# Chemical and Enzymatic Synthesis of DisialylGb5 and Other Sialosides for Glycan Array Assembly and Evaluation of Siglec-Mediated Immune Checkpoint Inhibition

**DOI:** 10.3390/molecules30112264

**Published:** 2025-05-22

**Authors:** Kuo-Shiang Liao, Yixuan Zhou, Cinya Chung, Chih-Chuan Kung, Chien-Tai Ren, Chung-Yi Wu, Yi-Wei Lou, Po-Kai Chuang, Balázs Imre, Yves S. Y. Hsieh, Chi-Huey Wong

**Affiliations:** 1 Genomics Research Center, Academia Sinica, Taipei 115, Taiwan; shiang.cgt@gmail.com (K.-S.L.); zhouyixuan1984@outlook.com (Y.Z.); cinyachung@gmail.com (C.C.); kung061283@gmail.com (C.-C.K.); a0922720496@gmail.com (C.-T.R.); cywu@rockbiomedical.com (C.-Y.W.); lou.yi.wei@gmail.com (Y.-W.L.); 2 Department of Chemistry, The Scripps Research Institute, San Diego, CA 92037, USA; pokaipkc@gmail.com; 3 School of Pharmacy, Taipei Medical University, Taipei 110, Taiwan; lalazsimre@tmu.edu.tw (B.I.); yvhsieh@tmu.edu.tw (Y.S.Y.H.); 4 Division of Glycoscience, Department of Chemistry, School of Engineering Sciences in Chemistry, Biotechnology and Health, Royal Institute of Technology (KTH), AlbaNova University Center, SE10691 Stockholm, Sweden

**Keywords:** sialyl SSEA-4, programmable, chemoenzymatic synthesis, glycolyl sialic acid, immune checkpoint

## Abstract

Aberrant glycosylation, especially sialylation, on cell surface is often associated with cancer progression and immunosuppression. Over-sialylation of stage-specific embryonic antigen-4 (SSEA-4) to generate disialylGb5 (DSGb5) was reported to trigger Siglec-7 recognition and suppress NK-mediated target killing. In this study, efficient chemo-enzymatic and programmable one-pot methods were explored for the synthesis of DSGb5 and related sialosides for assembly of glycan microarrays and evaluation of binding specificity toward Siglecs-7, 9, 10, and 15 associated with immune checkpoint inhibition. The result showed weak binding of DSGb5 to these Siglecs; however, a truncated glycolyl glycan was identified to bind Siglec-10 strongly with a dissociation constant of 50 nM and exhibited a significant inhibition of Siglec-10 interacting with breast cancer cells.

## 1. Introduction

Aberrant sialylation has been recognized as a hallmark of tumorigenesis. An example of such a change is the differential expression of glycosyltransferases in cancer cells to generate tumor-associated carbohydrate antigens (TACAs) [1]. Another example is the over-sialylation of glycoproteins or glycolipids on cancer cells to enhance interaction with specific sialic acid-binding immunoglobulin-type lectins (Siglecs) on immune cells, resulting in a suppression of immune response against cancer cells, a process called immune checkpoint inhibition [2,3]. It has been demonstrated that certain sialylated glycolipids, like stage-specific embryonic antigen (SSEA-4) [4,5], exclusively expressed on cancer cells are associated with cancer progression [6,7,8,9] and the expression level correlates with poor survival of cancer patients [10]. Interestingly, SSEA-4 could be further sialylated by the enzyme ST6GalNAc6 to form disialylGb5 (DSGb5), which was first found and downregulated in renal cancer [11]. In addition, DSGb5 and ST6GalNAc6 were reported to be downregulated in colon cancer [12], resulting in the decreased expression of disialyl LeA and increased expression of sialyl LeA and SSEA-4, which was linked to the signaling pathway that maintained the survival of tyrosine kinase inhibitor (TKI)-resistant cancer cells [13,14,15,16,17,18,19,20]. Moreover, recent development of anti-SSEA-4 antibodies [10], Globo-H cancer vaccines [21,22,23], and studies of globo-series glycan biosynthesis [4,24] all point to SSEA-4 as a target for new cancer therapies. On the other hand, specific Siglecs on immune cells may interact with specific sialylated glycans on cancer cells to trigger immune suppression [2,3]. A recent report showed that DSGb5 on renal cancer cells interacted with Siglec-7 on NK cells and downregulated their cytotoxicity [25]. This report prompted us to further investigate the binding specificity of DSGb5 toward Siglecs and compared to that of other tumor-associated carbohydrate antigens (TACAs). Since DSGb5 is not readily available [26], we first explored methods for the synthesis of DSGb5, including the programmable one-pot method and the enzymatic method using sialyltransferases that catalyzed the sialylation of SSEA-4 to form DSGb5. We then combined the synthetic glycans and other sialylated TACAs [21,27,28,29,30,31] and N-glycans to construct a sialylo–glycan array to profile the binding specificity of Siglecs, especially Siglec-7, Siglec-9, Siglec-10, and Siglec-15, to assess their role in immune checkpoint inhibition.

## 2. Results and Discussion

### 2.1. The mRNA Level of ST6GALNAC6 in Brain, Breast, Colon, Lung, Pancreas, and Prostate Cancers Decreased Compared to Normal Cells

**Figure 1:**
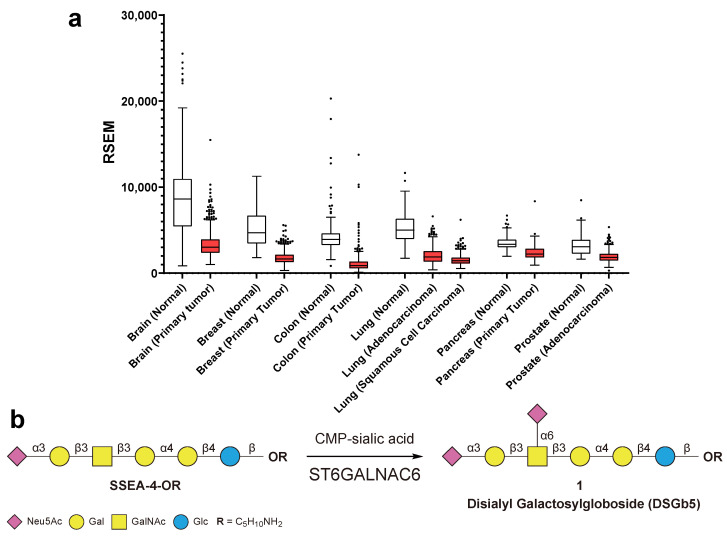
(**a**) The RSEM gene expression values of ST6GalNAc6 mRNA in brain, breast, colon, lung, pancreas, and prostate cancer cells (red box) and normal cells (white box). (**b**) Synthesis of DSGb5 by ST6GalNAc6-catalyzed sialylation of SSEA-4.

We previously showed that the elevated expression of SSEA-4 on cancer cells was caused by increased expression of β3GalT5 and ST6Gal2 [10]. Here, we compare the mRNA levels of globo-series glycotransferases in normal and cancer tissues with large-scale RNA-Seq transcriptome analyses from The Cancer Genome Atlas (TCGA) TARGET and GTEx (The Genotype-Tissue Expression) datasets. We observed a statistically significant decrease in the mRNA level of ST6GalNAc6 (Figure 1) in all cancers examined, and the decrease was significant at an early stage, while there was no obvious change in the mRNA levels of β4GalT, β3GalNT1, β3GalT5, and ST3Gal2, suggesting that downregulation of ST6GalNAc6 expression in the early stage of tumorigenesis correlated with increased expression of SSEA-4 in cancers.

### 2.2. Synthesis of DSGb5 and Other Sialylated Derivatives

**Scheme 1:**
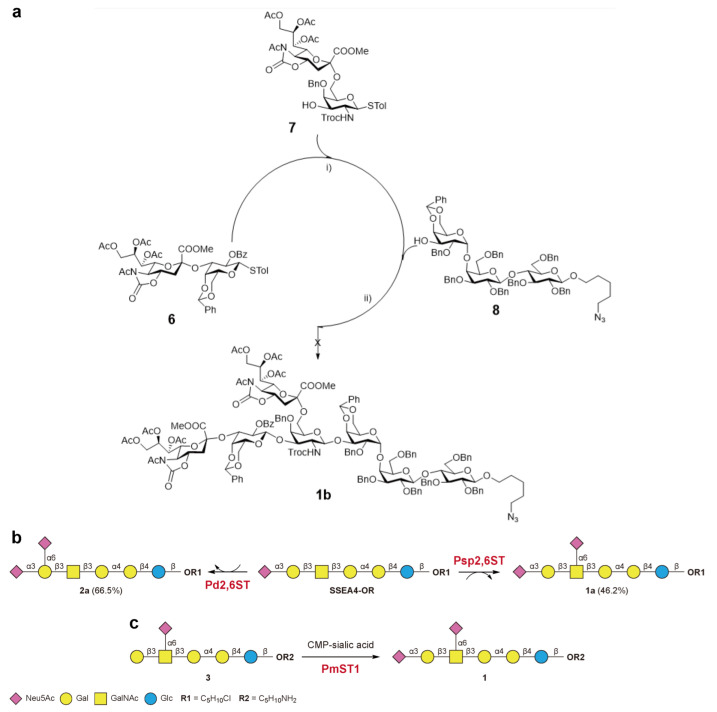
(**a**) Programmable one-pot synthesis of DSGb5 glycan (i) NIS, TfOH, MS 4 Å, CH_2_Cl_2_, −78 °C, 2 h; (ii) NIS, TfOH, MS 4 Å, CH_2_Cl_2_, −40 °C, 2 h. (**b**) Enzymatic sialylation of SSEA-4 with Pd2,6ST and Psp2,6ST. (**c**) Synthesis of DSGb5 by enzymatic α2,3-sialylation of sialyl SSEA3. NmCSS, CMP-sialic acid synthetase from *Neisseria meningitidis*; PmST1, α2,3-sialyltransferase from *Pasteurella multocida*.

We first explored the programmable one-pot method [32] for the synthesis of DSGb5 glycan. We designed two sialylated disaccharide building blocks, one with α2,3- and the other with α2,6-linkage, with distinct relative reactivity for the one-pot synthesis to give the product in moderate yield (Scheme 1a. see details in Supporting Information). We then explored the enzymatic method to sialylate SSEA-4 using the enzyme N-acetylgalactosamine α2,6-sialyltransferase VI from *Photobacterium damsela* (Pd2,6ST) [33,34]. However, Neu5Ac was transferred to the C-6 position of terminal galactose (compound **2a**) instead of the internal GalNAc (Scheme 1b). We next tested the α2,6-sialyltransferase from *Photobacterium* sp. (Psp2,6ST), and to our delight, DSGb5 glycan (compound **1a**) was obtained in 46% yield (Scheme 1b). We also designed a new enzymatic route to DSGb5 glycan that involves α2,6-sialylation of SSEA-3, followed by α2,3-sialylation at terminal galactose using a recombinant *Pasteurella multocida* α2,3-sialyltransferase (PmST1) (Scheme 1c). After purification with an ion-exchange column, the product was obtained in 10% yield, and the NMR spectrum was identical to compound **1**, except differences in the linker moiety at the reducing end. This result further confirmed that DSGb5 glycan can be obtained by α2,6-sialylation of SSEA-4 with Psp2,6ST or by α2,3-sialylation of sialyl SSEA-3 with PmST1.

To further optimize the yield of DSGb5 glycan, we overexpressed human ST6GalNAc6 from HEK 293 cells. DSGb5 (compound **1**) was enzymatically synthesized via human ST6GalNAc6 from SSEA-4 glycan (see Materials and Methods). The result showed that human ST6GalNAc6 was more efficient and DSGb5 glycan was obtained in 82.1% yield (Figure 1).

Due to the different specificities of Pd2,6ST and Psp2,6ST, we further explored the sialylation of a range of globo-series glycan related acceptors to better understand their acceptor specificity and to expand the structural repertoire of sialylated glycans for our glycan array study of Siglecs. The obtained products and yields are summarized in Table S1. We found that Pd2,6ST accepted Gb4, Gb5, and SSEA-4 glycans with terminal Gal/GalNAc residues, whereas Gb3 and Globo-H glycans could not be sialylated by this enzyme. Previous studies also showed a lower catalytic efficiency of Pd2,6ST when the α-linked terminal GalNAc of GalNAcαAA or GalNAcαSer was used as an acceptor [33,35,36,37]. Similar results were found in the case of Gb3 and Globo-H glycans, in which the α-linked terminal Gal or Fuc may have hindered the activity of Pd2,6ST. Similarly, Psp2,6ST exhibited no reactivity towards Gb3 glycan, while its activity towards Gb5 glycan was lower than that of Pd2,6ST, as the former not only transferred Neu5Ac to the terminal Gal residue to afford **5a** but also sialylated GalNAc to afford disialylated oligosaccharide **16**, with 15% isolated yield. In addition, a previous study showed that Pd2,6ST catalyzed the transfer of Neu5Ac to the terminal Gal and/or GalNAc residues of the disaccharide acceptor Galβ1-3GalNAc [38]. However, only the terminal Gal was sialylated by the enzyme to afford hexasaccharide **5a** as the sole product. Interestingly, the presence of Fuc did not affect the Psp2,6ST-catalyzed sialylation of Globo-H glycan at terminal Gal to form compound **17** in 33% isolated yield. Moreover, Psp2,6ST was found to sialylate the GalNAc residue of Globo-H glycan to form compound **8** albeit in trace amounts, which was later characterized by MS/MS analysis. The sialylation of SSEA-4 glycan by Psp2,6ST showed high regioselectivity, probably due to the α2-3 linked Neu5Ac in the terminal Gal residue.

### 2.3. Glycan Array Analysis of Siglec-7, Siglec-9, Siglec-10, and Siglec-15 Binding to DSGb5 Glycan and Other Cancer-Associated Carbohydrate Antigens

**Figure 2:**
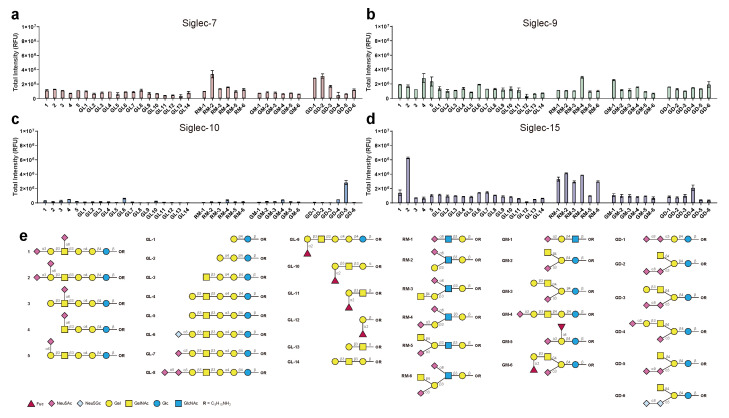
Glycan array profiling of commercially available Siglecs with TACAs. (**a**) Siglec-7. (**b**) Siglec-9. (**c**) Siglec-10. (**d**) Siglec-15. (**e**) TACAs used in this study. Siglec concentrations = 10 µg/mL.

With these glycans in hand, we then created a glycan array on NHS-activated glass slides and investigated the binding specificity of several Siglecs. This array, including the glycans associated with tumor-associated glycolipids, like globo-series and RM2-related glycans, gangliosides [25], and other TACAs, was used to profile the binding specificities of Siglec-7, -9, -10, and -15. The results showed that SSEA-4 glycan (compound **GL-7**) and DSGb5 (compound **1**) lack significant binding to these four Siglecs, while Siglec-15 showed binding to the disialylated SSEA-3 glycan (Gb5), compound **2** (Figure 2d). The glycans of gangliosides GD2 (compound **GD-2**) and GD3 (compound **GD-1**) showed interaction with Siglec-7 [39,40,41,42,43,44,45,46,47]. We also detected a significant binding of Siglec-7 to the mono-sialylated compound **RM-2** (Figure 2a). However, Siglec-9 showed a stronger binding than Siglec-7 to the DSGb5 and **RM-4** glycans. On the other hand, Siglec-10 showed a significant binding to the unnatural **GD-5** glycan with α2,9- sialylation, and Siglec-15 recognized the SSEA-3 derivative with two sialic acids linked to the terminal galactose, compound **2** (Figure 2d).

### 2.4. Binding of Siglecs Toward Sialylated N-Glycans

**Figure 3:**
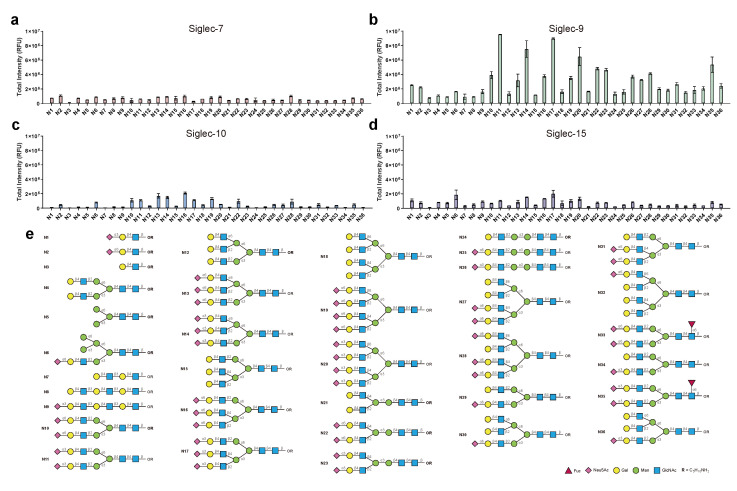
Profiling of (**a**) Siglec-7, (**b**) Siglec-9, (**c**) Siglec-10, and (**d**) Siglec-15 with *N*-glycan array. Siglec-9 significantly interacted with terminal α2,3-linked sialic acids of complex-type glycans compared to the α2,6-linked sialosides and the binding affinity increases with increase in antenna (**N11** vs. **N10**, **N14** vs. **N13**, **N17** vs. **N16**, **N20** vs. **N19**). In addition, the core fucose does not have a significant effect on binding (**N11** vs. **N35**). On the other hand, Siglec-10 prefers the *N*-sialosides with terminal α2,6-linked sialic acid. (**e**) *N*-glycans used in this study. Siglec concentrations = 10 µg/mL.

Since the glycans of cancer-associated glycolipids did not show significant binding to the Siglecs, we turned our attention to an array of sialylated *N*-glycans generated by us previously [28,29]. None of the *N*-glycans in our library showed any significant binding to Siglec-7 (Figure 3a). Interestingly, Siglec-9 recognized the glycans with terminal α2,3-sialylation (**N11**, **N14**, **N17**, and **N20**). On the other hand, the same type of *N*-glycans with α2,6-sialylaton (**N10** vs. **N11**, **N13** vs. **N14**, **N16** vs. **N17**, **N19** vs. **N20**) exhibited lower binding affinities. We also found that increasing the number of sialic acid residues in the α2,3-sialylated biantennary, triantennary, and tetraantennary *N*-glycans increased the binding affinity to Siglec-9. In contrast, no such trend was found in the case of α2,6-sialylated *N*-glycans (Figure 3b). The 2,4,2-triantennary *N*-glycan showed a stronger binding to Siglec-9 than the 2,2,6-branched one, irrespective of terminal sialic acid with α2,3- or α2,6- linkage (**N17** vs. **N16**, **N14** vs. **N13**). Interestingly, Siglec-9 did not show any significant interaction with complex type *N*-glycans with LacNAc repeats terminated with α2,3- or α2,6- siaylation. In addition, the core fucose did not affect binding to Siglec-9 as shown in the case of **N11** and **N35**. Contrary to what we observed in the case of Siglec-9, Siglec-10 preferred *N*-glycans with terminal α2,6-sialylation instead of α2,3-sialylation (**N13** vs. **N14**, **N16** vs. **N17**, and **N19** vs. **N20**). Moreover, an increasing number of terminal sialic acid groups led to a stronger binding (**N10** vs. **N11**, **N13** vs. **N14**, **N16** vs. **N17**, and **N19** vs. **N20** in Figure 3c). Siglec-15 exhibited a similar binding pattern to that of Siglec-9 toward *N*-glycans, favoring α2,3-sialosides, albeit with somewhat lower binding affinity (Figure 3d).

### 2.5. Inhibition of Siglec-10 Binding to CD24 on Breast Cancer Cells with Synthetic Glycans

**Figure 4:**
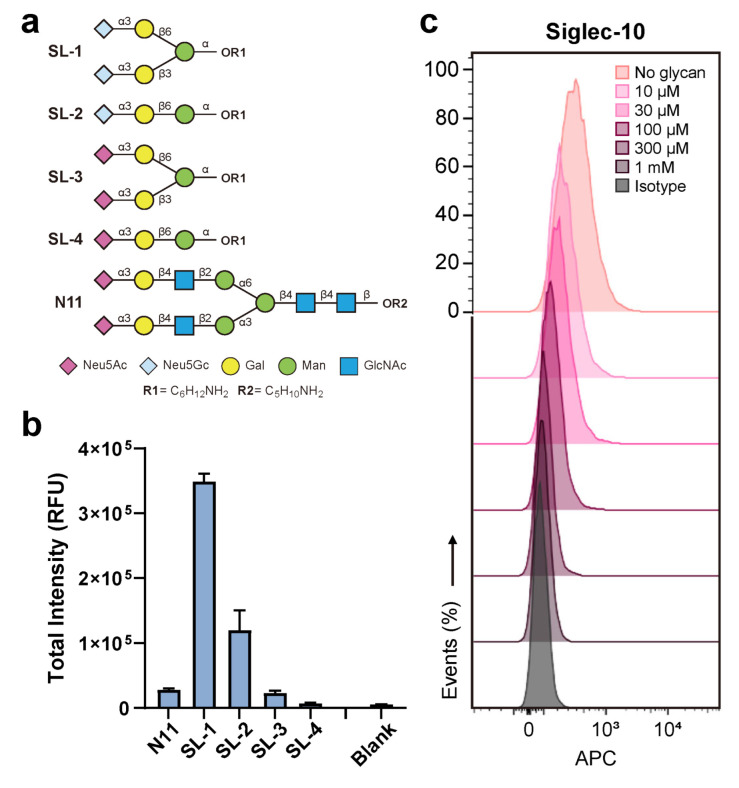
(**a**) Glycan candidates for Siglec-10 inhibition. (**b**) Siglec-10 binding signals of glycan candidates on glycan array. Binding of Siglec-10 (2.5 µg/mL) towards glycans on total glycan array (printed at 100 µM). Secondary antibody: Donkey anti-human IgG, Fcγ fragment specific, Alexa Fluor^®^ 647-conjugated. Error bars: standard deviation. (**c**) Inhibition of Siglec-10 binding to MCF-7 at various concentrations of SL-1.

A recent report suggested that CD24, a highly glycosylated glycosylphosphatidylinositol (GPI)-anchored protein [48,49], is overexpressed on various tumor cells [50] and is a ligand for Siglec-10. CD24 was also found to enhance the metastatic potential of malignant cells [51]. Human Siglec-10 is widely expressed in hematopoietic cell types [52], such as B and T cells, monocytes eosinophils, NK cells and macrophages [53,54]. The Siglec-10-CD24 interaction that led to immune suppression has been considered as a promising target for cancer immunotherapy [53,54,55,56,57]. The interaction of tumor CD24 and macrophage Siglec-10 inhibits phagocytosis and increases tumor growth and cell survival [53]. Removal of α2,3- and α2,6-linked sialic acids from CD24 by *Vibrio cholerae* neuraminidase significantly reduced binding to Siglec-10 [58]. Therefore, identification of Siglec-10 ligand may aid in the discovery of potential cancer biomarkers and the development of possible cancer therapy. Human Siglec-10 is a membrane-bound protein [59,60] that recognizes ɑ2,6-linked sialosides on cancer cells. In this study, we identified a strong ligand (**SL-1**) of Siglec-10 using our array and the commercially available array from RayBiotech (Figure S1), a bi-antennary glycan terminated with two α2,3-glycolylneuraminic acid with a dissociation constant of 0.050 ± 0.011 µM (Figure 4a and Figure S2). The binding affinity of Siglec-10 towards **SL-1** was better than other glycans with terminal Neu5Ac and the number of Neu5Gc-modified branches also influenced the binding affinity (**SL-1** > **SL-2**).

To test whether **SL-1** could compete with human breast cancer cell line MCF-7 over Siglec-10 binding, **SL-1** at different concentrations was added to a mixture of Siglec-10 and MCF-7 cells, and Siglec-10 binding signals were observed using flow cytometry. As shown in Figure 4c, binding of Siglec-10 to MCF-7 cells decreased as the concentration of glycan **SL-1** increased, suggesting that **SL-1** is a competitive inhibitor of Siglec-10 binding to MCF-7 cells. Competition analysis of Siglec-10 binding to glycans vs. MCF-7 cells was also performed with glycans **SL-2**, **SL-3**, **SL-4** and **N11**, respectively, at 100 µM. As shown in Figure S2, **SL-1** still had the strongest inhibition of Siglec-10 binding to MCF-7 cells and decreased in the order **SL-1** > **SL-2** > **SL-3** > **N11** > **SL-4**.

With **SL-2**, which contained only one of the branches of **SL-1**, Siglec-10 had a dissociation constant of 0.110 ± 0.041 µM. For **SL-3**, **SL-4**, and **N11**, the dissociation constants were similar (~0.176 µM); however, the fluorescent signals were very weak, and significantly lower than **SL-1** and **SL-2**. These results showed that Siglec-10 preferred glycolylneuraminic acid (**SL-1** and **SL-2**) over acetylneuraminic acid (**SL-3**, **SL-4** and **N11**).

The binding affinity also increased as the number of sialic acids increased (**SL-1** > **SL-2**). The multivalent glycans displayed on the microarray can be viewed as a mimic of cell-surface glycans confined in a small area. On the other hand, glycans used in the inhibition experiment in flow cytometry were dispersed in the solution as monomers. The binding affinity between glycans and Siglec-10 in solution should be lower than on microarrays. Therefore, to achieve better inhibitory effects, the interaction of **SL-1** to Siglec-10 should be increased. Since Siglec-10 is expressed on immune cells as multivalent glycoproteins, not as free form in solution, while MCF-7 cells are adherent cells, the interaction between two cells is more complex than that based on glycan array profiling. Work is in progress to identify better inhibitors of Siglec-10-mediated immune checkpoint suppression.

## 3. Materials and Methods

### 3.1. General Chemical Synthesis

All chemicals were purchased as reagent grade and used without further purification. Anhydrous dichloromethane (CH_2_Cl_2_) was purchased from a commercial source without further distillation. Pulverized Molecular MS 4 Å (Sigma-Aldrich, St. Louis, MO, USA) for glycosylation were activated by heating at 350 °C for 10 h. Reactions were monitored by analytical thin-layer chromatography (TLC) in EM silica gel 60 F254 plates and visualized under UV (254 nm) and/or by staining with acidic ceric ammonium molybdate or p-anisaldehyde. Flash chromatography was performed on silica gel (Millipore, Burlington, MA, USA) of 40–63 μm particle size. 1H NMR spectra were recorded on a Bruker AVANCE 600 (600 MHz) spectrometer at 25 °C. Chemical shift (in ppm) was assigned according to CDCl_3_ (δ = 7.24 ppm) and D_2_O (δ = 4.80 ppm). C-13 NMR spectra were obtained with Bruker AVANCE 600 spectrometer and were calibrated with CDCl_3_ (δ = 77.00 ppm). Coupling constants (J) are reported in hertz (Hz). Chemical shifts measurements are reported in delta (δ) units, and splitting patterns are described as singlet (s), doublet (d), triplet (t), quartet (q), or multiplet (m). Coupling constants (J) are reported in Hertz (Hz). High resolution ESI mass spectra were recorded on a Bruker Daltonics or Bruker Bio-TOF III spectrometer (Bruker, Billerica, MA, USA). MALDI-TOF spectra were recorded on Bruker Ultraflex II sepctrameter (Bruker).

### 3.2. Enzyme Preparations

Codon-optimized genes for pET-15b-Pd2,6ST, pET-22b-Psp2,6ST, and pET-23a-PmST1 were synthesized by GenScript™. To construct the expression plasmid for soluble ST6GalNAc6, the pHEK293 Ultra Expression Vector I (TAKARA) was linearized with XhoI and ligated with the gene encoding soluble ST6GalNAc6 (V67–T333) fused to a C-terminal FLAG tag, using the NEBuilder^®^ HiFi DNA Assembly Cloning Kit (NEB). The resulting plasmid was sequence-verified.

For bacterial expression of recombinant Pd2,6ST, Psp2,6ST, and PmST1, an overnight culture of BL21(DE3) was inoculated into 1 L Terrific Broth (Sigma-Aldrich) at a 1:50 (*v/v*) ratio in a 2.5 L baffled flask (Thomson, Carlsbad, CA, USA). When the culture reached an OD_600_ of 0.8–1.0, protein expression was induced with 1 mM IPTG and incubated at 16 °C for 24 h. The cells were harvested, lysed using Bugbuster Protein Extraction Reagent (Millipore, Burlington, MA, USA), and the supernatant containing 6 × His-tagged proteins was purified using IMAC (Cytiva, Marlborough, MA, USA). The column was washed and eluted with eight column volumes of 50 mM MOPS, 300 mM NaCl, and either 20 mM or 300 mM imidazole (pH 7.5). The fractions were analyzed by SDS-PAGE followed by Coomassie Blue staining.

For mammalian expression of ST6GalNAc6, Expi293 cells were transfected according to the manufacturer’s instructions, with a starting cell density of 1.5 million cells/mL. After 72 h, the culture supernatant was collected, filtered, and passed through anti-FLAG M2 agarose resin (Millipore). The resin was equilibrated with 10 column volumes of TBS buffer and 20 mM HEPES buffer containing 300 mM NaCl (pH 7.0) before loading. After binding, the resin was washed with 10 column volumes of the same buffer, and the protein was eluted using 8 column volumes of 0.1 M Tris-glycine (pH 3.0). The elution was immediately neutralized by adding 1/15 volume of 1 M Tris-HCl (pH 9.0). The enzyme was then buffer-exchanged and concentrated using an Amicon Ultra centrifugal filter unit (Millipore) with an appropriate molecular weight cutoff. Protein concentration was determined using the Pierce^TM^ BCA Protein Assay Kit (Thermo Fisher Scientific, Waltham, MA, USA).

### 3.3. Enzymatic Synthesis of DSGb5 via Human ST6GalNAc6

The enzymatic reaction was subsequently performed in a reaction mixture containing 100 mM sodium cacodylate (pH 6.0), 10 mM MgCl_2_, 1.6 mM CMP-sialic acid, 3 µg of recombinant human ST6GalNAc6, and 0.08 mM SSEA-4 glycan as acceptor substrate. The reaction mixture was incubated at 37 °C overnight with gentle shaking. The product was then isolated by solid phase extraction with HyperSep™ Hypercarb™ SPE Cartridges (Thermo Fisher Scientific) and analyzed by porous graphitic carbon liquid chromatography/tandem mass spectrometry (PGC-LC-MS/MS) using a Hypercarb™ column (Thermo Fisher Scientific).

### 3.4. Glycan Microarray

Glycan microarrays were fabricated by printing 100 µM amine-containing glycans in printing buffer (300 mM sodium phosphate buffer (pH 8.5) with 0.01% Triton X-100) onto NHS-activated glass slides (Nexterion^®^ Slide H, SCHOTT, Mainz, Germany) as previously described [61]. Recombinant human Siglec-hFc-fusion proteins (Siglec-7, 9, 10, 15) were purchased from R&D Systems. The microarrays were blocked with SuperBlock Blocking Buffer in PBS (Thermo Fisher Scientific) at RT for 1 h and washed with 1 × PBS buffer containing 0.05% Tween-20 (PBST). Siglecs were diluted with PBST containing 30 mg/mL BSA (PBST-BSA). The Siglec samples were added to the microarray and incubated at RT for 1 h. The microarrays were washed with PBST, then added with DyLight™ 649 modified anti-human IgG antibody (donkey anti-human IgG, Fcγ fragment specific, DyLight™ 649, Jackson ImmunoResearch, West Grove, PA, USA) for 1 h in the dark. Finally, the microarrays were washed in PBST, milliQ, and dried. The microarrays were scanned at 635 nm using a GenePix 4300A Microarray Scanner (Molecular Devices, San Jose, CA, USA), and the fluorescence intensities were analyzed by the GenePix Pro 7.0 software (Molecular Devices).

The dissociation constants of Siglec-10 binding to glycans SL-1, SL-2, SL-3, SL-4 and N11 were determined using glycan microarray. Concentrations of amine-modified glycans ranging from 10 µM to 400 µM were printed onto NHS-modified glass slides [62], then treated with various concentrations of Siglec-10 coupled with Alexa Fluor^®^ 647 modified anti-human IgG antibody (donkey anti-human IgG, Fcγ fragment specific, Alexa Fluor^®^ 647, Jackson ImmunoResearch) at 4 °C overnight. After incubation, the microarray slides were washed with PBST, milliQ, and then dried. The microarrays were excited with 635 nm lasers and scanned using a GenePix 4300A Microarray Scanner, and the fluorescence intensities were analyzed by the GenePix Pro 7.0 software. Data analysis and Langmuir isotherms fitting was performed using PRISM (GraphPad, Boston, MA, USA) [61,62]. The microarrays were excited with 635 nm lasers and scanned using a GenePix 4300A Microarray Scanner, where the fluorescence intensities were analyzed using the GenePix Pro 7.0 software. Fluorescence intensities were plotted out against Siglec-10 concentrations to give a set of curves which were analyzed as Langmuir isotherms, assuming the reaction reached equilibrium,

$$\frac{F_{max}[P]}{\left[P\right]+K_{D}},$$

where *F_max_* is the maximum fluorescence intensity, [*P*] is the total Siglec-10 concentration, and *K_D_* is the equilibrium dissociation constant for surface glycan and Siglec-10 binding.

### 3.5. Cell Culture

The human breast cancer cell line MCF-7 (HTB-22, the American Type Culture Collection, Manassas, VA, USA) was cultured in RPMI 1640 medium (Thermo Fisher Scientific) supplemented with 10% fetal bovine serum, nonessential amino acids (Thermo Fisher Scientific) and 1 × antibiotic-antimycotic (Thermo Fisher Scientific) [63]. The cells were incubated at 37 °C with 5% CO_2_ and humidified atmosphere control. The culture medium was changed every 3 to 4 days.

### 3.6. Flow Cytometry

MCF-7 cells were detached from the dish surface through trypsinization, and washed with ice-cold FACS buffer (1% FBS in 1 × DPBS with 0.1% Sodium Azide) before staining with the following antibodies for respective experimental purposes: (1) For visualization of CD24 on the cell surface, cells were stained with anti-CD24 antibody, CD24 Monoclonal Antibody (eBioSN3 (SN3 A5-2H10)), PE (Invitrogen); Mouse IgG1 kappa Isotype Control (P3.6.2.8.1), PE (Thermo Fisher Scientific) was used as isotype control. (2) In Siglec-10 binding/inhibitory experiments, 5 × 10^5^ MCF-7 cells were incubated with 10 µg/mL Siglec-10, along with different concentrations of SL-1 glycan (0, 0.01 to 1 mM) in FACS buffer and incubated at 4 °C for 1 h. The cells were washed with FACS buffer before adding Alexa Flour^®^ 647-conjugated anti-hFc antibody for Siglec-10 staining. Flow cytometry was performed on FACSCanto flow cytometer (BD Bioscience, Franklin Lakes, NJ, USA).

### 3.7. Commercial Glycan Array

Profiling of recombinant Siglec-10 was performed on commercial Glycan Array 300 (RayBiotech, Peachtree Corners, GA, USA) by the Academia Sinica Glycoscience Core Facility. Siglec-10 samples were dialyzed and labeled with biotin according to the protocol provided by the manufacturer. The glycan array slide was blocked, washed, and biotin-labeled proteins were added for incubation with the reagents provided in the kit. Cy3 equivalent dye-conjugated streptavidin was used to visualize the binding signals. The glycan arrays were excited at 532 nm laser and scanned using a GenePix 4300A Microarray Scanner.

## 4. Conclusions

It is known that cancer cells utilize the Siglec interacting pathway to evade immune cell-mediated cytotoxicity. In this study, we describe the development of facile and scalable chemo-enzymatic strategies for the synthesis of DSGb5 and sialylated derivatives. These sialylated glycans and the glycans of cancer-associated glycolipids were used to create glycan microarrays to evaluate their binding towards recombinant human Siglecs, including Siglec-7, Siglec-9, Siglec-10, and Siglec-15. It was found that SSEA-4 and DSGb5 glycans had binding to Siglec-9 and the disialyl SSEA-3 glycan with disialylated terminal Gal (compound **2**) showed binding to Siglec-9 and Siglec-15. However, these Siglecs exhibited better binding to sialylated N-glycans and the binding affinity and specificity were strongly influenced by the α2,3- or α2,6-linkage of terminal sialic acid, with Siglec-9 in favor of the α2,3-linkage and Siglec-10 the α2,6-linkage. Notably, the complex-type N-glycans are better ligands for Siglec-9 and Siglec-10 and the binding increases with increasing number of terminal sialic acids. We also found that the N-glycolylneuraminic acid derivatives are better ligands for Siglec-10, especially the truncated biantennary glycan SL-1, which exhibited a significant inhibition of macrophage-mediated phagocytosis.

## Data Availability

All study data are included and available in the article and/or in the Supporting Information (SI). The raw data supporting the conclusions of this article will be made available by the authors upon request.

## Supplementary Materials

The following supporting information can be downloaded at https://www.mdpi.com/article/10.3390/molecules30112264/s1, detailed description of synthesis of DSGb5 and related sialosides and their characterization, including NMR data; Scheme S1–S3, Table S1–S2, Figure S1–S3, and References [1,2,3,4,5,6,7,8,9,10,11,12] are cited in the Supplementary Materials.
