# Supplementary material for: Chemical and Enzymatic Synthesis of DisialylGb5 and Other Sialosides for Glycan Array Assembly and Evaluation of Siglec-Mediated Immune Checkpoint Inhibition
Source: Molecules. 2025 May 22;30(11):2264. doi: 10.3390/molecules30112264 (PMC12156072; doi:10.3390/molecules30112264)
Supplement: Supplementary file 1 [file molecules-30-02264-s001.zip › molecules-3614457-supplementary.pdf]

Supporting Information

Article

Chemical and Enzymatic Synthesis of DisialylGb5 and Other Sialosides for Glycan Array Assembly and Evaluation of Siglec-Mediated Immune Checkpoint Inhibition

Kuo-Shiang Liao [1,†], Yixuan Zhou [1,†], Cinya Chung [1,†], Chih-Chuan Kung [1], Chien-Tai Ren [1], Chung-Yi Wu [1], Yi-Wei Lou [1], Po-Kai Chuang [2], Balázs Imre [3], Yves S. Y. Hsieh [3,4] and Chi-Huey Wong [1,2,*]

[1] Genomics Research Center, Academia Sinica, Taipei 115, Taiwan; shiang.cgt@gmail.com (K.-S.L.); zhouyixuan1984@outlook.com (Y.Z.); cinyachung@gmail.com (C.C.); kung061283@gmail.com (C.-C.K.); a0922720496@gmail.com (C.-T.R.); cywu@rockbiomedical.com (C.-Y.W.); lou.yi.wei@gmail.com (Y.-W.L.)
[2] Department of Chemistry, The Scripps Research Institute, San Diego, CA 92037, USA; pokaipkc@gmail.com
[3] School of Pharmacy, Taipei Medical University, Taipei 110, Taiwan; lalazsimre@tmu.edu.tw (B.I.); yvhsieh@tmu.edu.tw (Y.S.Y.H.)
[4] Division of Glycoscience, Department of Chemistry, School of Engineering Sciences in Chemistry, Biotechnology and Health, Royal Institute of Technology (KTH), AlbaNova University Center, SE10691 Stockholm, Sweden
* Correspondence: wong@scripps.edu
† These authors contributed equally to this work.

Academic Editor: Joachim Erich Thiem

Received: 14 April 2025
Revised: 14 May 2025
Accepted: 19 May 2025
Published: 22 May 2025

Citation: Liao, K.-S.; Zhou, Y.; Chung, C.; Kung, C.-C.; Ren, C.-T.; Wu, C.-Y.; Lou, Y.-W.; Chuang, P.-K.; Imre, B.; Hsieh, Y.S.Y.; et al. Chemical and Enzymatic Synthesis of DisialylGb5 and Other Sialosides for Glycan Array Assembly and Evaluation of Siglec-mediated Immune Checkpoint Inhibition. *Molecules* **2025**, *30*, x. https://doi.org/10.3390/xxxxx

Copyright: © 2025 by the authors. Submitted for possible open access publication under the terms and conditions of the Creative Commons Attribution (CC BY) license (https://creativecommons.org/licenses/by/4.0/).

Synthesis of Acceptor 7

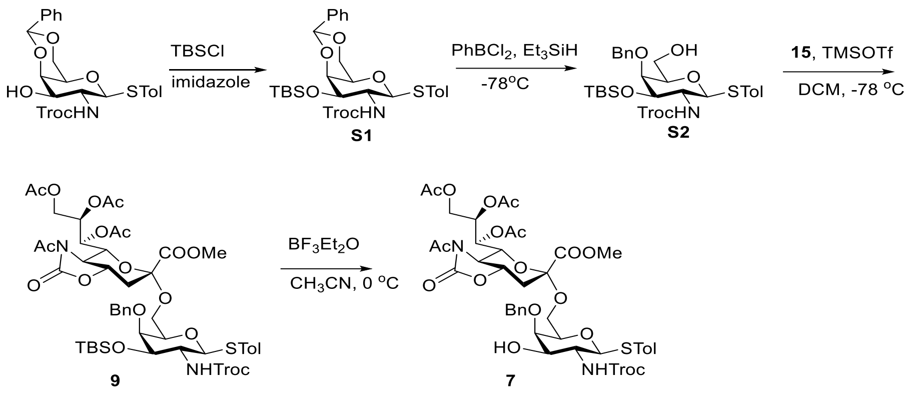

A solution of 2,2,2-trichloroethyl((2*S*,4a*R*,6*S*,7*R*,8*R*,8a*R*)-8-hydroxy-2-phenyl-6- (*p*-tolylthio)hexah-ydropyrano[3,2-*d*][1,3]dioxin-7-yl)carbamate [1] (1.0 g, 1.8 mmol), tert-Butyldimethylsilyl chloride (0.41 g, 2.7mmol) and imidazole (0.25 g, 3.6 mmol) in dry DMF (10 mL) was stirred under argon at room temperature for overnight. After concentrating in *high vacuo*, the reaction mixture was washed with saturated aq. $NaHCO_3$. After drying over $MgSO_4$, the solution was filtered and concentrated in *vacuo*. The residue was purified by silica gel column chromatography (EtOAc/Hexane 1:2), to give the compound **S1** (1.09 g, 90%).

A solution of **S1** (1 g, 1.5 mmol), pulverized activated 4Å MS (2.0 $gmmol^{-1}$) in dry DCM (10 mL) was stirred under argon at room temperature for 2 h. The reaction was then cooled to -78°C followed by a slow addition of Et_3SiH (0.82 mL, 5.1 mmol) and $PhBCl_2$ (0.46 mL, 4.5 mmol). After being stirred for 1 h, the reaction mixture was neutralized by Et_3N (0.1 mL) and washed with saturated aq. $NaHCO_3$. After drying over $MgSO_4$, the solution was filtered and concentrated in *vacuo*. The residue was purified by silica gel column chromatography (EtOAc/Hexane 1:2), to give compound **S2** after drying (0.75 g, 75%). ^{1}H NMR (600 MHz, $CDCl_3$) δ 7.42 (d, *J* = 8.2 Hz, 2H), 7.37-7.34 (m, 5H), 7.05 (d, *J* = 8.0 Hz, 2H), 5.09 (d, *J* = 8.3 Hz, 1H), 5.03 (m, 2 H) 4.75 (d, *J* = 11.8 Hz, 1H), 4.69 (d, *J* = 11.8 Hz, 1H), 4.56 (d, *J* = 11.5 Hz, 1H), 4.08 (d, *J* = 9.3 Hz, 1H), 3.89-3.81 (m, 2 H),3.74 (d, *J* = 2.5 Hz, 1H), 3.59-3.57 (m, 2 H), 2.33 (s, 3 H), 0.94 (s, 9 H), 0.18 (s, 3 H), 0.13 (s, 3 H). ^{13}C NMR (150 MHz, $CDCl_3$) δ 153.7, 138.4, 137.7, 132.5, 129.7, 128.4, 127.7, 95.3, 86.2, 78.8, 74.9, 74.8, 62.4, 54.3, 25.8, 21.1, 18.0, -3.9, -5.0; HRMS (ESI-TOF, MNa^+) calcd for $C_{29}H_{40}Cl_3NO_6SSi$: 686.1303, found: 686.1330.

A solution of sialyl phosphate donor **15** [2-3] (0.75 g, 1.13 mmol), acceptor **S2** (0.5 g, 0.75 mmol) and pulverized activated 4Å MS (2.0 $gmmol^{-1}$) in dry DCM (12 mL) was stirred under argon at room temperature for 2 h. The reaction was then cooled to -78°C followed by a slow addition of TMSOTf (0.14 mL, 0.75 mmol) via micro-syringe. After being stirred for 1 h, the reaction mixture was neutralized by Et_3N (0.1 mL) and washed with saturated aq. $NaHCO_3$. After drying over $MgSO_4$, the solution was filtered and concentrated in *vacuo*. The residue was purified by silica gel column chromatography (EtOAc/Hexane 1:2), to give compound **9** (0.42 g, 50%). ^{1}H NMR (600 MHz, $CDCl_3$) δ 7.45 (d, *J* = 8.0 Hz, 2H), 7.35-7.33 (m, 4H), 7.30-7.28 (m, 1H),7.00 (d, *J* = 7.9 Hz, 2H),5.54 (dd, J_1 = 8.1 Hz, J_2 = 1.3 Hz, 2H), 5.48-5.46 (m, 2 H) 5.05 (d, *J* = 11.2 Hz, 1H), 4.94 (d, *J* = 9.9, 1H), 4.77 (d, *J* = 11.9 Hz, 1H), 4.67-4.60 (m, 3H), 4.46 (dd, J_1 = 12.2 Hz, J_2 = 2.4 Hz,1H), 4.09 (d, *J* = 8.7 Hz, 1H), 4.03-3.96 (m, 2H), 3.89-3.86 (m, 1H), 3.80-3.72 (m, 8H), 2.90 (dd, J_1 = 12.1 Hz, J_2 = 3.3 Hz,1H), 2.52 (s, 3H), 2.31 (s, 3H), 2.13-2.09 (m, 7H), 1.93 (s, 3H), 0.92 (s, 9H), 0.18 (s, 3H), 0.13 (s, 3H). ^{13}C NMR (150 MHz, $CDCl_3$) δ 172.2, 171.6, 170.2, 170.0, 168.3, 153.8, 153.7, 139.0, 137.0, 132.0, 129.9, 129.4, 128.1, 127.2, 100.1, 95.5, 86.2, 76.3, 76.0, 75.7, 74.9, 74.7, 71.9, 68.8, 64.5, 63.8, 59.0, 54.1, 36.7, 25.7, 24.8. 21.1, 21.0, 20.7, 17.9, -3.7, -5.0; HRMS (ESI-TOF, MNa^+) calcd for $C_{48}H_{63}Cl_3N_2O_{18}SSi$:1143.2524, found: 1143.2554.

A solution of compound **9** (0.42 g, 0.38mmol) in dry CH_3CN (5 mL) was cooled to 0 ^oC followed by addition BF_3•Et_2O (0.14mL, 1.37 mmol). After being stirred overnight at room temperature, the reaction mixture was washed with saturated aq. $NaHCO_3$. After drying over $MgSO_4$, the solution was filtered and concentrated in *vacuo*. The residue was purified by silica gel column chromatography (EtOAc/Hexane 1:2), to give compound **7** after drying (0.34 g, 90%). ^{1}H NMR (600 MHz, $CDCl_3$) δ 7.43 (d, *J* = 8.0 Hz, 2H), 7.37-7.35 (m, 4H), 7.33-7.32 (m, 1H), 7.01 (d, *J* = 7.7 Hz, 2H), 6.06 (d, *J* = 8.5 Hz, 1H), 5.57 (m, 1H), 5.39 (t, J_1 = J_2 = 7.8 Hz,1H), 4.93 (d, *J* = 12, 1H), 4.87 (d, *J* = 11.7 Hz, 1H), 4.78-4.74 (m, 2H), 4.69, (d, *J* = 9.4 Hz, 1H), 4.62, (d, *J* = 12 Hz, 1H), 4.05-3.98 (m, 3H), 3.88-3.82 (m, 7H), 3.72-3.66 (m, 2H), 2.91 (dd, J_1 = 12.2 Hz, J_2 = 2.8 Hz,1H), 2.54 (s, 3H), 2.43 (d, *J* = 8.6 Hz, 1H), 2.32 (s, 3H), 2.19 (s, 3H), 2.17 (s, 3H), 2.10 (m, 1H), 1.79(s, 3H). ^{13}C NMR (150 MHz, $CDCl_3$) δ 172.4, 172.2, 170.3, 169.9, 168.4, 155.1, 153.6, 138.5, 136.9, 131.4, 130.0, 129.4, 128.4, 127.7, 127.7, 100.6, 95.7, 86.6, 76.2, 75.8, 74.7, 74.6, 74.6, 74.4, 73.6, 71.3, 68.2, 64.9, 64.7, 59.0, 54.6, 53.3, 37.2, 24.8, 21.1, 21.0, 20.9, 20.5; HRMS (ESI-TOF, MH^+) calcd for $C_{42}H_{49}Cl_3N_2O_{18}S$:1007.1839, found: 1007.1868.

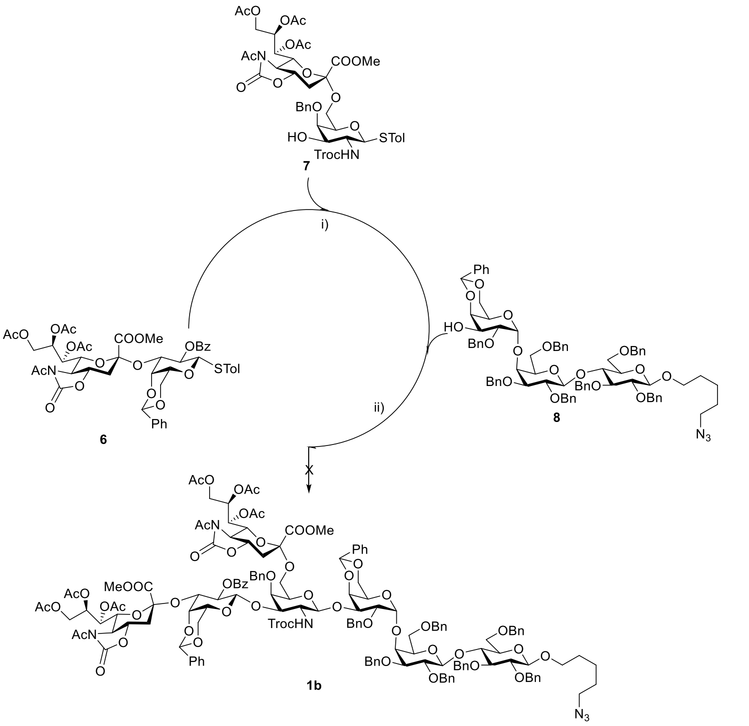

Scheme S1. Programmable one-pot synthesis of DSGb5 **(I)** NIS, TfOH, MS 4 Å, CH_2Cl_2, -78°C, 2 h; **(II)** NIS, TfOH, MS 4 Å, CH_2Cl_2, -40°C, 2 h

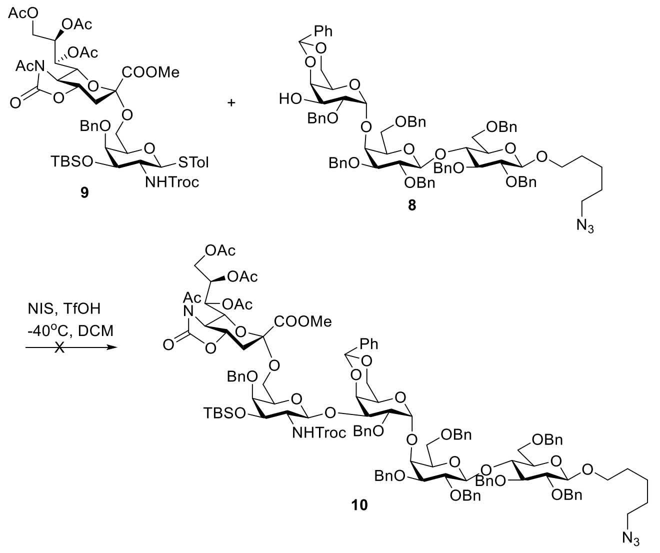

Scheme S2. Synthesis of pentasaccharide **10**

Synthesis of Donor 11

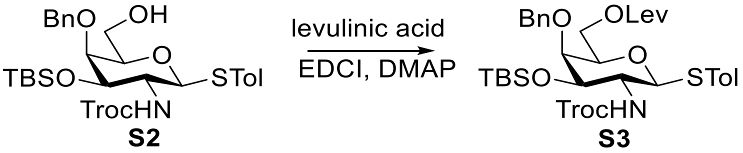

((2*R*,3*S*,4*R*,5*R*,6*S*)-3-(benzyloxy)-4-((*tert*-butyldimethylsilyl)oxy)-6-(*p*-tolylthio)-5-(((2,2,2-trichloroethoxy)carbonyl)amino)tetrahydro-2*H*-pyran-2-yl)methyl 4-oxopentanoate (S3)

A solution of **S2** (0.75 g, 1.1 mmol), pulverized activated 4Å MS (2.0 gmmol^{-1}) in dry DCM (7 mL) was stirred under argon at room temperature for 2 h. The reaction was then cooled to 0 °C followed by addition of EDCI (0.32 g, 1.7 mmol) and DMAP (0.034g, 0.28 mmol). After being stirred overnight at room temperature, the reaction mixture was washed with saturated aq. $NaHCO_3$. After drying over $MgSO_4$, the solution was filtered and concentrated in *vacuo*. The residue was purified by silica gel column chromatography (EtOAc/Hexane 1:2), to give compound **S3** after drying (0.69 g, 80%).^{1}H NMR (600 MHz, $CDCl_3$) δ 7.42 (d, *J* = 8.1 Hz, 2H), 7.36-7.34 (m, 5H), 7.04 (d, *J* = 8.0 Hz, 2H), 5.05-5.01 (m, 3H), 4.75 (d, *J* = 11.9 Hz, 1H), 4.69 (d, *J* = 11.9 Hz, 1H), 4.55 (d, *J* = 11.2 Hz, 1H), 4.30 (dd, J_1 = 11.2 Hz, J_2 = 6.9 Hz, 2H), 4.21 (dd, J_1 = 11.2 Hz, J_2 = 5.7 Hz, 2H), 4.10 (d, *J* = 9.06 Hz, 1H), 3.82-3.73 (m, 3H), 2.75 (t, J_1 = 13.0 Hz, J_2 = 6.4 Hz, 2H), 2.56-2.54 (m, 2H), 2.32 (s, 3H), 2.20 (s, 3H), 0.93 (s, 9H), 0.19 (s, 3H), 0.12 (s, 3H). ^{13}C NMR (150 MHz, $CDCl_3$) δ 206.4, 172.5, 153.6, 138.5, 137.6, 132.5, 129.6, 128.2, 127.7, 127.5, 95.2, 86.1, 75.9, 75.0, 74.8, 73.9, 63.5, 54.1, 37.9, 29.8, 27.9, 25.8, 21.1, 18.0, -3.9, -5.1; HRMS (ESI-TOF, MH^+) calcd for $C_{34}H_{45}Cl_3NO_8SSi$: 762.1668, found: 762.1715.

(2*S*,4a*R*,6*R*,7*R*,8*S*,8a*S*)-6-(((2*R*,3*R*,4*R*,5*R*,6*S*)-3-(benzyloxy)-2-(((4-oxopentanoyl)oxy)methyl)-6-(*p*-tolylthio)-5-(((2,2,2-trichloroethoxy)carbonyl)amino)tetrahydro-2*H*-pyran-4-yl)oxy)-8-((tert-butyldimethylsilyl)oxy)-2-phenylhexahydropyrano[3,2-*d*][1,3]dioxin-7-yl benzoate (11)

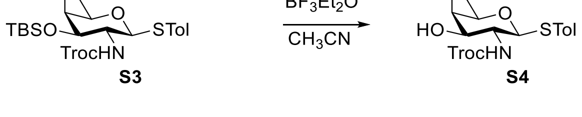

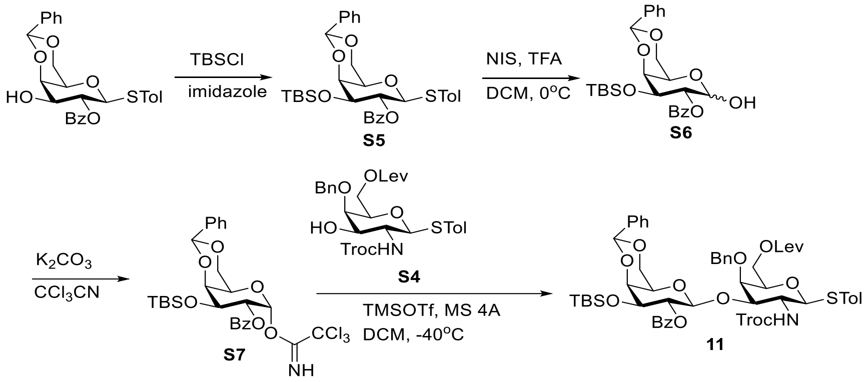

A solution of compound **S3** (0.69 g, 0.9 mmol) in dry CH_3CN (1 mL) was cooled to 0 °C followed by addition BF_3•Et_2O (0.29 mL, 2.7 mmol). After being stirred overnight at room temperature, the reaction mixture was washed with saturated aq. $NaHCO_3$. After drying over $MgSO_4$, the solution was filtered and concentrated in *vacuo*. The residue was purified by silica gel column chromatography (EtOAc/Hexane 1:2), to give compound **S4** after drying (0.53 g, 90%).^{1}H NMR (600 MHz, $CDCl_3$) δ 7.43 (d, J = 8.0 Hz, 2H), 7.38-7.33 (m, 5H), 7.09 (d, J = 8.0 Hz, 2H), 5.17 (d, J = 7.1 Hz, 1H), 4.80-4.71 (m, 5H), 4.37-4.34 (m, 1H), 4.22-4.19 (m, 1H), 3.87 (s, 2H), 3.74-3.70 (m, 2H), 2.77-2.73 (m, 3H), 2.57 (t, J_1 = 13.0 Hz, J_2 = 6.5 Hz, 2H), 2.34 (s, 3H), 2.20 (s, 3H). ^{13}C NMR (150 MHz, $CDCl_3$) δ 206.4, 172.4, 154.9, 138.2, 137.9, 133.0, 129.7, 128.5, 127.8, 95.3, 86.4, 76.2, 75.7, 75.4, 74.7, 73.8, 63.0, 54.3, 37.9, 29.8, 27.8, 21.2; HRMS (ESI-TOF, MH^+) calcd for $C_{28}H_{31}Cl_3NO_8S$: 648.0802, found: 648.0864.

A solution of (2*S*,4a*R*,6*S*,7*R*,8*S*,8a*R*)-8-hydroxy-2-phenyl-6-(*p*-tolylthio) hexahydro pyrano[3,2-*d*][1,3]dioxin-7-yl benzoate [4] (1.0 g, 2.1 mmol), tert-butyldimethylsilyl chloride (0.47 g, 3.1mmol) and imidazole (0.29 g, 4.2 mmol) in dry DMF (10 mL) was stirred under argon at room temperature for overnight. After concentration in *high vacuo*, the reaction mixture was washed with saturated aq. $NaHCO_3$. After drying over $MgSO_4$, the solution was filtered and concentrated *in vacuo*. The residue was purified by silica gel column chromatography (EtOAc/Hexane 1:2), to give compound **S5** after drying (1.11 g, 90%).

To a vigorously stirred solution of **S5** (1.11 g, 1.87 mmol) in DCM (10 mL) and H_2O (1 mL) at 0 °C was added NIS (0.41 g, 1.87 mmol) and TFA (0.143 mL, 1.87 mmol). After the starting material was consumed as indicated by TLC, the reaction was quenched by saturated aq. NaS_2O_3 and $NaHCO_3$. The organic layer was dried over $MgSO_4$ and concentrated *in vacou*. The residue was purified by silica gel column chromatography (EtOAc/Hexane 1:1) to give compound **S6** after drying (0.68 g, 75%).

To a stirred solution of **S6** (0.68 g, 1.40 mmol) and trichloroacetonitrile (0.70 mL, 7.0 mmol) in dry DCM (6 mL) was added K_2CO_3 (0.77 g, 5.60 mmol). After being stirred for 24 h, the reaction mixture was diluted with DCM and filtrated with Celite. The concentrated residue was purified by silica gel column chromatography (EtOAc/Hexane 1:1) to give compound **S7** (0.75 g, 85%) as white foam after drying. ^{1}H NMR (600 MHz, $CDCl_3$) δ 8.53 (s, 1 H), 8.06 (dd, J_1 = 8.6 Hz, J_2 = 1.3 Hz, 2H), 7.62-7.56 (m, 3H), 7.45-7.39 (m, 5H), 6.79 (d, J = 3.4 Hz, 1H), 5.77 (dd, J_1 = 10.1 Hz, J_2 = 3.4 Hz, 1H), 5.64 (s, 1H), 4.58 (dd, J_1 = 10.1 Hz, J_2 = 3.5 Hz, 1H), 4.41 (dd, J_1 = 12.5 Hz, J_2 = 1.1 Hz, 1H), 4.33 (d, J = 3.4, 1H), 4.14 (dd, J_1 = 12.6 Hz, J_2 = 1.38 Hz, 1H), 4.05 (s, 1H), 0.85 (s, 9H), 0.16 (s, 3H), 0.01 (s, 3H). ^{13}C NMR (150 MHz, $CDCl_3$) δ 165.6, 160.5, 137.7, 133.2, 129.8, 129.6, 128.9, 128.3, 128.2, 126.1, 100.8, 95.2, 91.3, 70.2, 69.0, 67.9, 65.6, 25.6, 18.0, -4.5, -4.6.

A solution of **S7** (0.75 g, 1.19 mmol), acceptor **S4** (0.51 g, 0.78 mmol) and pulverized activated 4Å MS (2.0 gmmol^{-1}) in dry DCM (12 mL) was stirred under argon at room temperature for 2 h. The reaction was then cooled to -78°C followed by a slow addition of

TMSOTf (19 µL, 0.12 mmol) via micro-syringe. After being stirred for 1 h, the reaction mixture was neutralized by Et_3N (0.1 mL) and washed with saturated aq. $NaHCO_3$. After drying over $MgSO_4$, the solution was filtered and concentrated in *vacuo*. The residue was purified by silica gel column chromatography (EtOAc/Hexane 1:4) to give compound **11** after drying (0.57 g, 65%). ^1H NMR (600 MHz, $CDCl_3$) δ 8.11 (d, *J* = 7.5 Hz, 2H), 7.62 (t, *J* = 7.4 Hz, 1H), 7.54-7.48 (m, 5H), 7.32-7.28 (m, 7H), 7.22-7.21 (m, 3H), 6.98 (d, *J* = 8.0 Hz, 2H), 5.59-5.56 (m, 2H), 5.11 (d, *J* = 10.3 Hz, 1H), 5.06 (d, *J* = 7.1 Hz, 1H), 4.98 (d, *J* = 11.6 Hz, 1H), 4.77 (d, *J* = 8.0 Hz, 1H), 4.67-4.62 (m, 3H), 4.44-4.41 (m, 2H), 4.21 (dd, J_1 = 11.2 Hz, J_2 = 7.1 Hz, 1H), 4.14-4.12 (m, 2H), 4.03 (d, *J* = 82.3 Hz, 1H), 4.00-3.96 (m, 2H), 3.64 (t, J_1 = J_2 = 6.2 Hz, 1H), 3.53 (s, 1H), 3.46-3.41 (m, 1H), 2.69-2.67 (m, 2H), 2.53-2.42 (m, 2H), 2.30 (s, 3H), 2.16 (s, 3H), 0.76 (s, 9H), 0.06(s, 3H), 0.10 (s, 3H). ^{13}C NMR (150 MHz, $CDCl_3$) δ 206.5, 172.3, 165.0, 153.7, 138.3, 137.7, 137.6, 133.2, 132.3, 130.2, 129.7, 129.6, 129.2, 128.9, 128.8, 128.6, 128.2, 128.0, 127.3, 126.1, 102.0, 101.1, 95.8, 85.2, 77.9, 77.2, 76.8, 76.4, 75.9, 74.4, 74.2, 74.0, 72.3, 72.1, 69.1, 66.8, 63.5, 53.3, 37.9, 29.8, 27.8, 25.4, 21.1, 17.9, -4.6, -4.7; HRMS (ESI-TOF, MH^-) calcd for $C_{54}H_{63}Cl_3NO_{14}SSi$: 1114.2799, found: 1114.2790.

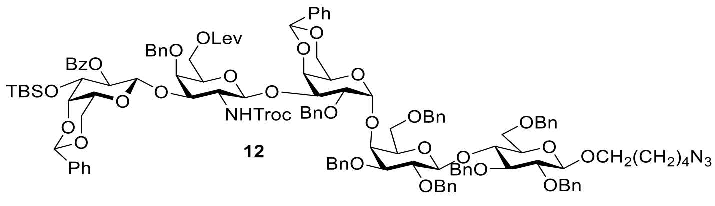

12

2*S*,4a*R*,6*R*,7*R*,8*S*,8a*S*)-6-(((2*S*,3*R*,4*R*,5*R*,6*R*)-2-(((2*S*,4a*R*,6*R*,7*R*,8*S*,8a*S*)-6-(((2*R*,3*S*,4*S*,5*R*,6*S*)-6-(((2*R*,3*R*,4*S*,5*R*,6*R*)-6-((5-azidopentyl)oxy)-4,5-bis(benzyloxy)-2-((benzyloxy)methyl)tetrahydro-2*H*-pyran-3-yl)oxy)-4,5-bis(benzyloxy)-2-((benzyloxy)methyl)tetrahydro-2*H*-pyran-3-yl)oxy)-7-(benzyloxy)-2-phenylhexahydropyrano[3,2-*d*][1,3]dioxin-8-yl)oxy)-5-(benzyloxy)-6-(((4-oxopentanoyl)oxy)methyl)-3-(((2,2,2-trichloroethoxy)carbonyl)amino)tetrahydro-2*H*-pyran-4-yl)oxy)-8-((tert-butyldimethylsilyl)oxy)-2-phenylhexahydropyrano[3,2-*d*][1,3]dioxin-7-yl benzoate (12)

A solution of **11** (0.57 g, 0.51 mmol), acceptor **8** (0.62 g, 0.46 mmol) and pulverized activated 4Å MS (2.0 $gmmol^{-1}$) in dry DCM (12 mL) was stirred under argon at room temperature for 2 h. The mixture was then cooled to -40 °C followed by addition of NIS (115 mg, 0.51 mmol) and TfOH (278 µL, 0.14 mmol, 0.5 M in ether). The reaction was stirred at -40°C for 2 h, then neutralized by saturated $NaHCO_3$ (aq.), diluted with DCM and filtered with a pad of Celite. The filtrate was poured into a mixture of saturated $NaHCO_3$ (aq.) and saturated $Na_2S_2O_3$ (aq.). The aqueous layer was extracted with two portions of DCM. The combined extracts were washed with brine, dried over Mg_2SO_4, filtered, and concentrated in *vacuo*. The residue was purified by silica gel column chromatography (EtOAc/Hexane 1:4) to give compound **12** after drying (0.64 g, 60 %). ^1H NMR (600 MHz, $CDCl_3$) δ 8.11 (d, *J* = 7.5 Hz, 2H), 7.56-7.50 (m, 3H), 7.42-7.40 (m, 7H), 7.36-7.29 (m, 23H), 7.28-7.23 (m, 15H), 7.18 (m, 3H), 7.12 (d, *J* = 7.0 Hz, 2H), 5.59-5.57 (m, 2H), 5.36 (s, 1H), 5.11 (d, *J* = 2.8 Hz, 2H), 5.03-4.96 (m, 2H), 4.91-4.85 (m, 4H), 4.82-4.66 (m, 7H), 4.59-4.46 (m, 5H), 4.42-4.34 (m, 3H), 4.26-4.00 (m, 13H), 3.96-3.89 (m, 4H), 3.81 (m, 2H), 3.74 (d, *J* = 10.0 Hz, 1H), 3.65-3.60 (m, 3H), 3.54-3.49 (m, 4H), 3.45-3.40 (m, 3H), 3.31-3.27 (m, 3H), 3.22 (t, J_1 = 13.8 Hz, J_2 = 6.9 Hz, 3H), 2.57-2.54 (m, 2H), 2.45-2.34 (m, 2H), 2.08 (s, 3H), 1.70-1.64 (m, 2H), 1.64-1.59 (m, 2H), 1.52-1.43 (m, 2H), 0.77 (s, 9H), 0.06 (s, 3H), -0.11 (s, 3H). ^{13}C NMR (150 MHz, $CDCl_3$) δ 206.2, 172.1, 165.0, 153.5, 139.3, 138.8, 138.6, 138.5, 138.4, 138.2, 138.1, 138.0, 137.8, 132.9, 130.2, 129.8, 129.1, 128.8, 128.7, 128.5, 128.4, 128.4, 128.2, 128.2, 128.0, 127.9, 127.9, 127.8, 127.6, 127.6, 127.5, 127.5, 127.4, 127.4, 127.3, 127.0, 126.3, 126.2, 103.5, 103.3, 101.5, 101.0, 100.7, 100.6, 100.5, 100.5, 96.1, 82.0, 81.9, 81.3, 79.0, 76.5, 76.0, 75.2, 75.0, 74.9, 74.7, 74.4, 74.3, 73.8, 73.4, 73.4, 73.0, 72.9, 72.2, 72.0, 71.3, 69.5, 69.1, 69.1, 68.5, 67.0, 66.8, 63.0, 62.9, 55.0, 51.3, 37.7, 29.7, 29.3, 28.6, 27.7, 25.4, 23.4,

17.9, -4.6, -4.8; HRMS (ESI-TOF, MNa+) calcd for $C_{126}H_{143}Cl_3N_4O_{30}SiNa$: 2349.8493, found: 2349.8374.

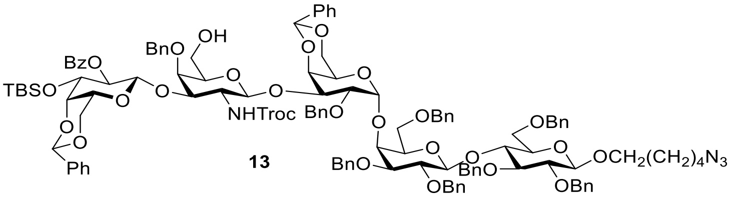

13

(2*S*,4a*R*,6*R*,7*R*,8*S*,8a*S*)-6-(((2*S*,3*R*,4*R*,5*R*,6*R*)-2-(((2*S*,4a*R*,6*R*,7*R*,8*S*,8a*S*)-6-(((2*R*,3*S*,4*S*,5*R*,6*S*)-6-(((2*R*,3*R*,4*S*,5*R*,6*R*)-6-((5-azidopentyl)oxy)-4,5-bis(benzyloxy)-2-((benzyloxy)methyl)tetrahydro-2*H*-pyran-3-yl)oxy)-4,5-bis(benzyloxy)-2-((benzyloxy)methyl)tetrahydro-2*H*-pyran-3-yl)oxy)-7-(benzyloxy)-2-phenylhexahydropyrano[3,2-*d*][1,3]dioxin-8-yl)oxy)-5-(benzyloxy)-6-(hydroxymethyl)-3-(((2,2,2-trichloroethoxy)carbonyl)amino)tetrahydro-2*H*-pyran-4-yl)oxy)-8-((tert-butyldimethylsilyl)oxy)-2-phenylhexahydropyrano[3,2-*d*][1,3]dioxin-7-yl benzoate (13)

The pentasaccharide precursor **12** (0.64 g, 0.27 mmol) was dissolved in dry pyridine (3 ml), and 1 mM hydrazine hydrate (NH_2NH_2•H_2O) in pyr/AcOH mixture (vol/vol = 3:2) (0.81 ml, 0.81 mmol) was added. The reaction mixture was stirred at 0 °C for 4 h and then penta-2, 4-dione (1 ml) was added. The solvent was removed under reduced pressure, and the residue was dissolved in CH_2Cl_2, washed with H_2O and brine, dried (Na_2SO_4), concentrated, and purified by flash column chromatography purification (hexane/EtOAc, 2:1) to provide the desired pentasaccharide **13** (0.52 g, 85%) as a white glassy residue. ^{1}H NMR (600 MHz, $CDCl_3$) δ 8.11 (d, *J* = 7.4 Hz, 2H), 7.56-7.52 (m, 3H), 7.43-7.39 (m, 8H), 7.36-7.28 (m, 27H), 7.27-7.22 (m, 13H), 7.13 (d, *J* = 6.8 Hz, 2H), 5.61 (s, 1H), 5.58 (t, J_1 = 8.8 Hz, J_2 = 8.8 Hz, 1H), 5.33 (s, 1H), 5.10 (d, *J* = 3.4 Hz, 1H), 5.01-4.97 (m, 2H), 4.92-4.83 (m, 5H), 4.78-4.66 (m, 6H), 4.60-4.49 (m, 5H), 4.26 (m, 1H), 4.18-3.95 (m, 13H), 3.85-3.75 (m, 3H), 3.66 (t, J_1 = 8.9 Hz, J_2 = 8.9 Hz, 1H), 3.61-3.41 (m, 11H), 3.32-3.26 (m, 3H), 3.22 (t, J_1 = 6.8 Hz, J_2 = 7.0Hz, 3H), 1.70-1.60 (m, 4H), 1.52-1.44 (m, 2H), 0.77 (s, 9H), 0.06 (s, 3H), -0.11 (s, 3H). ^{13}C NMR (150 MHz, $CDCl_3$) δ 165.0, 153.5, 139.4, 138.8, 138.5, 138.4, 138.2, 138.1, 138.0, 137.8, 132.9, 130.3, 129.8, 129.5, 128.8, 128.6, 128.5, 128.4, 128.3, 128.3, 128.1, 127.9, 127.8, 127.7, 127.6, 127.6, 127.6, 127.5, 127.5, 127.3, 127.1, 126.4, 126.2, 103.5, 103.3, 101.7, 101.1, 100.6, 100.4, 100.3, 96.0, 82.3, 81.8, 81.2, 78.8, 75.8, 75.1, 74.9, 74.7, 74.4, 74.2, 74.1, 73.9, 73.8, 73.5, 73.3, 73.1, 73.0, 72.9, 72.3, 72.1, 72.0, 69.5. 69.1, 69.0, 68.5, 67.1, 66.8, 63.1, 62.1, 55.2, 51.3, 29.7, 29.3, 28.6, 25.4, 23.4, 17.9, -4.6, -4.8; HRMS (ESI-TOF, MNa+) calcd for $C_{121}H_{137}Cl_3N_4O_{28}SiNa$: 2251.8125, found: 2250.8040.

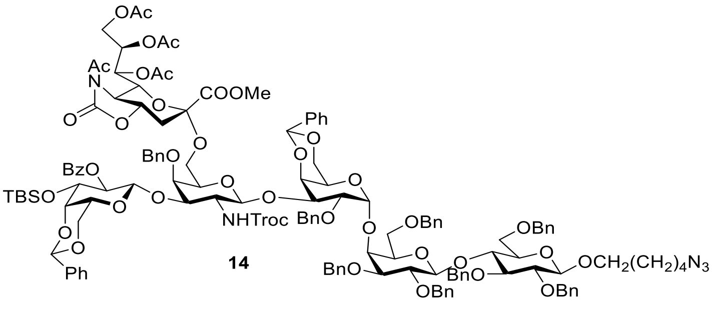

14

(2*R*)-1-((3a*R*,4*R*,6*R*,7a*S*)-3-acetyl-6-(((2*R*,3*R*,4*R*,5*R*,6*S*)-6-(((2*S*,4a*R*,6*R*,7*R*,8*S*,8a*S*)-6-(((2*R*,3*S*,4*S*,5*R*,6*S*)-6-(((2*R*,3*R*,4*S*,5*R*,6*R*)-6-((5-azidopentyl)oxy)-4,5-bis(benzyloxy)-2-((benzyloxy)methyl)tetrahydro-2*H*-pyran-3-yl)oxy)-4,5-bis(benzyloxy)-2-((benzyloxy)methyl)tetrahydro-2*H*-pyran-3-yl)oxy)-7-(benzyloxy)-2-phenylhexahydropyrano[3,2-*d*][1,3]dioxin-8-yl)oxy)-4-(((2*S*,4a*R*,6*R*,7*R*,8*S*,8a*S*)-7-(benzoyloxy)-8-((tert-butyldimethylsilyl)oxy)-2-phenylhexahydropyrano[3,2-*d*][1,3]dioxin-6-yl)oxy)-3-(benzyloxy)-5-(((2,2,2-

trichloroethoxy)carbonyl)amino)tetrahydro-2*H*-pyran-2-yl)methoxy)-6-(methoxycarbonyl)-2-oxohexahydro-4*H*-pyrano[3,4-d]oxazol-4-yl)propane-1,2,3-triyl triacetate (14)

A solution of **13** (0.038 g, 0.017 mmol), phosphate donor **15** (0.057 g, 0.085 mmol) and pulverized activated 4Å MS (2.0 $gmmol^{-1}$) in dry DCM (1 mL) was stirred under argon at room temperature for 2 h. The mixture was then cooled to -70 ^oC followed by addition of TMSOTf (5 µL, 0.027 mmol). The reaction was stirred at -70 ^oC for 16 h, then neutralized by saturated $NaHCO_3$ (aq.), diluted with DCM and filtered with a pad of Celite. The aqueous layer was extracted with two portions of DCM. The combined extracts were washed with brine, dried over Mg_2SO_4, filtered, and concentrated in *vacuo*. The residue was purified by silica gel column chromatography (EtOAc/DCM/Toluene 1:4:4) to give compound **14** after drying (0.025 g, 55 %). ^1H NMR (600 MHz, $CDCl_3$) δ 8.05 (d, *J* = 7.4 Hz, 2H), 7.56-7.55 (m, 2H), 7.50-7.47 (m, 1H), 7.44-7.37 (m, 6H), 7.36-7.29 (m, 19H), 7.27-7.26 (m, 6H), 7.24-7.18 (m, 17H),7.09 (d, *J* = 7.2 Hz, 2H), 5.63 (d, *J* = 8.6 Hz, 1H), 5.58-5.55 (m, 2H), 5.47 (s, 1H), 5.43-5.41 (m, 1H), 5.18 (m, 1H), 5.11(d, *J* = 3.2 Hz, 1H), 4.98-4.86 (m, 5H), 4.81-4.77 (m, 5H), 4.69 (d, *J* = 11.1 Hz, 1H), 4.58-4.53 (m, 3H), 4.49-4.41 (m, 5H), 4.38-4.33 (m, 3H), 4.28 (d, *J* = 8.6 Hz, 1H), 4.16-3.78 (m, 20H), 3.74-3.69 (m, 4H), 3.65-3.60 (m, 3H), 3.56-3.48 (m, 3H), 3.45-3.39 (m, 2H), 3.33-3.28 (m, 5H), 3.25-3.20 (m, 5H), 2.75 (dd, J_1 = 12.0 Hz, J_2 = 3.1Hz, 1H), 2.49 (s, 3H), 2.09-2.01 (m, 7H), 1.98 (s, 3H), 1.70-1.59 (m, 4H), 1.51-1.43 (m, 2H), 0.77 (s, 9H), 0.05 (s, 3H), -0.12 (s, 3H). ^{13}C NMR (150 MHz, $CDCl_3$) δ 171.7, 170.9, 170.1, 170.0, 167.9, 165.1, 153.8, 153.6, 139.3, 139.2, 139.0, 138.8, 138.5, 138.4, 138.1, 138.1, 137.7, 133.0, 130.2, 129.7, 128.9, 128.5, 128.4, 128.4, 128.3, 128.2, 128.2, 127.9, 127.9, 127.8, 127.6, 127.6, 127.6, 127.5, 127.4, 127.3, 127.1, 126.8, 126.2, 126.1, 103.5, 103.4, 101.3, 100.9, 100.8, 100.3, 100.3, 99.3, 96.2, 82.0, 81.7, 81.3, 79.0, 78.7, 77.7, 75.5, 76.4, 75.2, 75.1, 75.0, 74.9, 74.8, 74.6, 74.5, 74.2, 74.0, 73.7, 73.6, 73.4, 73.1, 73.0, 72.9, 72.2, 72.2, 72.0, 71.9, 71.3, 69.5, 69.1, 69.0, 68.6, 68.5, 66.9, 66.9, 63.9, 63.1, 59.1, 54.9, 52.8, 51.3, 36.0, 29.7, 29.3, 28.6, 24.7, 23.4, 21.0, 20.9, 17.9, -4.6, -4.8; HRMS (ESI-TOF, MNa^+) calcd for $C_{121}H_{137}Cl_3N_4O_{28}SiNa$: 2687.9556, found: 2687.9475.

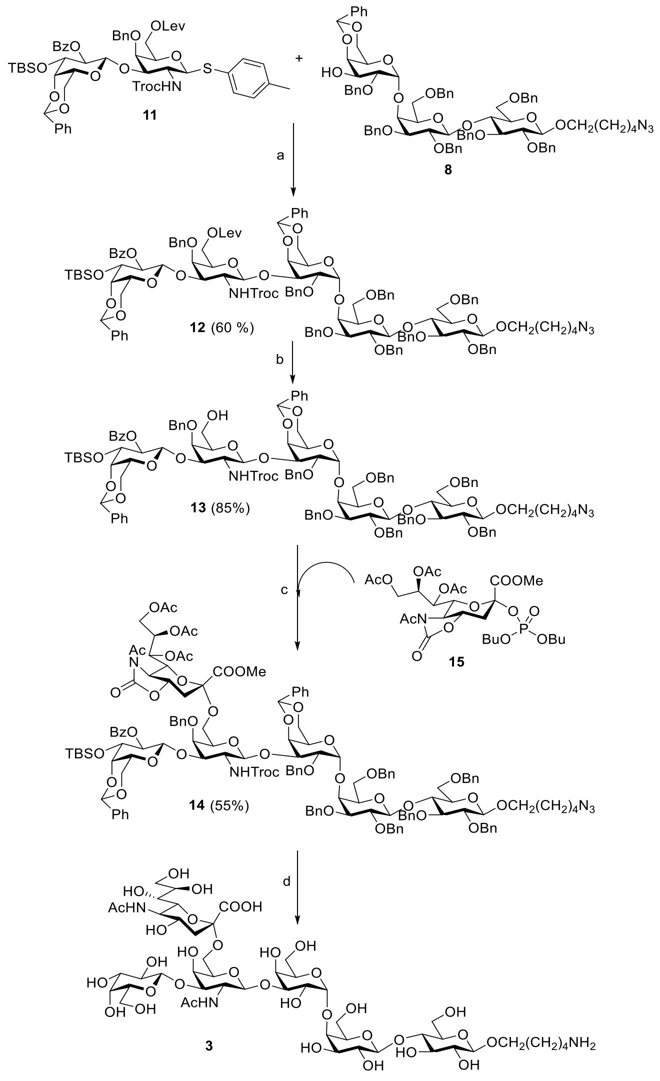

Scheme S3. Synthesis of hexasaccharide 3[a]

[a]Reagents and conditions: **(a)** NIS, TfOH, MS 4 Å, CH_2Cl_2, -40°C, 2 h, 60%. **(b)** NH_2NH_2, 0°C, 2h, 85%. **(c)** TMSOTf, MS 4 Å, CH_2Cl_2, -70°C, 24 h, 55%. **(d)** (i) LiOH, dioxane, H_2O, 100°C, 48 h; (ii) TBAF, CH_2Cl_2, overnight; (iii) Py, Ac_2O, overnight; (iv) NaOMe, CH_2Cl_2, MeOH, overnight; (v) $Pd(OH)_2$, H_2, MeOH, AcOH, H_2O, r.t., 8 h.

General procedures for the deprotection of oligosaccharide. LiOH (5.0 mmole, 50.0 eq) was added to a stirred solution of protected oligosaccharide (0.1 mmole, 1.00 eq) in 1,4-dioxane (5.00 mL) and H_2O (5.00 mL) at room temperature. After stirring at 80°C for 36 h, the reaction mixture was constranted *in vacuo*. The residue was purified by reverse–phase column chromatography (Bond Elut-C18) to give the product residue. $NaHCO_3$ (5.0 mmole, 50.0 eq) and acetic anhydride (5.0 mmole, 50.0 eq) were added to a stirred solution of the above residue in H_2O (3.00 mL) at room temperature. After stirring at the same temperature for 1 h, $NaHCO_3$ (5.0 mmole, 50.0 eq) and acetic anhydride (5.0 mmole, 50.0 eq) were added to the reaction mixture at room temperature followed by stirring at the same temperature for 1 h, and LiOH (5.0 mmole, 50.0 eq) was added to the reaction mixture. After stirring at the same temperature for 12 h, the reaction mixture was evaporated *in vacuo*. The residue was purified by reverse–phase column chromatography (Bond Elut-C18). $Pd(OH)_2$ (1 mmole) was added to a stirred solution of the above residue in methanol (5.00 mL) and HOAc (0.50 mL). The reaction mixture was hydrogenolyzed for 12 h under H_2 gas atmosphere, then filtered, and the filtrate was evaporated *in vacuo*. The residue was purified

by reverse–phase column chromatography (Bond Elut-C18) to give deprotected oligosaccharide after drying.

SA-Gb5-NH_2 (3)

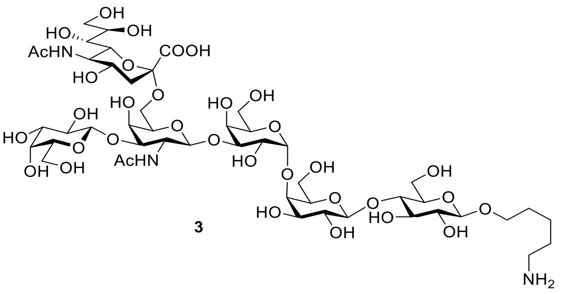

3

^{1}H NMR (600 MHz, $CDCl_3$) δ 4.85 (d, *J*= 4.0 Hz, 1H), 4.61 (d, *J*= 8.5 Hz, 1H), 4.43 (dd, J_1= 9.6 Hz, J_2= 7.8 Hz, 2H), 4.37 (d, *J*= 7.7 Hz, 1H), 4.32 (t, J_1= J_2= 6.6 Hz, 1H), 4.20 (d, *J*= 3.0 Hz, 1H), 4.13 (d, *J*= 3.3 Hz, 1H), 3.99-3.50 (m, 39H), 3.45 (dd, J_1= 9.8 Hz, J_2= 7.8 Hz, 1H), 3.23 (t, J_1= 8.3 Hz, J_2= 8.8 Hz, 1H), 2.94 (t, J_1= J_2= 7.8 Hz, 2H), 2.66 (dd, J_1= 12.3 Hz, J_2= 4.8 Hz, 2H), 1.96 (s, 3H), 1.96 (s, 3H), 1.65-1.57 (m, 6H), 1.42-1.38 (m, 2H). ^{13}C NMR (150 MHz, $CDCl_3$) δ 175.1, 175.0, 173.4, 104.8, 103.3, 102.8, 101.9, 100.4, 79.7, 78.9, 78.8, 77.1, 75.5, 75.0, 74.8, 74.5, 72.9, 72.6, 72.5, 72.1, 71.7, 70.8, 70.6, 70.4, 70.0, 68.9, 68.6, 68.3, 68.2, 67.9, 67.7, 63.4, 62.6, 61.0, 60.6, 60.3, 60.1, 51.9, 51.4, 40.2, 39.3, 28.1, 26.4, 22.3, 22.1, 22.0. HRMS (ESI-TOF, MH^-) calcd for C48H82N3O34: 1244.4774, found: 1244.4818.

Protocol of enzymatic reaction. An oligosaccharide (10 μmol/mL), Neu5Ac (15 μmol/mL) and CTP (40 μmol/mL) were dissolved in Tris-HCl buffer (150 mM, pH 8.5, 20 mM $MgCl_2$). After addition of NmCSS [5] (0.2 mg/ mL) and enzyme (Psp2,6ST(15–501)-His_6 [6] or His_6-Pd2,6ST(16-497) [7-10]/ 1mg/ mL), the reaction was keep at 27 ^oC for 2-7 h with agitation (700 rpm). The reaction was monitored by TLC (n-butanol: AcOH: water = 4:2:1). The reaction was stopped by using Amicon Ultra 10K to filter the enzymes, and the filtrate was concentrated and purified by C18 column (0-15% MeOH) and G10 column (20 mM NH_4HCO_3).

Compound 2a

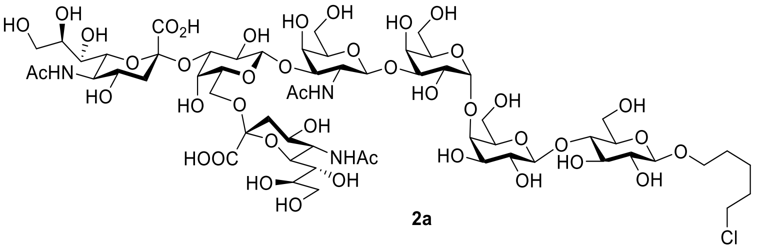

2a

^{1}H NMR (600 MHz, D_2O) δ 4.87 (d, *J* = 3.9 Hz, 1H), 4.64 (d, *J* = 4.5 Hz, 1H), 4.48-4.45 (m, 3H), 4.35 (dd, *J* = 6.45, 6.45 Hz, 1H), 4.22 (d, *J* = 2.5 Hz, 1H), 4.15 (d, *J* = 2.7 Hz, 1H), 4.04-3.99 (m, 3H), 3.96-3.93 (m, 2H), 3.39-3.73 (m, 17H), 3.72-3.52 (m, 21H), 3.48 (dd, *J* = 8.8, 8.8 Hz, 1H), 3.27-3.24 (m, 1H), 2.71-2.66 (m, 2H), 1.99-1.98 (m, 9H), 1.79-1.72 (m, 3H), 1.64-1.59 (m, 3H), 1.49-1.44 (m, 2H). ^{13}C NMR (150 MHz, D_2O) δ 175.0, 174.9, 173.8, 173.3, 160.4, 104.3, 103.3, 103.0, 101.9, 100.36, 100.3, 99.8, 79.9, 78.7, 77.0, 75.4, 74.7, 74.6, 74.5, 72.9, 72.7, 72.5, 72.0, 71.7, 71.7, 70.8, 70.3, 70.2, 68.9, 68.3, 68.2, 68.0, 67.7, 67.6, 67.4, 63.3, 62.6, 62.5, 61.1, 60.3, 60.2, 60.0, 51.8, 51.6, 51.2, 45.5, 40.1, 39.5, 31.5, 28.0, 22.5, 22.3, 22.0. HRMS (ESI-TOF, MH^+) calcd for C59H98ClN3O42: 778.7729, found: 778.7673.

Compound 1a

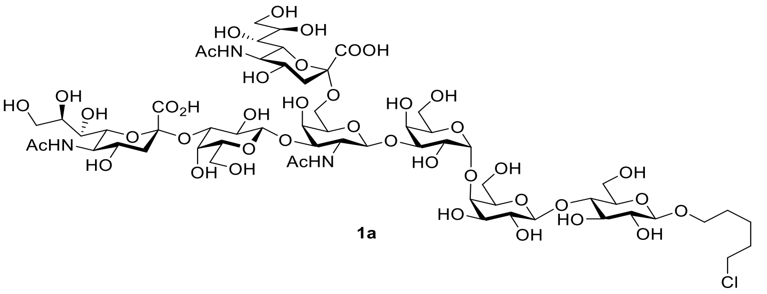

1a

^{1}H NMR (600 MHz, D_2O) δ 4.93 (d, *J* = 3.9 Hz, 1H), 4.68 (d, *J* = 8.5 Hz, 1H), 4.53-4.50 (m, 3H), 4.41 (dd, *J* = 6.4, 6.4 Hz, 1H), 4.28 (d, *J* = 2.9 Hz, 1H), 4.20 (d, *J* = 3.1 Hz, 1H), 4.09-4.07 (m, 3H), 4.02-3.53 (m, 42H), 3.32-3.29 (m,1H), 2.77-2.72 (m, 2H), 2.04-2.03 (m, 9H), 1.84-1.77 (m, 3H), 1.70-1.64 (m, 3H), 1.54-1.69 (m, 2H). ^{13}C NMR (150 MHz, D_2O) δ 175.0, 175.0, 194.9, 173.9, 173.4, 104.6, 103.3, 102.8, 101.9, 100.4, 100.3, 99.7, 79.8, 79.0, 78.8, 77.0, 75.6, 75.5, 74.8, 74.7, 74.5, 72.9, 72.9, 72.8, 72.6, 72.1, 71.8, 71.7, 70.8, 70.4, 70.3, 69.0, 68.9, 68.9, 68.4, 68.2, 68.0, 67.7, 67.6, 67.4, 63.4, 62.6, 62.5, 60.9, 60.6, 60.3, 60.1, 51.9, 51.6, 51.2, 45.5, 40.2, 39.7, 31.5, 28.0, 22.5, 22.3, 22.0. HRMS (ESI-TOF, MH^{+}) calcd for C59H98ClN3O42: 778.7729, found: 778.7616.

Compound 4a

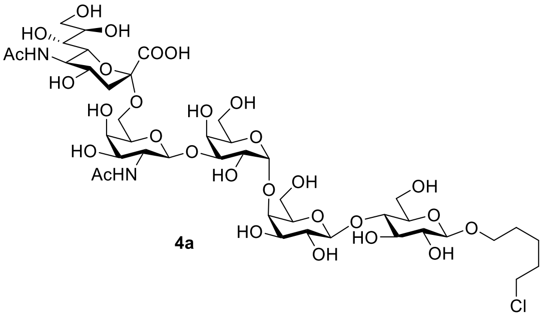

4a

^{1}H NMR (600 MHz, D_2O) δ 4.95 (d, *J* = 4.0 Hz, 1H), 4.65 (d, *J* = 8.5 Hz, 1H), 4.54 (d, *J* = 7.7 Hz, 1H), 4.52 (d, *J* = 8.0 Hz, 1H), 4.42 (dd, *J* = 6.4, 6.4 Hz, 1H), 4.30 (d, *J* = 2.5 Hz, 1H), 4.07 (d, *J* = 2.9 Hz, 1H), 4.03-3.81 (m, 14H), 3.78-3.60 (m, 17H), 3.34-3.32 (m, 1H), 2.77 (dd, *J* = 12.3, 4.5 Hz, 1H), 2.07-2.06 (m, 6H), 1.86-1.82 (m, 2H), 1.72-1.70 (m, 3H), 1.57-1.51 (m, 2H). ^{13}C NMR (150 MHz, D_2O) δ 175.1, 175.0, 173.4, 103.3, 103.3, 103.1, 103.0, 102.0, 100.4, 100.3, 79.0, 78.9, 77.2, 77.0, 75.5, 75.4, 74.8, 74.5, 73.2, 73.2, 73.0, 72.9, 72.6, 72.2, 71.8, 71.6, 70.8, 70.7, 70.7, 70.4, 70.4, 70.3, 70.2, 69.0, 68.8, 68.2, 67.8, 67.7, 67.6, 67.6, 63.6, 63.4, 62.7, 62.6, 60.7, 60.6, 60.3, 60.2, 60.1, 52.6, 52.4, 51.9, 51.8, 45.6, 40.2, 31.6, 28.0, 22.6, 22.4, 22.3, 22.2, 22.1, 22.0. HRMS (ESI-TOF, MH^{+}) calcd for C42H71ClN2O29: 552.1988, found: 552.1938.

Compound 5a

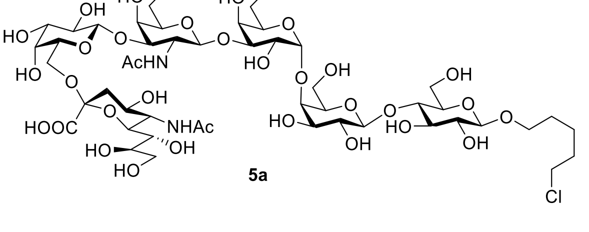

5a

^{1}H NMR (600 MHz, D_2O) δ 4.90 (d, *J* = 4.0 Hz, 1H), 4.67 (d, *J* = 8.5 Hz, 1H), 4.50-4.47 (m, 2H), 4.42 (d, *J* = 7.8 Hz, 1H), 4.38 (dd, *J* = 6.9, 6.9 Hz, 1H), 4.25 (d, *J* = 3.1 Hz, 1H), 4.17 (d, *J* = 3.2 Hz, 1H), 4.07-4.02 (m, 2H), 3.99-3.95 (m, 2H), 3.94-3.76 (m, 14H), 3.74-3.56 (m, 18H), 4.17 (dd, *J* = 3.9 Hz, 1H), 3.49 (dd, *J* = 9.8, 7.8 Hz, 1H), 3.28 (m, 1H), 2.72 (dd, *J* = 12.6, 4.6 Hz, 1H), 2.02-2.01 (m, 6H), 1.82-1.77 (m, 2H), 1.67-1.62 (m, 3H), 1.52-1.47 (m, 2H). ^{13}C NMR (150 MHz, D_2O) δ 175.1, 175.0, 173.4, 104.6, 103.3, 103.0, 101.9, 100.4, 100.3, 79.7, 78.7, 77.1, 75.4, 74.7, 74.7, 74.5, 73.1, 72.9, 72.6, 72.3, 72.1, 71.7, 70.8, 70.4, 70.3, 70.2, 68.9, 68.4, 68.2, 67.9, 67.6, 63.4, 62.6, 61.1, 60.3, 60.2, 60.0, 51.8, 51.4, 45.5, 40.1, 31.5, 28.0, 22.5, 22.2, 22.0. HRMS (ESI-TOF, MH^+) calcd for C48H81ClN2O34: 633.2252, found: 633.2184.

Compound 16

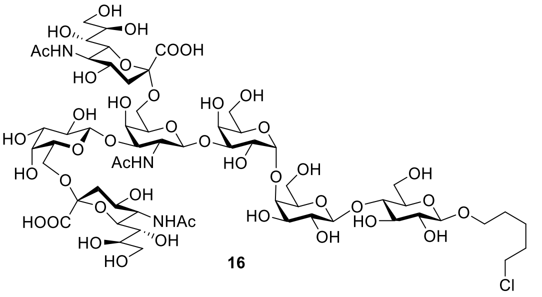

^{1}H NMR (600 MHz, D_2O) δ 4.95 (d, *J* = 3.9 Hz, 1H), 4.68 (d, *J* = 8.5 Hz, 1H), 4.55-4.52 (m, 2H), 4.48 (d, *J* = 7.7 Hz, 1H), 4.44 (dd, *J* = 6.4, 6.4 Hz, 1H), 4.32 (d, *J* = 2.8 Hz, 1H), 4.17 (d, *J* = 3.1 Hz, 1H), 4.12-4.07 (m, 2H), 4.04-4.01 (m, 3H). 3.97-3.81 (m, 18H), 3.79-3.59 (m, 32H), 3.53, (d, *J* = 9.9, 7.9 Hz, 1H), 3.33 (m, 1H), 2.78-2.75 (m, 2H), 2.07-2.05 (m, 9H), 1.86-1.82 (m, 2H), 1.74-1.67 (m, 4H), 1.56-1.51 (m, 2H). ^{13}C NMR (150 MHz, D_2O) δ 175.1, 175.0, 173.5, 173.4, 104.5, 103.4, 102.8, 102.0, 100.6, 100.4, 100.3, 79.1, 78.9, 78.9, 77.0, 75.5, 74.8, 74.5, 73.1, 73.1, 73.0, 72.7, 72.5, 72.3, 72.2, 71.8, 71.6, 70.8, 70.5, 70.4, 70.3, 68.9, 68.4, 68.3, 68.2, 68.1, 68.0, 67.6, 63.2, 62.7, 61.4, 60.7, 60.3, 60.1, 59.4, 52.0, 51.9, 51.4, 45.5, 40.2, 40.2, 31.5, 28.0, 22.6, 22.3, 22.0. HRMS (ESI-TOF, MH^+) calcd for C59H98ClN3O42: 778.7729, found: 778.7612.

Compound 17

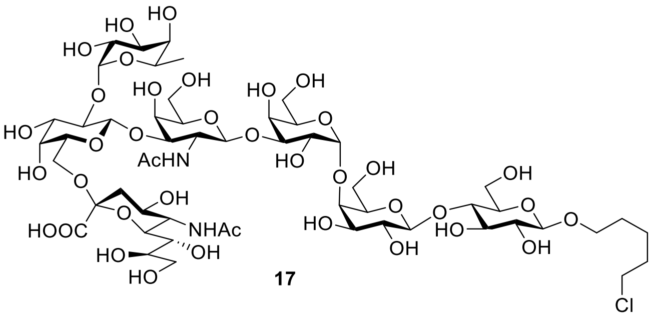

^{1}H NMR (600 MHz, D_2O) δ 5.22 (d, *J* = 3.8 Hz, 1H), 4.89 (d, *J* = 3.7 Hz, 1H), 4.60 (d, *J* = 7.7 Hz, 1H), 4.54 (d, *J* = 7.3 Hz, 1H), 4.52-4.49 (m, 2H), 4.40 (dd, *J* = 6.4, 6.4 Hz, 1H), 4.25-2.22 (m, 2H), 4.09 (s, 1H), 4.03 (d, *J* = 2.6 Hz, 1H), 4.00-3.87 (m, 10H), 3.86-3.76 (m, 9H), 3.75-3.67 (m, 10H), 3.65-3.58 (m, 10H), 3.31-3.28 (m, 1H), 2.73 (dd, *J* = 12.4, 4.5 Hz, 1H), 2.04-2.04 (m, 6H), 1.83-1.78 (m, 2H), 1.70-1.64 (m, 3H), 1.53-1.48 (m, 2H), 1.21 (d, *J* = 6.5 Hz, 1H). ^{13}C NMR (150 MHz, D_2O) δ 177.8, 177.0, 176.1, 106.7, 106.1, 104.7, 104.7, 103.1, 103.1, 102.0, 81.5, 81.0, 79.7, 79.2, 78.7, 78.2, 77.5, 77.2, 76.2, 75.8, 75.7, 75.4, 74.8, 74.6, 74.5, 73.5, 73.1, 72.8, 72.2, 71.9, 71.6, 71.2, 70.9, 70.9, 70.8, 70.6, 69.5, 65.9, 65.4, 63.8, 63.1, 63.0, 62.8, 54.6, 54.3, 48.2, 42.8, 34.2, 30.7, 25.3, 25.0, 24.7, 18.0. HRMS (ESI-TOF, MH^+) calcd for C54H91ClN2O38: 706.2542, found: 706.2562.

Compound 18

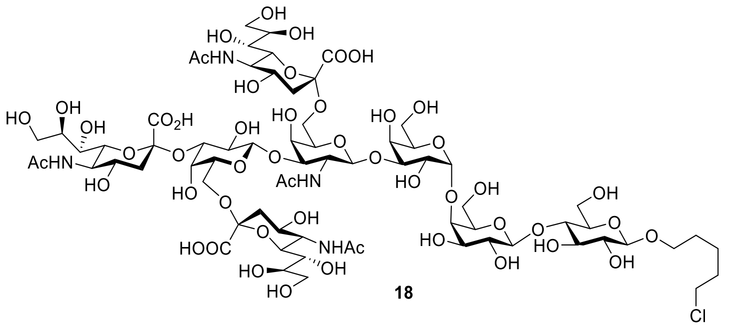

18

^{1}H NMR (600 MHz, D2O) δ 4.96 (d, *J* = 4.0 Hz, 1H), 4.69 (d, *J* = 8.5 Hz, 1H), 4.56-4.54 (m, 3H), 4.45 (dd, *J* = 6.5, 6.5 Hz, 1H), 4.33 (d, *J* = 2.9 Hz, 1H), 4.17 (d, *J* = 3.0 Hz, 1H), 4.12-4.08 (m, 3H), 4.05-4.02 (m,3H), 3.99-3.80 (m, 23H), 3.79-3.71 (m, 23H), 3.70-3.59 (m,16H),3.56 (dd, *J* = 9.7, 8.0 Hz, 1H), 3.33 (dd, *J* = 8.5, 8.5 Hz, 1H), 2.80-2.75 (m, 3H), 2.07-2.06 (m, 12H), 1.87-1.80 (m, 3H), 1.74-1.68 (m, 4H), 1.57-1.53 (m, 2H). ^{13}C NMR (150 MHz, D2O) δ 175.0, 175.0, 174.9, 173.8, 173.5, 173.4, 104.2, 103.4, 102.8, 102.0, 100.6, 100.4, 100.3, 99.8, 79.1, 79.0, 79.0, 76.9, 75.5, 75.4, 74.8, 74.5, 73.1, 73.0, 72.8, 72.7, 72.5, 72.2, 71.8, 71.7, 71.5, 70.8, 70.4, 70.4, 68.9, 68.9, 68.5, 68.3, 68.2, 68.1, 67.9, 67.6, 67.3, 63.9, 63.2, 62.7, 62.7, 62.5, 61.3, 60.7, 60.3, 60.1, 59.4, 52.0, 51.9, 51.6, 51.3, 45.5, 40.2, 40.2, 39.6, 31.6, 28.0, 22.6, 22.4, 22.0. HRMS (ESI-TOF, MH^+) calcd for C54H91ClN2O38: 1847.6346, found: 1847.6340.

Procedures for the synthesis of oligosaccharides with C5-NH2 tail at the reducing end.

NaN_3 (5.0 mmole, 50.0 eq) was added to a stirring solution of oligosaccharide with C5-Cl-linker (0.1 mmole, 1.00 eq) in DMF (5.00 mL) at room temperature. After stirring at 50°C for 48 h, the reaction mixture was concentrated in vacuo and the residue was purified by Sephadex G10 column to give the product residue. Pd $(OH)_2$ (1 mmole) was added to a stirred solution of the above residue in methanol (5.00 mL) and HOAc (0.50 mL). The reaction mixture was hydrogenolyzed for 12 h under H_2 gas atmosphere, then filtered, and the filtrate was evaporated in vacuo. The residue was purified by reverse–phase column chromatography (Bond Elut-C18) to give oligosaccharide with NH_2-linker.

Compound 1

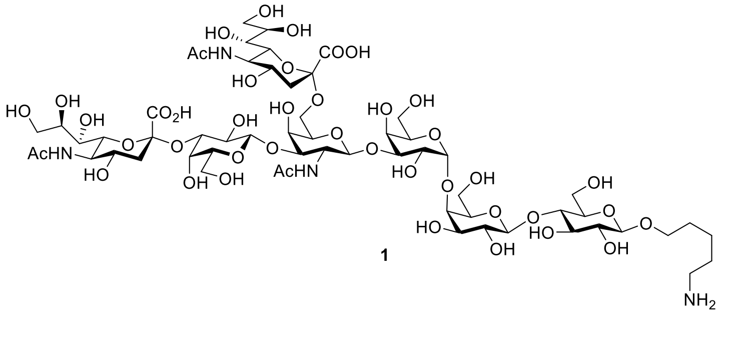

1

^{1}H NMR (600 MHz, D2O) δ 4.85 (d, *J*= 3.8 Hz, 1H), 4.61 (d, *J*= 8.5 Hz, 1H), 4.45-4.42 (m, 3H), 4.32 (t, *J*= 6.6 Hz, 1H), 4.20 (d, *J*= 2.8 Hz, 1H), 4.12 (d, *J*= 3.3 Hz, 1H), 4.00-3.97 (m, 3H), 3.94-3.89 (m, 3H), 3.88-3.74 (m, 14H), 3.73-3.65 (m, 6H), 3.64-3.54 (m, 15H), 3.53-3.46 (m, 3H), 3.23 (t, *J*= 8.1, 8.8 Hz, 1H), 2.94 (t, *J*= 7.5 Hz, 2H), 2.70-2.64 (m, 2H), 1.96-1.95 (m, 9H), 1.72 (t, *J*= 12.1 Hz, 1H), 1.65-1.57 (m, 5H), 1.42-1.37 (m, 2H). ^{13}C NMR (150 MHz, D2O) δ 175.0, 175.0, 175.0, 173.9, 173.4, 104.6, 103.3, 102.8, 101.9, 100.3, 99.6, 79.8, 78.9, 78.9, 77.1, 75.6, 75.5, 74.8, 74.7, 74.6, 72.9, 72.8, 72.6, 72.1, 71.8, 71.6, 70.8,

70.4, 70.0, 69.0, 68.9, 68.4, 68.3, 68.1, 67.7, 67.6, 67.4, 63.4, 62.6, 62.5, 60.9, 60.6, 60.3, 60.1, 51.9, 51.6, 51.2, 40.2, 39.7, 39.3, 28.1, 26.4, 22.3, 22.0, 22.0. HRMS (ESI-TOF, MH$^+$) calcd for C59H100N4O42: 1537.5885, found: 1537.5895.

Sialyl Gb4-C5NH2 (SGb4-NH$_2$, 4)

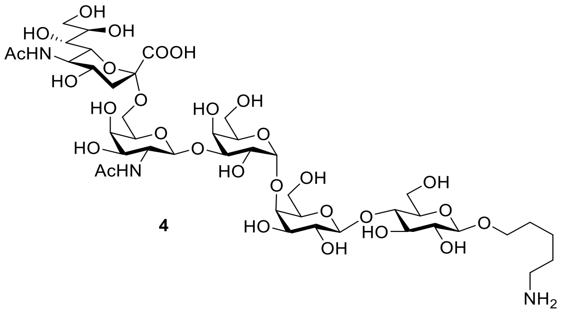

4

^{1}H NMR (600 MHz, D2O) δ 4.84 (d, *J* = 4.0 Hz, 1H), 4.55 (d, *J* = 8.0 Hz, 1H), 4.43 (dd, *J* = 8.0, 10.0 Hz, 2H), 4.32 (t, *J* = 6.4 Hz, 1H), 4.20 (d, *J* = 2.8 Hz, 1H), 3.97 (d, *J* = 3.0 Hz, 1H), 3.94-3.80 (m, 10H), 3.80-3.71 (m, 5H), 3.68-3.50 (m, 16H), 3.23 (t, *J* = 8.2, 8.7 Hz, 1H), 2.94 (t, *J* = 7.2, 7.7Hz, 2H), 2.67 (dd, *J* = 4.8, 13.0 Hz, 1H), 1.97 (s, 3H), 1.96 (s, 3H), 1.64-1.58 (m, 5H), 1.42-1.38 (m, 2H).^{13}C NMR (150 MHz, D2O) δ 175.1, 175.0, 173.4, 103.3, 103.0, 101.9, 100.4, 100.4, 78.9, 78.8, 77.2, 75.5, 74.8, 74.5, 73.2, 72.9, 72.6, 72.1, 71.7, 70.8, 70.7, 70.4, 70.0, 68.9, 68.3, 68.2, 67.7, 67.7, 63.5, 62.6, 60.6, 60.3, 60.1, 52.5, 51.9, 40.2, 39.3, 28.1, 26.4, 22.2, 22.1, 22.0. HRMS (ESI-TOF, MH$^+$) calcd for C42H73N3O29: 1084.4402, found: 1084.4405.

Sialyl Gb5-C5NH2 (SGb5-NH$_2$, 5)

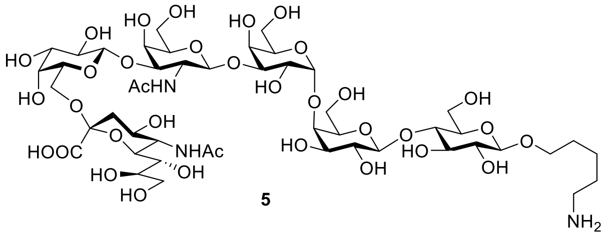

5

^{1}H NMR (600 MHz, D2O) δ 4.84 (d, *J* = 4.2 Hz, 1H), 4.62 (d, *J* = 8.5 Hz, 1H), 4.44 (dd, *J* = 7.5, 10.5 Hz, 2H), 4.37 (d, *J* = 7.5 Hz, 1H), 4.32 (t, *J* = 6.5, 6.8 Hz, 1H), 4.19 (d, *J* = 2.6 Hz, 1H), 4.11 (d, *J* = 3.3 Hz, 1H), 4.01-3.97 (m, 2H), 3.94-3.90 (m, 2H), 3.89-3.71 (m, 15H), 3.69-3.60 (m, 8H), 3.59-3.51 (m, 8H), 3.45 (dd, *J* = 7.7, 9.9 Hz, 1H), 3.23 (m, 1H), 2.94 (t, *J* = 7.6 Hz, 2H), 2.66 (dd, *J* = 4.6, 12.5 Hz, 1H), 1.97 (s, 3H), 1.96 (s, 3H), 1.66-1.57 (m, 5H), 1.42-1.37 (m, 2H). ^{13}C NMR (150 MHz, D2O) δ 175.1, 175.0, 173.4, 104.7, 103.3, 103.0, 101.9, 100.4, 100.4, 79.6, 78.8, 78.7, 77.1, 75.4, 74.8, 74.7, 74.6, 73.2, 72.9, 72.6, 72.3, 72.1, 71.7, 70.9, 70.5, 70.3, 70.0, 68.9, 68.5, 68.3, 68.2, 68.0, 67.6, 63.4, 62.6, 61.2, 60.4, 60.3, 60.1, 51.9, 51.4, 40.2, 39.3, 28.1, 26.4, 22.3, 22.1, 22.0. HRMS (ESI-TOF, MH$^+$) calcd for C48H83N3O34: 1246.4931, found: 1246.4911.

Disialyl Gb5-C5NH2 from Pd2,6ST (DSGG(Pd2,6ST)-NH$_2$, 2)

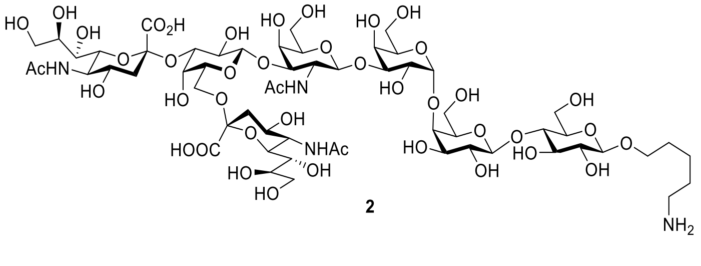

2

^{1}H NMR (600 MHz, D2O) δ 4.64 (d, *J* = 3.7 Hz, 1H), 4.71 (d, *J* = 8.8 Hz, 1H), 4.54 (m, 3H), 4.42 (t, *J* = 6.6 Hz, 1H), 4.29 (d, *J* = 2.6 Hz, 1H), 4.21 (d, *J* = 2.6 Hz, 1H), 4.12-4.06 (m, 3H), 4.03-4.00 (m, 2H), 3.99-3.80 (m, 18H), 3.78-3.55 (m, 22H), 3.33 (m, 1H), 3.05-3.00 (m, 2H), 2.79-2.74 (m, 2H), 2.06-0.05 (m, 9H), 1.81 (t, *J* = 11.8, 12.3 Hz, 1H), 1.76-1.67 (m, 5H), 1.49 (m, 2H). ^{13}C NMR (150 MHz, D2O) δ 175.1, 174.9, 173.8, 173.4, 104.4, 103.3, 103.0, 101.9, 100.4, 100.4, 99.8, 79.8, 78.9, 78.7, 77.1, 75.4, 74.8, 74.7, 74.6, 73.0, 72.9, 72.8, 72.6, 72.1, 71.8, 71.7, 70.9, 70.3, 70.0, 68.9, 68.9, 68.4, 68.3, 68.3, 68.1, 67.9, 67.6, 67.4, 63.4, 62.7, 62.5, 61.2, 60.4, 60.3, 60.1, 51.8, 51.6, 51.3, 40.1, 39.6, 39.3, 28.1, 26.4, 22.3, 22.0, 22.0. HRMS (ESI-TOF, MH^+) calcd for C59H100N4O42: 1538.5918, found: 1538.5929.

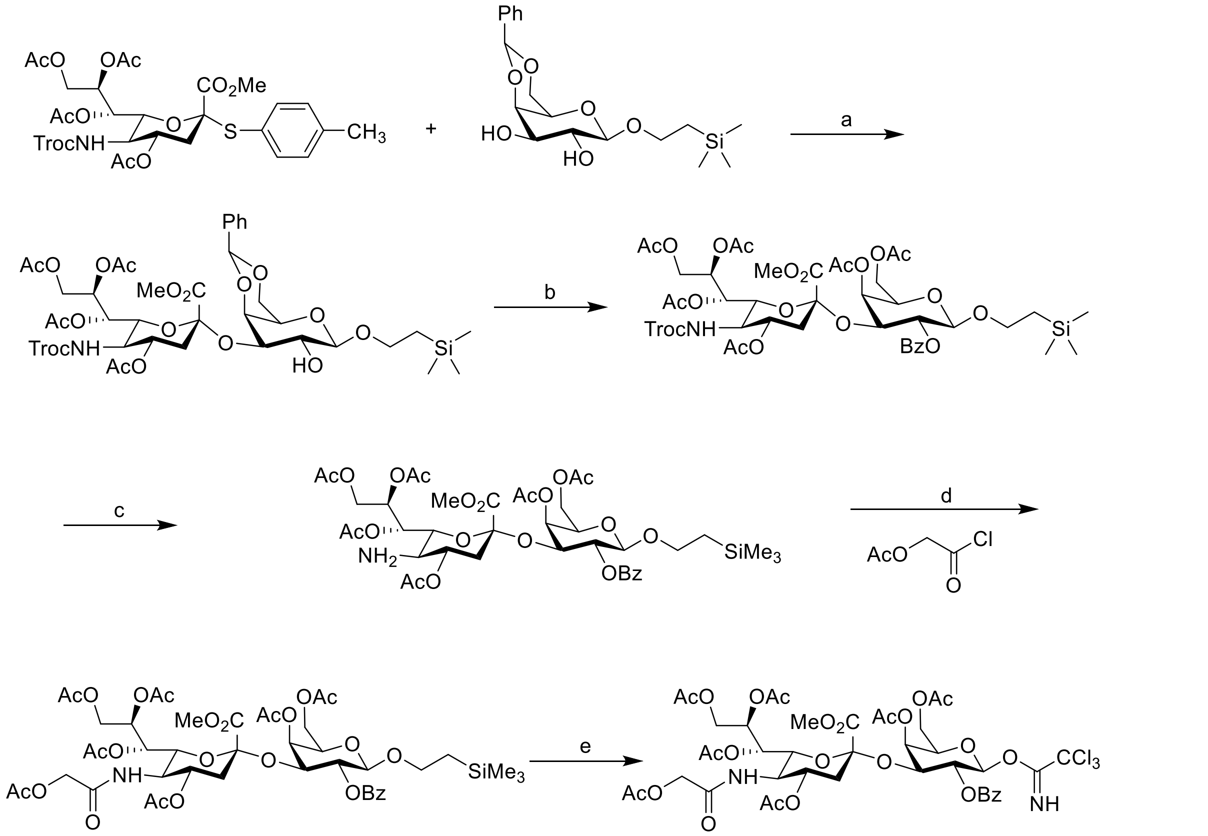

S8

Scheme S4. Synthesis of Neu5Gc disaccharide donor S8[a] [11]

[a]Reagents and conditions: (a) NIS, TfOH, MS 4A, CH_3CN/ CH_2Cl_2 (5/1), -30 to -40 °C. (b) (i) Bz_2O, Pyridine, DMAP (cat.), overnight; (ii) Pd/C, H_2, HOAc, 16h; (iii) Ac_2O, Pyridine, overnight. (c) Zn, HOAc, rt, 2 h. (d) CH_2Cl_2, Et_3N, 0 °C, 1h. (e) (i) TFA, CH_2Cl_2, (ii) CCl_3CN, DBU, CH_2Cl_2. Total yield: 65%

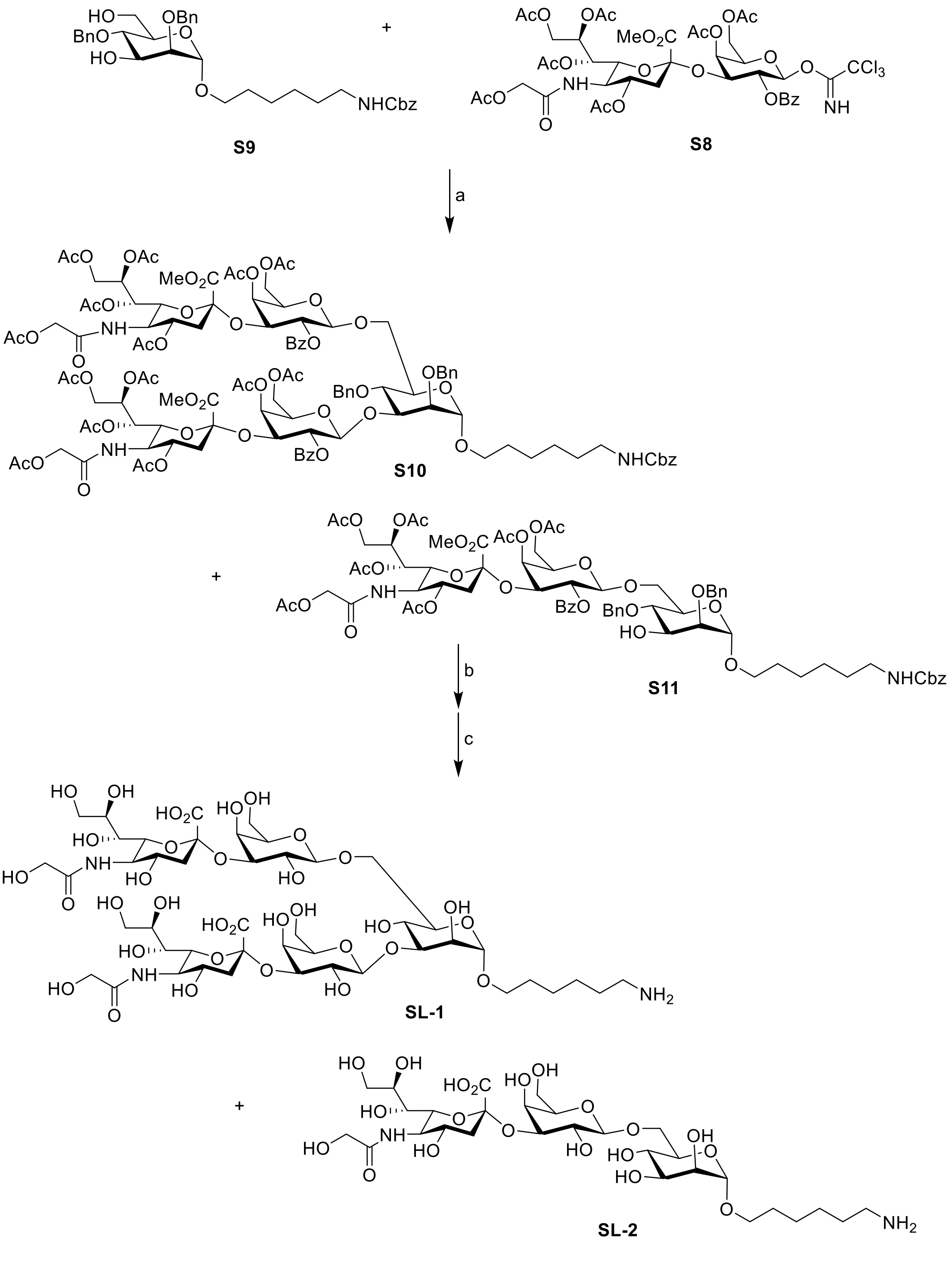

Scheme S5. Synthesis of SL-1 and SL-2[a] [12]

[a]Reagents and conditions: (a) TMSOTf, MS 4 Å, CH_2Cl_2, -45 °C to 0 °C. (b) silica gel column chromatography (50-100% EtOAc in Hexane). (c) (i) NaOMe/MeOH, then H_2O; (ii) $Pd(OH)_2/C$, H_2, $MeOH/H_2O/HOAc$.

Procedures for the synthesis of oligosaccharides SL-1 and SL-2.

To a solution of acceptor **S9** [12] (208 mg, 0.35 mmol) and donor **S8** (732 mg, 0.70 mmol) in 10 mL of dichloromethane (CH_2Cl_2) was added powdered molecular sieves (MS 4 Å, 1502 mg). The mixture was stirred at room temperature for 2 h. After cooling to -45 °C, TMSOTf (20 μL, 0.12 mmol) was added, and the mixture was allowed to slowly warm to 0 °C and stirred overnight. The reaction mixture was quenched by the addition of triethylamine (1.0 mL), diluted with CH_2Cl_2 and filtered through celite. The filtrate was washed with saturated sodium bicarbonate ($NaHCO_3$) aqueous solution, dried over sodium sulfate (Na_2SO4), filtered, and concentrated. The residue was purified by flash silica gel column chromatography (50-100% EtOAc in Hexane) to afford pentasaccharide **S10** (196 mg, 24%) and trisaccharide **S11** (47 mg, 9.14%) respectively. To a solution of **S10** in a mixture of methanol, water and acetic acid (7:2:1, 10 mL) was added 20% $Pd(OH)_2$ in Carbon (110 mg), and the reaction mixture was stirred at room temperature under a positive pressure of hydrogen for 16 h. After reaction was complete, the reaction mixture was filtered through a pad of Celite and concentrated. The residue was neutralized with ammonium hydroxide in water (28-30%), concentrated, and purified by reverse phase column chromatography (RP-18) to afford **SL-1** (24 mg, 24%). In the same manner, compound **SL-2** were obtained 5.8 mg in 28% yield from **S11.**

Neu5Gc-pentasaccharide **SL-1**:

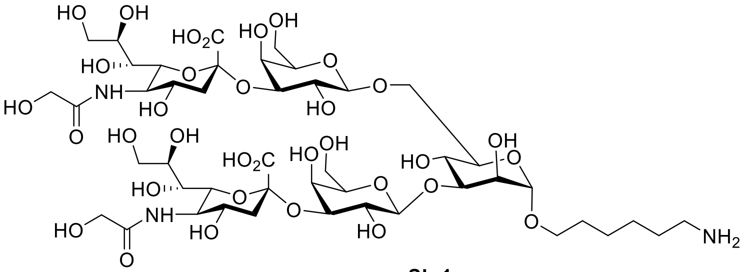

SL-1

1H NMR (400 MHz, D2O) δ 4.95 (s, 1H), 4.63 (d, *J*= 7.8, 1H), 4.57 (d, *J* = 8.0, 1H), 4.27 (d, *J* = 10.4, 1H), 4.19-4.15 (m, 6H), 4.09-4.05 (m, 1H), 4.01-3.58 (m, 30H), 3.04 (t, *J* = 7.6, 14.9, 2H), 2.85-2.81 (m, 2H), 1.86 (t, *J* = 12.0, 21.7, 2H), 1.75-1.64 (m, 4H), 1.46 (br, 4H). HRMS (ESI-TOF, MH^+) calcd for $C_{46}H_{79}N_3O_{34}$: 1217.4545, found: 1218.4649.

Neu5Gc-trisaccharide **SL-2**:

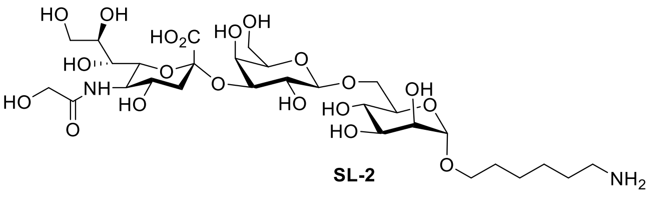

SL-2

1H NMR (400 MHz, D2O) δ 4.91 (s, 1H), 4.57 (d, *J* = 7.9 Hz, 1H), 4.25 (d, *J* = 11.0 Hz, 1H), 4.18-4.15 (m, 3H), 4.01-3.59 (m, 19H), 3.04 (t, *J* = 7.6, 15.2 Hz, 2H), 2.82 (dd, *J* = 4.7, 12.7 Hz, 1H), 1.86 (t, *J* = 12.4, 24.64, 1H), 1.70 (m, 4H), 1.45 (m, 4H). HRMS (ESI-TOF, MH^+) calcd for $C_{29}H_{52}N_2O_{20}$: 748.3113, found: 749.3201.

Table S1. α2-6-sialylation of globo-series glycans

Acceptor	Pd2,6ST			Psp2,6ST		
	Time	Product	Yield	Time	Product	Yield
Gb3 -OR	24 h	ND	--	24 h	ND	--
Gb4 -OR	7 h	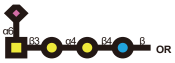 4a	70%[a]<br>83%[b]	3.5 h	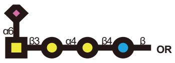 4a	42%[a]<br>55%[b]
Gb5 -OR	5 h	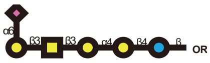 5a	69%[a]<br>97%[b]	5 h	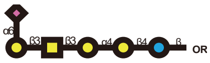 5a<br>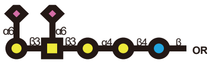 16	51%[a]<br>82%[b]<br>15%[a]
Globo H -OR	24 h	ND	--	2 h	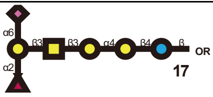 17<br>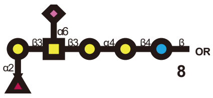 8	33%[b]<br>trace
SSEA4 -OR	2 h	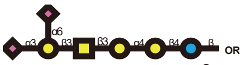 2a	67%[a]<br>100%[b]	2 h	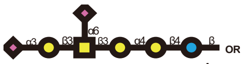 1a<br>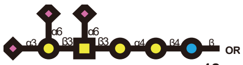 18	46%[a]<br>79%[b]<br>4%[a]

[a] isolated yield; [b] NMR yield; R=$C_5H_{10}Cl$

Table S2. Structures of glycans used in this study.

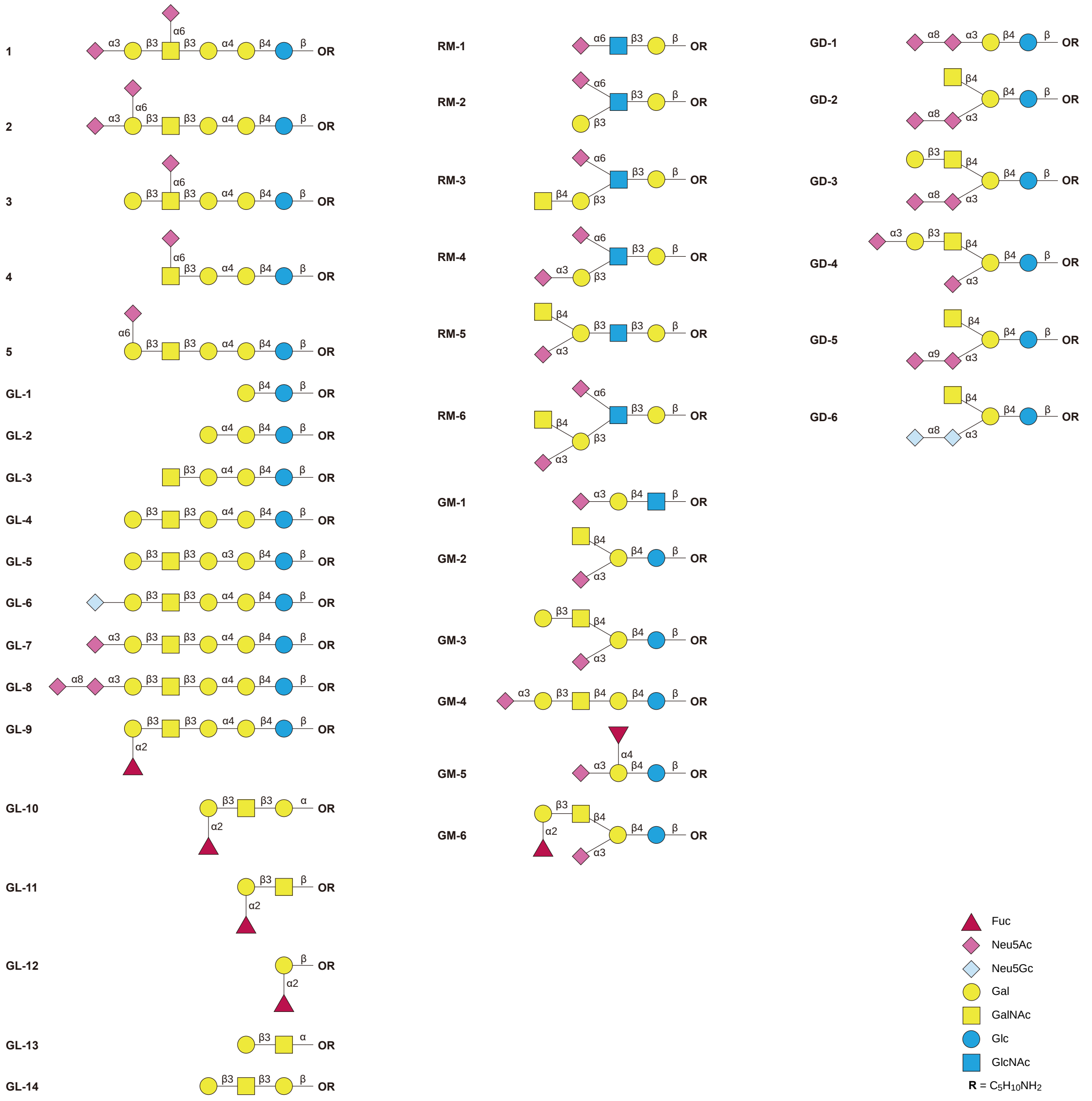

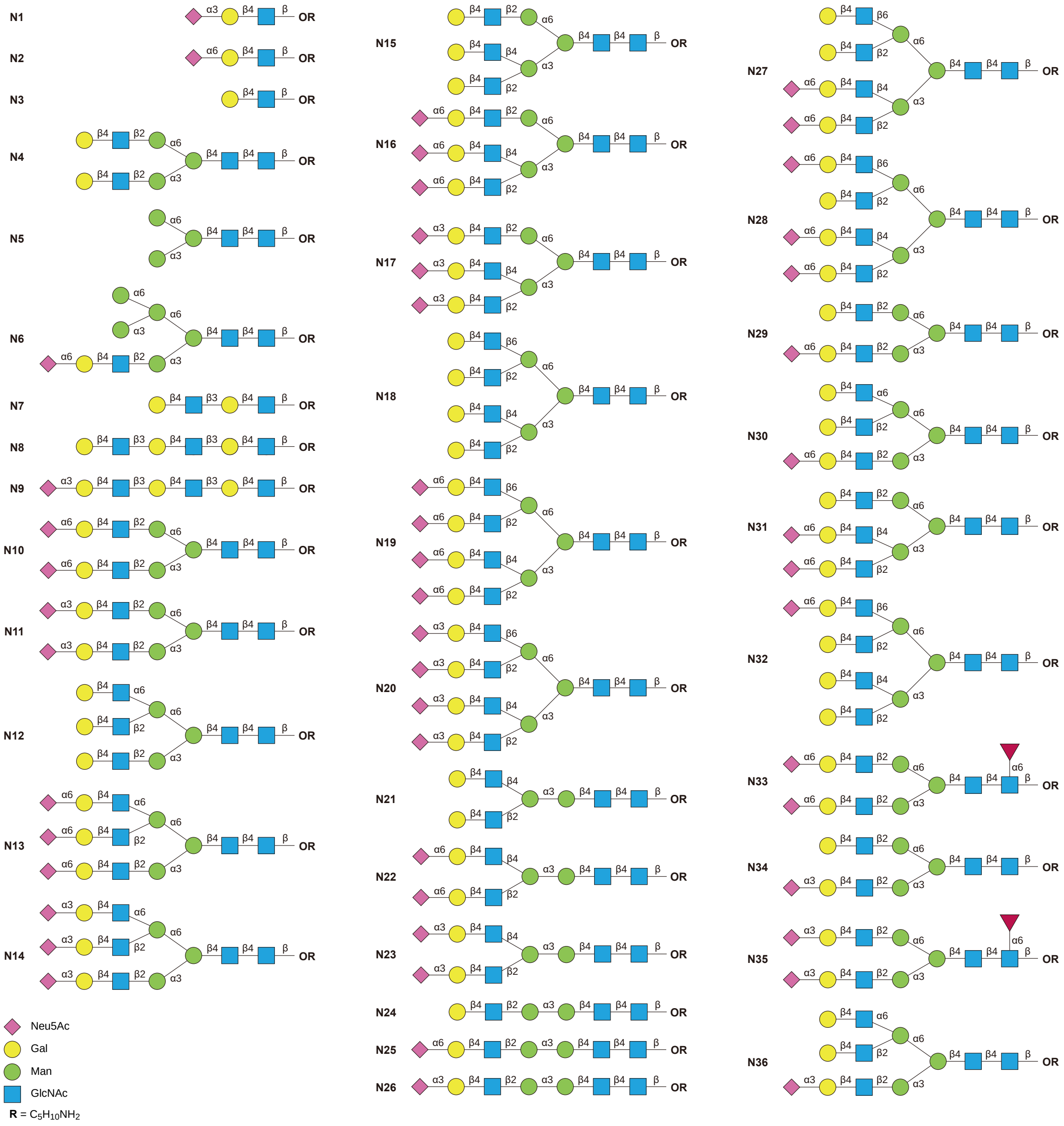
N1
N2
N3
N4
N5
N6
N7
N8
N9
N10
N11
N12
N13
N14
N15
N16
N17
N18
N19
N20
N21
N22
N23
N24
N25
N26
N27
N28
N29
N30
N31
N32
N33
N34
N35
N36
OR
Neu5Ac
Gal
Man
GlcNAc
R = $C_5H_{10}NH_2$

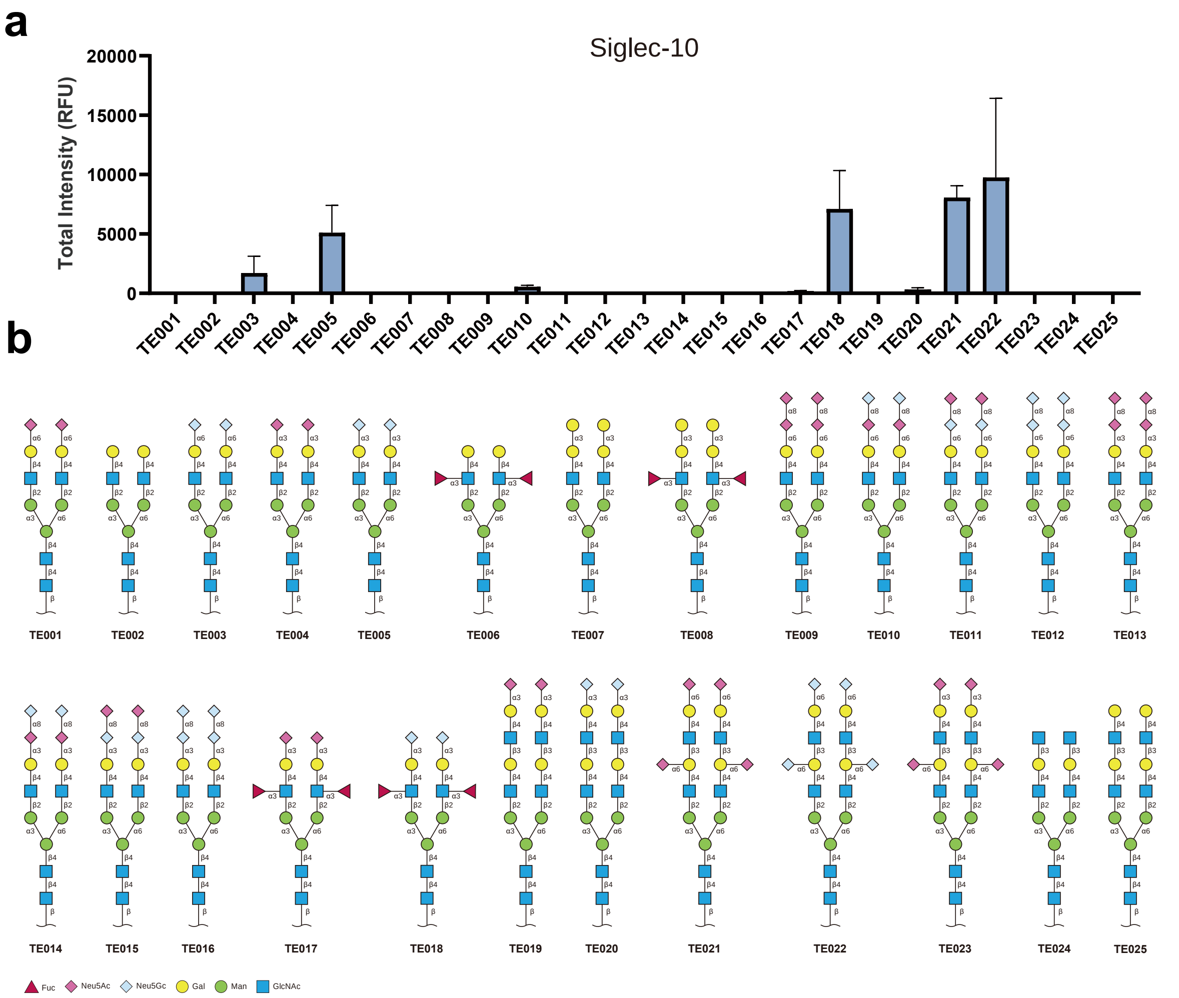

Figure S1. Commercial glycan array results (RayBiotech). Glycans on the array were treated with 12.5 µg/mL Siglec-10-Fc (R&D Systems). Error bars: standard deviation.

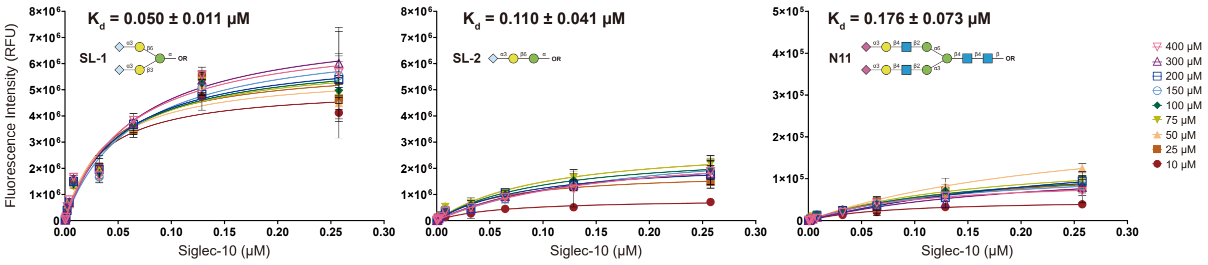

Figure S2. Binding of Siglec-10-Fc towards glycans on glycan array. Binding curves observed for glycans SL-1, SL-2 and N11 at various concentrations. Secondary antibody: Donkey anti-human IgG, Fcγ fragment specific, Alexa 647-conjugated. Error bars: standard deviation.

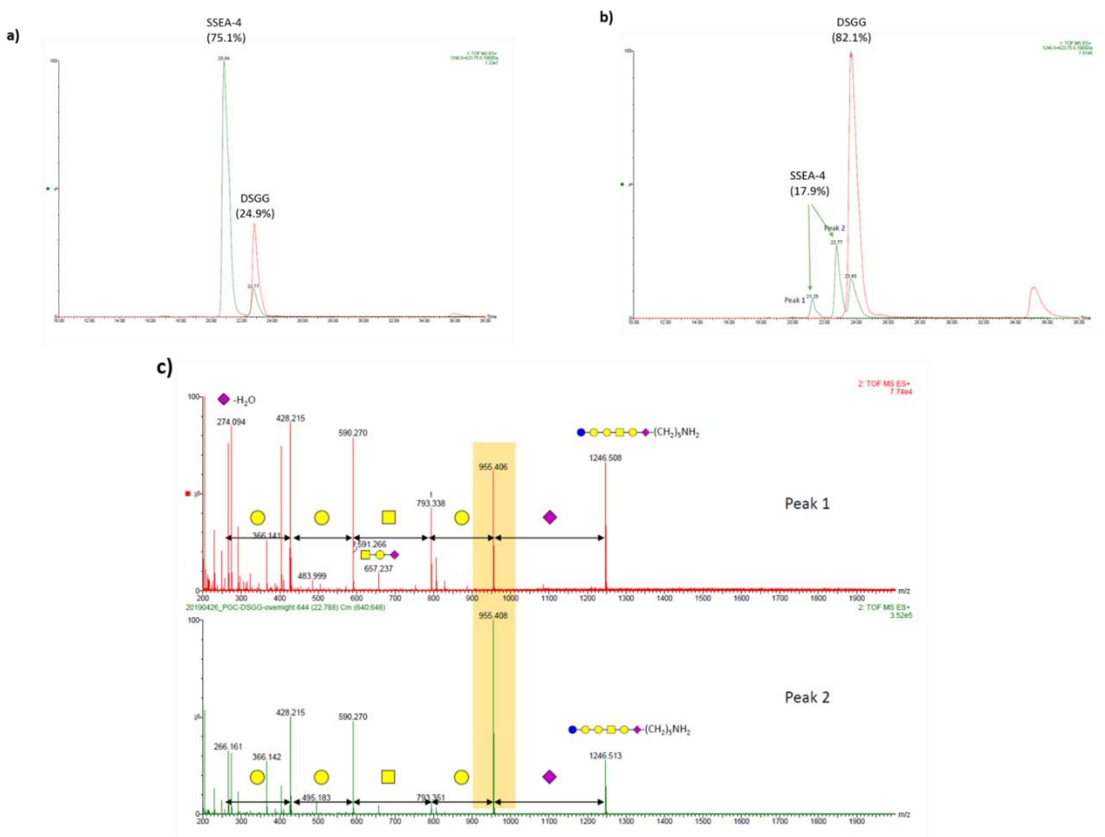
a)
SSEA-4
(75.1%)
DSGG
(24.9%)
b)
DSGG
(82.1%)
SSEA-4
(17.9%)
Peak 2
Peak 1
c)
-H2O
274.094
428.215
590.270
955.406
1246.508
793.338
366.141
591.266
483.999
657.237
Peak 1
-(CH2)5NH2
955.408
428.215
590.270
266.161
366.142
1246.513
495.183
793.351
Peak 2
m/z

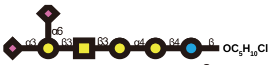
α6
α3
β3
β3
α4
β4
β
OC5H10Cl
2a

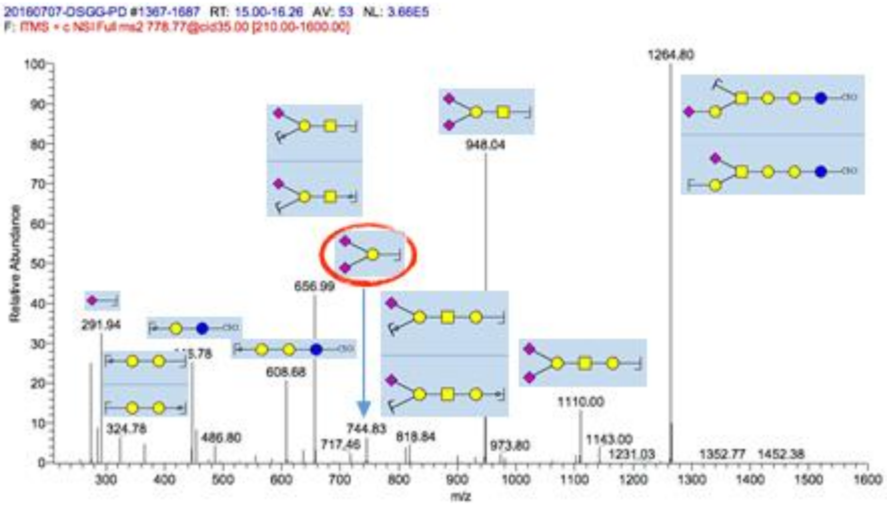
20160707-DSGG-PD #1367-1687 RT: 15.00-16.26 AV: 53 NL: 3.66E5
F: ITMS + c NSI Full ms2 778.77@cid35.00 [210.00-1600.00]
Relative Abundance
1264.80
948.04
656.99
291.94
608.68
324.78
486.80
744.83
717.46
818.84
973.80
1110.00
1143.00
1231.03
1352.77
1452.38
m/z

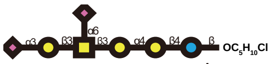
α3
β3
α6
β3
α4
β4
β
$OC_5H_{10}Cl$

1a

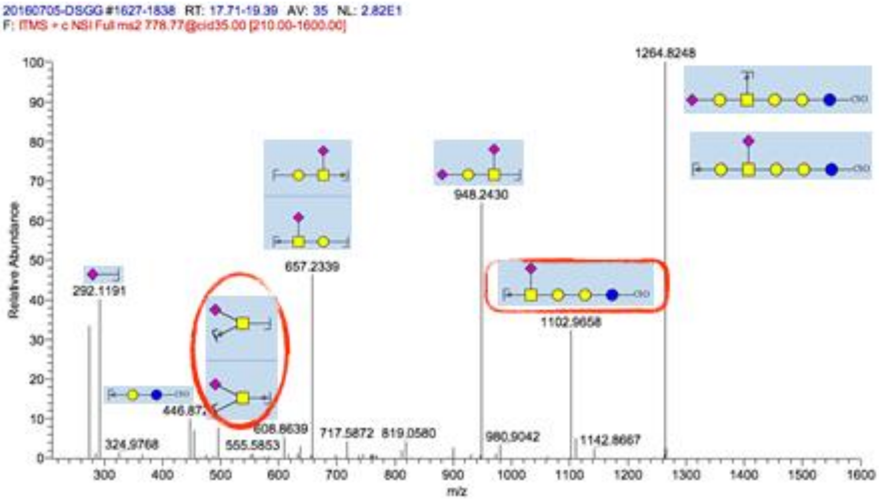
20160705-DSGG #1627-1838 RT: 17.71-19.39 AV: 35 NL: 2.82E1
F: ITMS + c NSI Full ms2 778.77@cid35.00 [210.00-1600.00]
Relative Abundance
m/z
1264.8248
948.2430
657.2339
292.1191
1102.9658
446.87
608.8639
555.5853
324.9768
717.5872
819.0580
980.9042
1142.8667

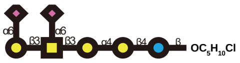
α6
β3
α6
β3
α4
β4
β
$OC_5H_{10}Cl$

16

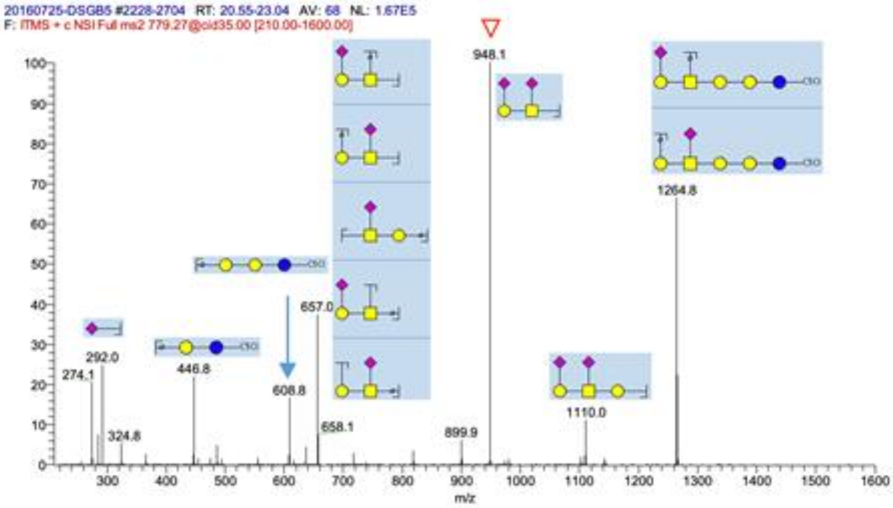
20160725-DSGB5 #2228-2704 RT: 20.55-23.04 AV: 68 NL: 1.67E5
F: ITMS + c NSI Full ms2 779.27@cid35.00 [210.00-1600.00]
m/z
948.1
1264.8
657.0
292.0
274.1
446.8
608.8
324.8
658.1
899.9
1110.0

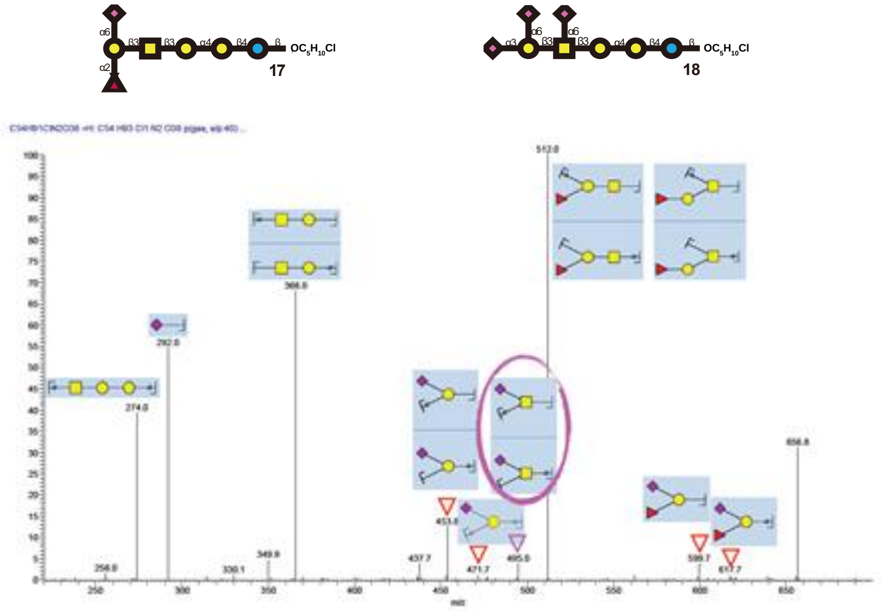

Figure S3. Structural determination of DSGG and other synthesized glycans

According to the MS/MS data analysed by GlycoWorkbench, fragment 744.8 of compound **2a** indicated that these two sialic acids connected to the same Gal. The fragments 495.09 and 1102.88 compound **1a** suggested that the new sialic acid is connected to GalNAc. When Psp2,6ST was used for the sialylation with Gb5, the monosialylated compound **5a** and disialylated compound **16** were obtained. The peak 948.1 of compound **16** of MS/MS data suggested that the second sialic acid was connected to GalNAc. For GH sialylation by Psp2,6ST, we got a monosialylated GH which was not pure compound when checked by NMR. Analysis with MS/MS data, peaks 453.8, 471.7, 599.7 and 617.7 showed sialic acid and fucose linked to the same Gal (compound **17**). However, peak 495.0 indicated that sialic acid was linked to GalNAc. According to the MS/MS data and NMR, the mixture product should include compound **17** and **8**.

NMR data

Abbreviations:

Gb4-Pd-2h: enzymatic sialylation of Gb4 with Pdp2,6ST for 2 h.

Gb4-Psp-2h: enzymatic sialylation of Gb4 with Psp2,6ST for 2 h.

GH: globo-H glycan. Lac: lactose. Slac: sialyl lactose

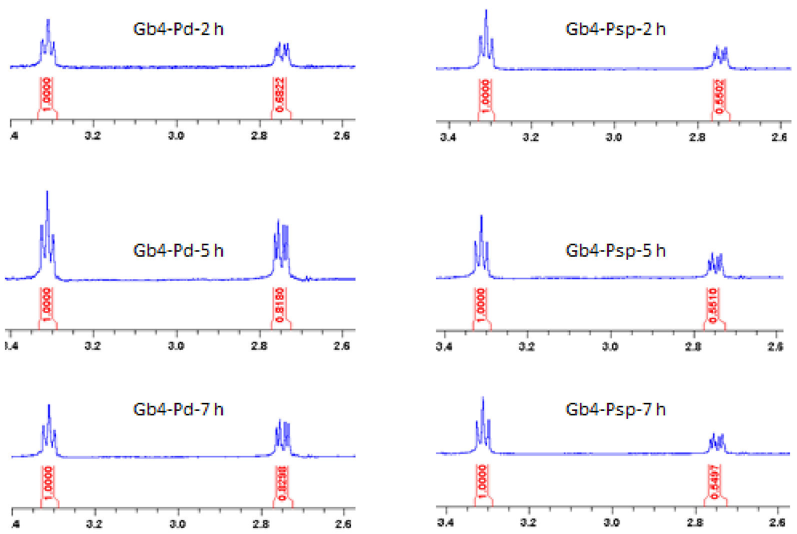

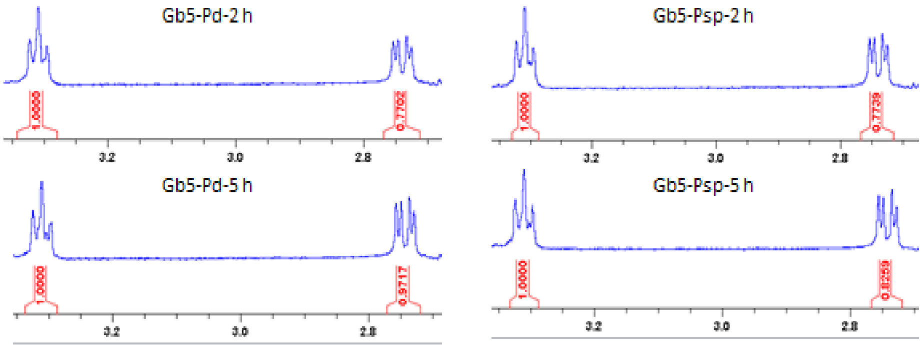

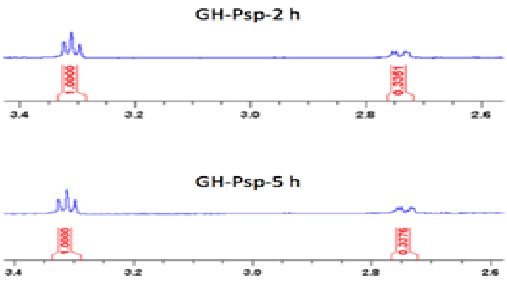

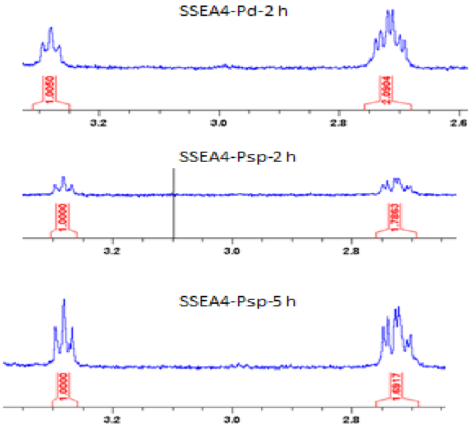

Lac-Pd-20 min

Lac-Psp-20 min

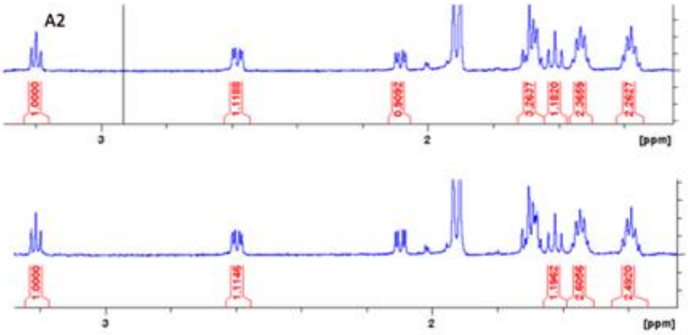

SLac-Pd-20 min

SLac-Psp-20 min
yield ~62%

SLac-Psp-40 min

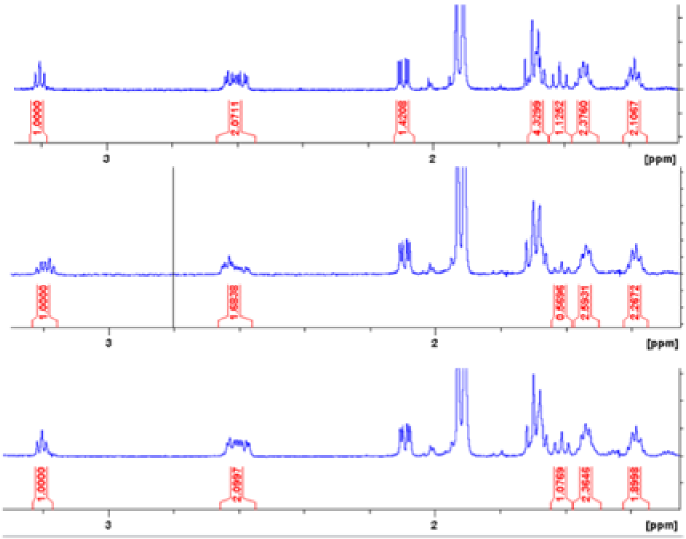

Compound S2

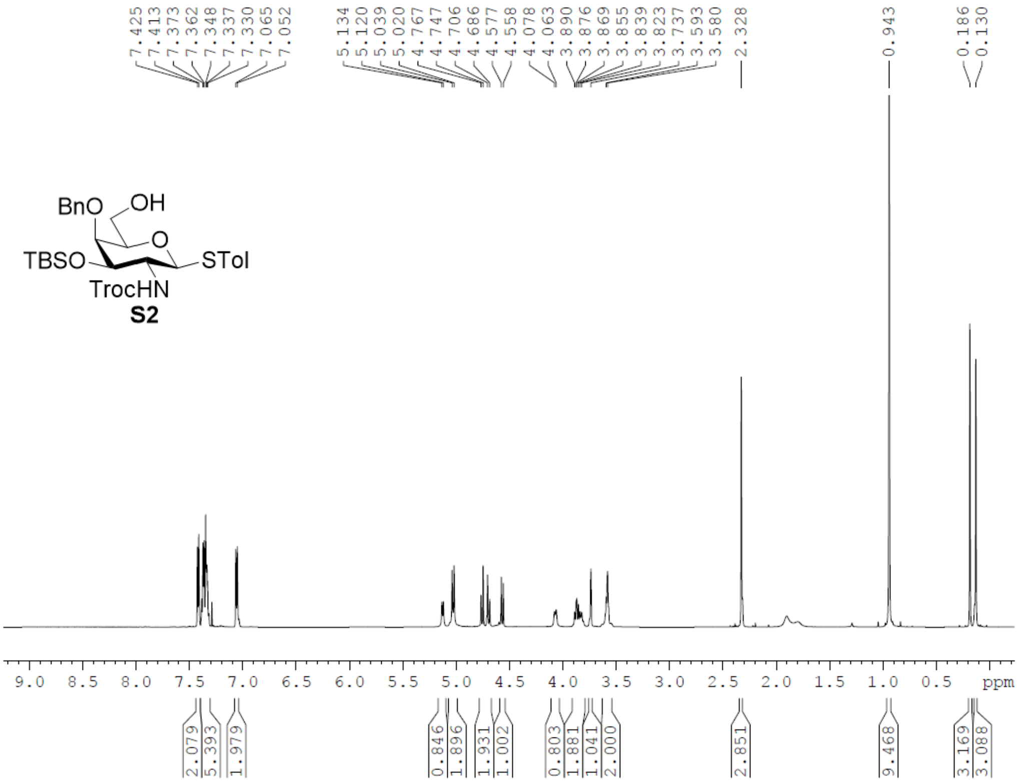
7.425
7.413
7.373
7.362
7.348
7.337
7.330
7.065
7.052
5.134
5.120
5.039
5.020
4.767
4.747
4.706
4.686
4.577
4.558
4.078
4.063
3.890
3.876
3.869
3.855
3.839
3.823
3.737
3.593
3.580
2.328
0.943
0.186
0.130
BnO
OH
O
TBSO
STol
TrocHN
S2
ppm
2.079
5.393
1.979
0.846
1.896
1.931
1.002
0.803
1.881
1.041
2.000
2.851
9.468
3.169
3.088

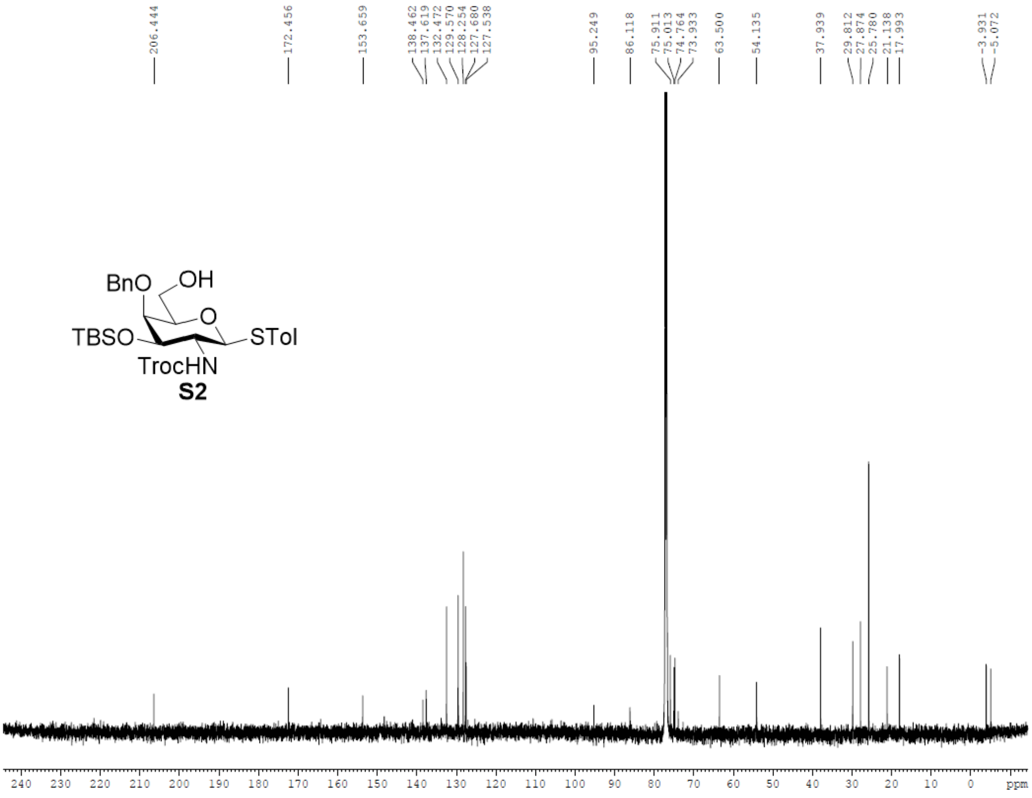
206.444
172.456
153.659
138.462
137.619
132.472
129.570
128.254
127.680
127.538
95.249
86.118
75.911
75.013
74.764
73.933
63.500
54.135
37.939
29.812
27.874
25.780
21.138
17.993
-3.931
-5.072
BnO
OH
O
TBSO
STol
TrocHN
S2
ppm

Compound S3

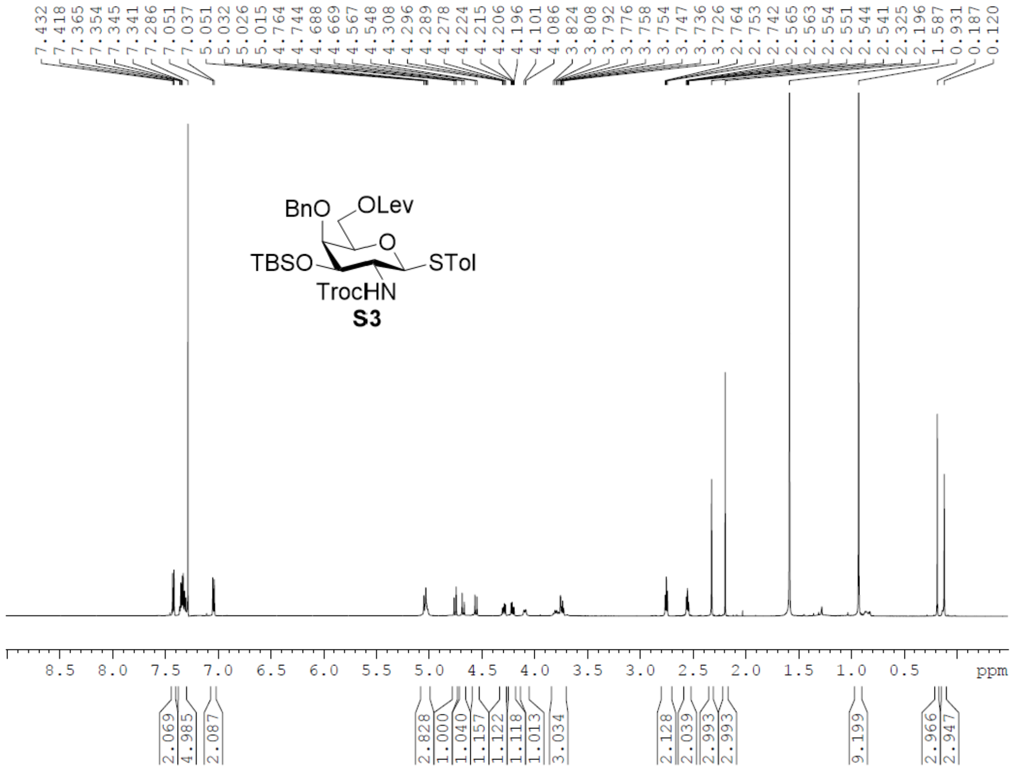
BnO
OLev
O
TBSO
STol
TrocHN
S3
ppm

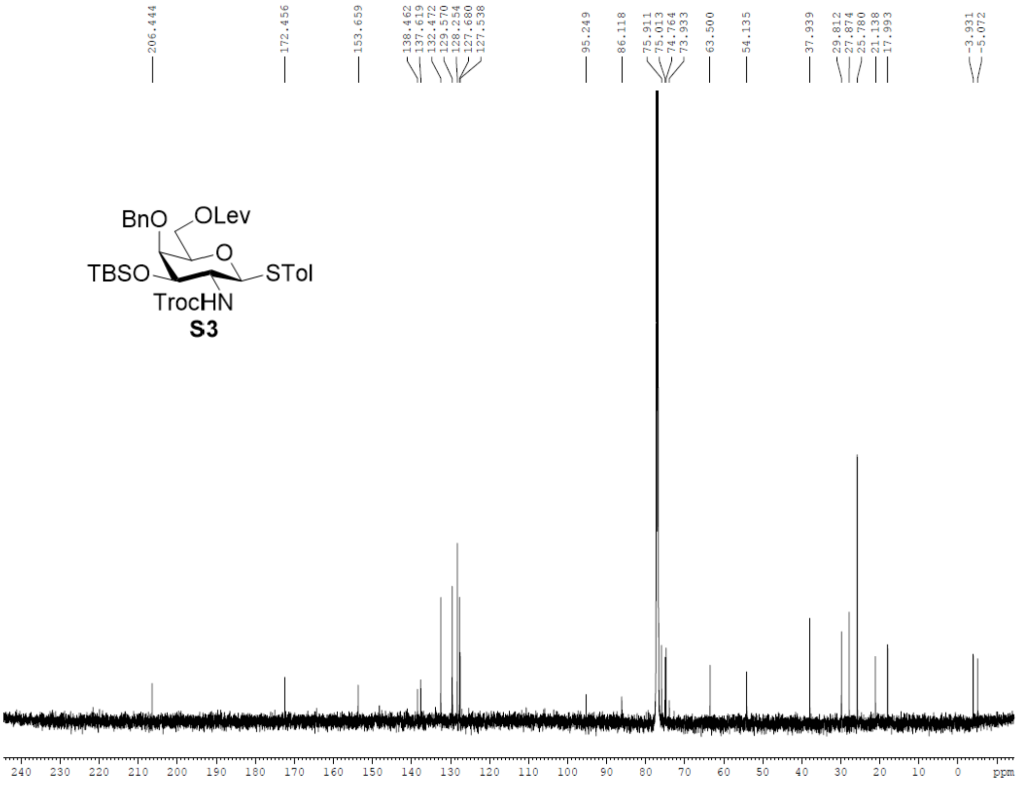
BnO
OLev
O
TBSO
STol
TrocHN
S3
ppm

Compound S4

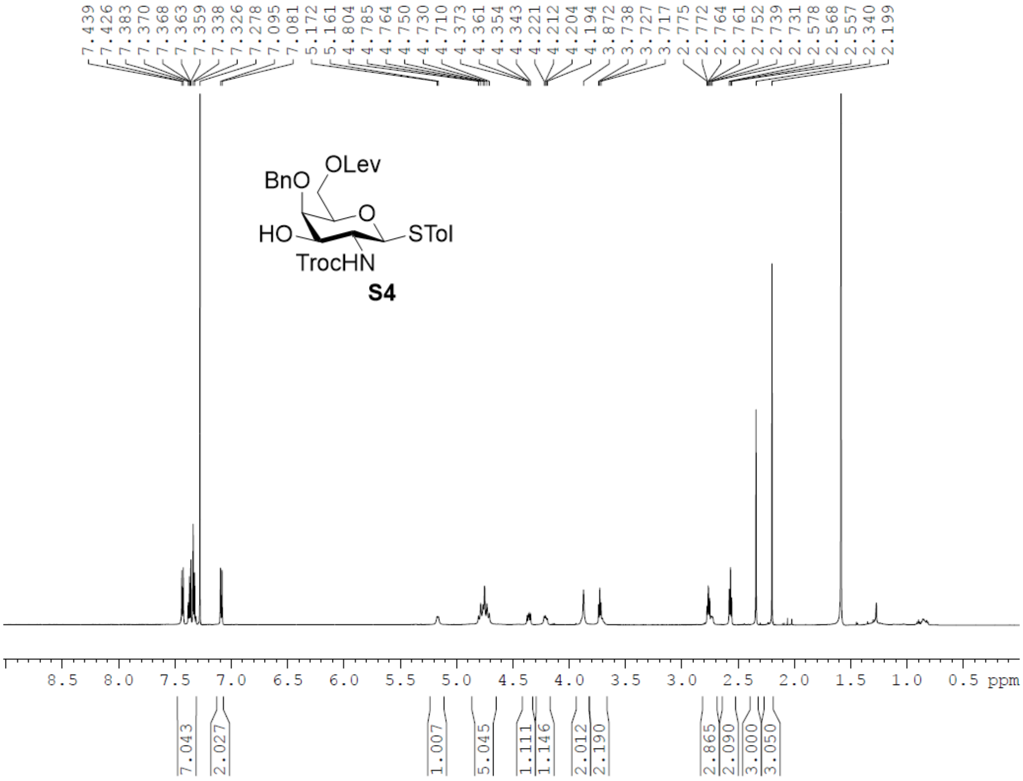

7.439
7.426
7.383
7.370
7.368
7.363
7.359
7.338
7.326
7.278
7.095
7.081
5.172
5.161
4.804
4.785
4.764
4.750
4.730
4.710
4.373
4.361
4.354
4.343
4.221
4.212
4.204
4.194
3.872
3.738
3.727
3.717
2.775
2.772
2.764
2.761
2.752
2.739
2.731
2.578
2.568
2.557
2.340
2.199
BnO
OLev
O
HO
STol
TrocHN
S4
8.5 8.0 7.5 7.0 6.5 6.0 5.5 5.0 4.5 4.0 3.5 3.0 2.5 2.0 1.5 1.0 0.5 ppm
7.043
2.027
1.007
5.045
1.111
1.146
2.012
2.190
2.865
2.090
3.000
3.050

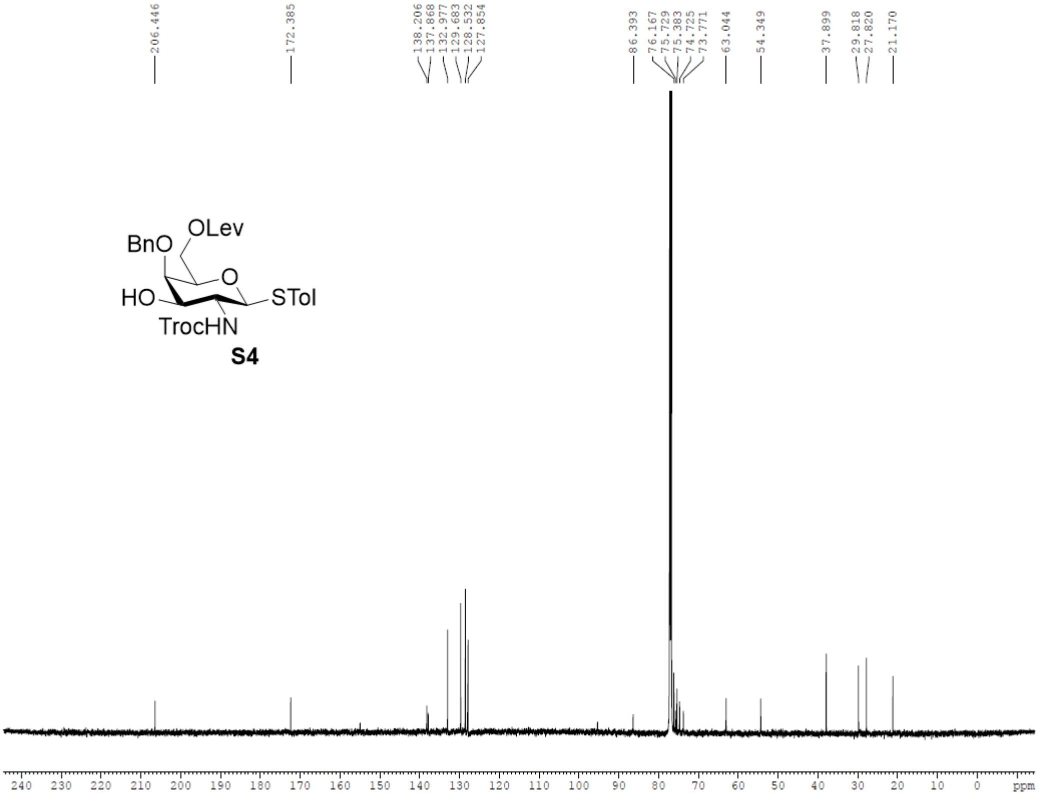

206.446
172.385
138.206
137.868
132.977
129.683
128.532
127.854
86.393
76.167
75.729
75.383
74.725
73.771
63.044
54.349
37.899
29.818
27.820
21.170
BnO
OLev
O
HO
STol
TrocHN
S4
240 230 220 210 200 190 180 170 160 150 140 130 120 110 100 90 80 70 60 50 40 30 20 10 0 ppm

Compound S7

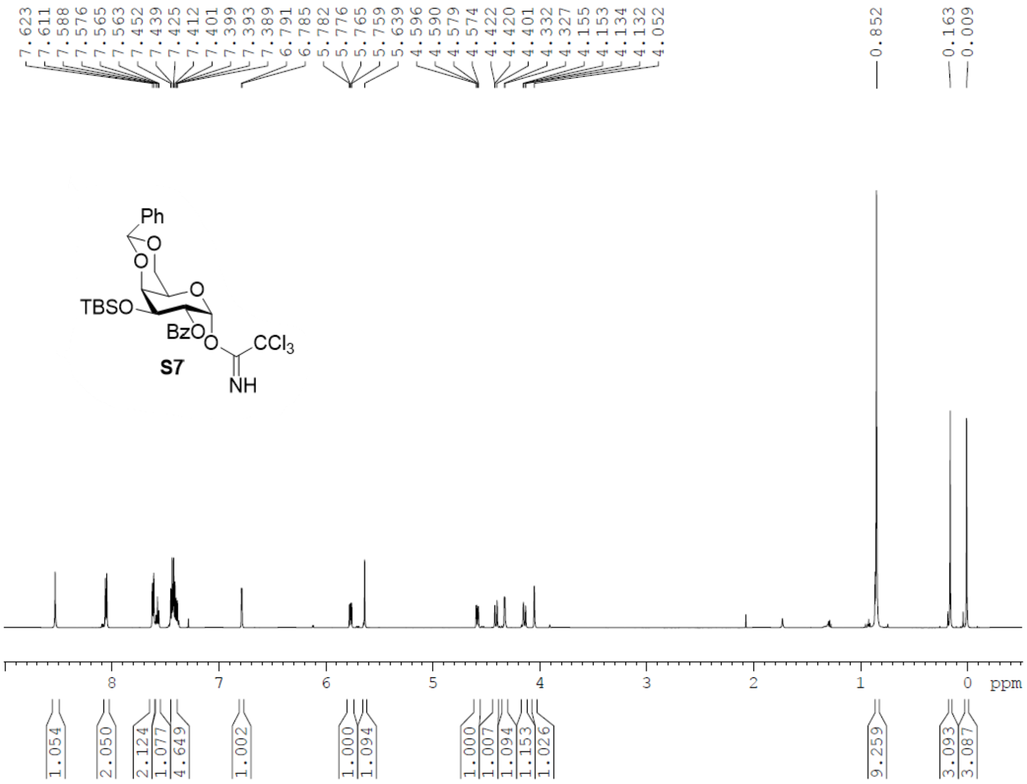
7.623 7.611 7.588 7.576 7.565 7.563 7.452 7.439 7.425 7.412 7.401 7.399 7.393 7.389 6.791 6.785 5.782 5.776 5.765 5.759 5.639 4.596 4.590 4.579 4.574 4.422 4.420 4.401 4.332 4.327 4.155 4.153 4.134 4.132 4.052
0.852
0.163 0.009
Ph
O
O
O
TBSO
BzO
O
CCl3
S7
NH
8 7 6 5 4 3 2 1 0 ppm
1.054 2.050 2.124 1.077 4.649 1.002 1.000 1.094 1.000 1.007 1.094 1.153 1.026 9.259 3.093 3.087

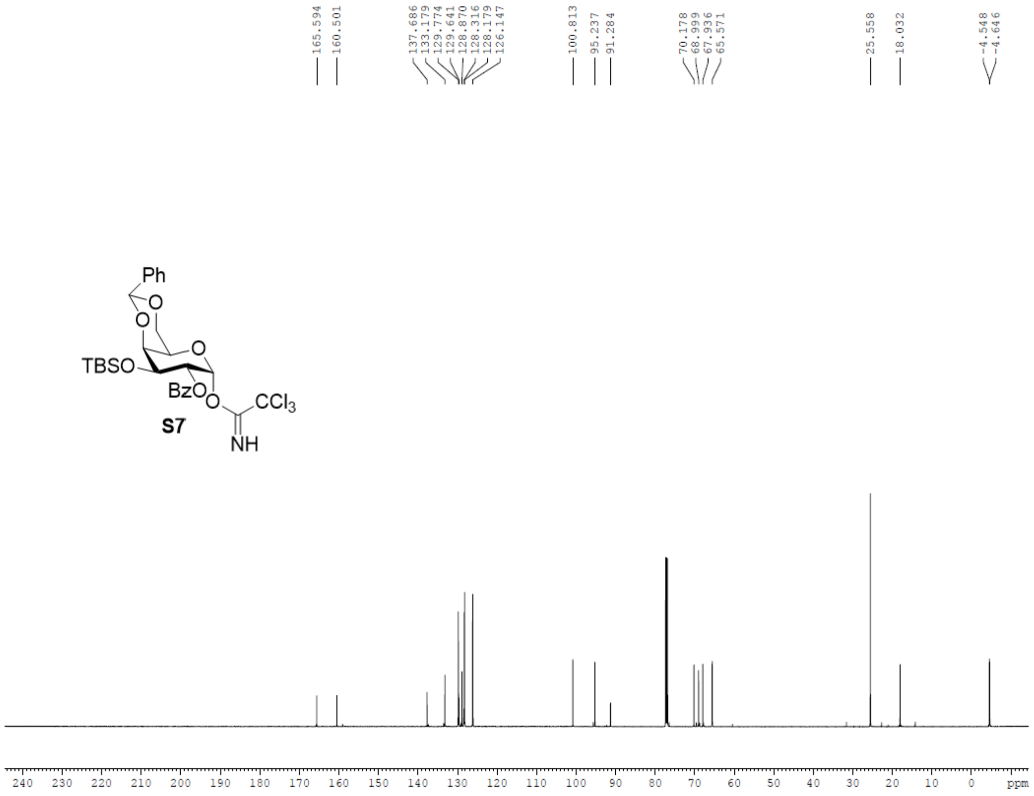
165.594 160.501
137.686 133.179 129.774 129.641 128.870 128.316 128.179 126.147
100.813 95.237 91.284
70.178 68.999 67.936 65.571
25.558 18.032
-4.548 -4.646
Ph
O
O
O
TBSO
BzO
O
CCl3
S7
NH
240 230 220 210 200 190 180 170 160 150 140 130 120 110 100 90 80 70 60 50 40 30 20 10 0 ppm

Compound 7

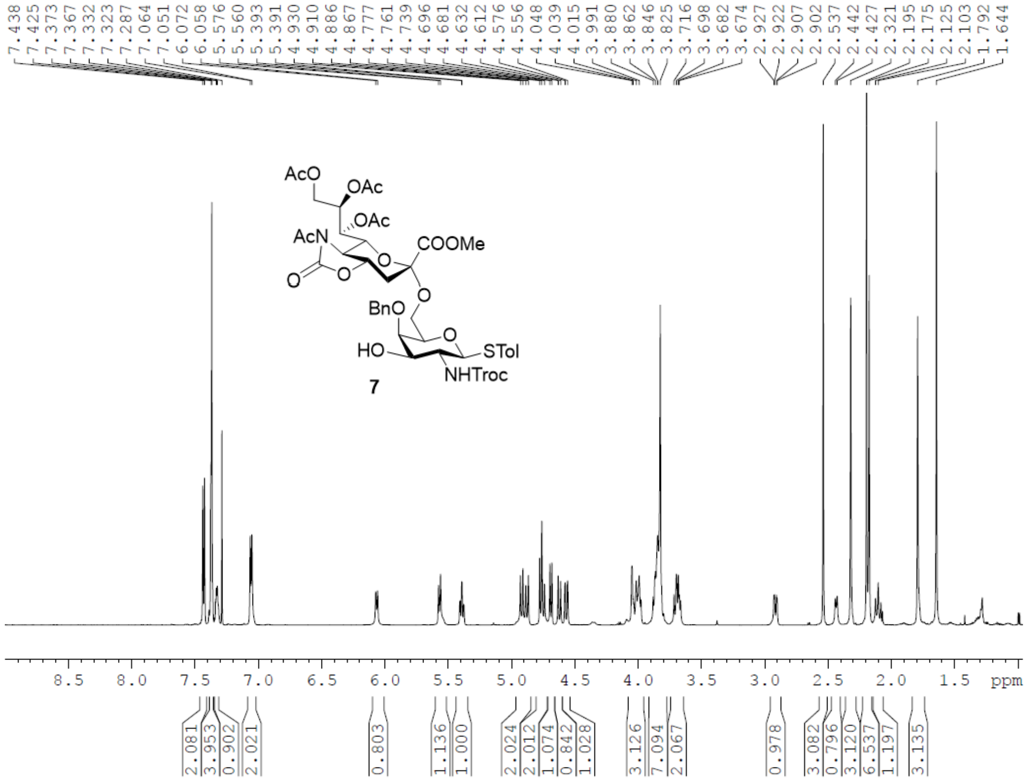
7.438
7.425
7.373
7.367
7.332
7.323
7.287
7.064
7.051
6.072
6.058
5.576
5.560
5.393
5.391
4.930
4.910
4.886
4.867
4.777
4.761
4.739
4.696
4.681
4.632
4.612
4.576
4.556
4.048
4.039
4.015
3.991
3.880
3.862
3.846
3.825
3.716
3.698
3.682
3.674
2.927
2.922
2.907
2.902
2.537
2.442
2.427
2.321
2.195
2.175
2.125
2.103
1.792
1.644
AcO
OAc
OAc
AcN
COOMe
O
O
O
O
BnO
O
HO
STol
NHTroc
7
8.5 8.0 7.5 7.0 6.5 6.0 5.5 5.0 4.5 4.0 3.5 3.0 2.5 2.0 1.5 ppm
2.081
3.953
0.902
2.021
0.803
1.136
1.000
2.024
2.012
1.074
0.842
1.028
3.126
7.094
2.067
0.978
3.082
0.796
3.120
6.537
1.197
3.135

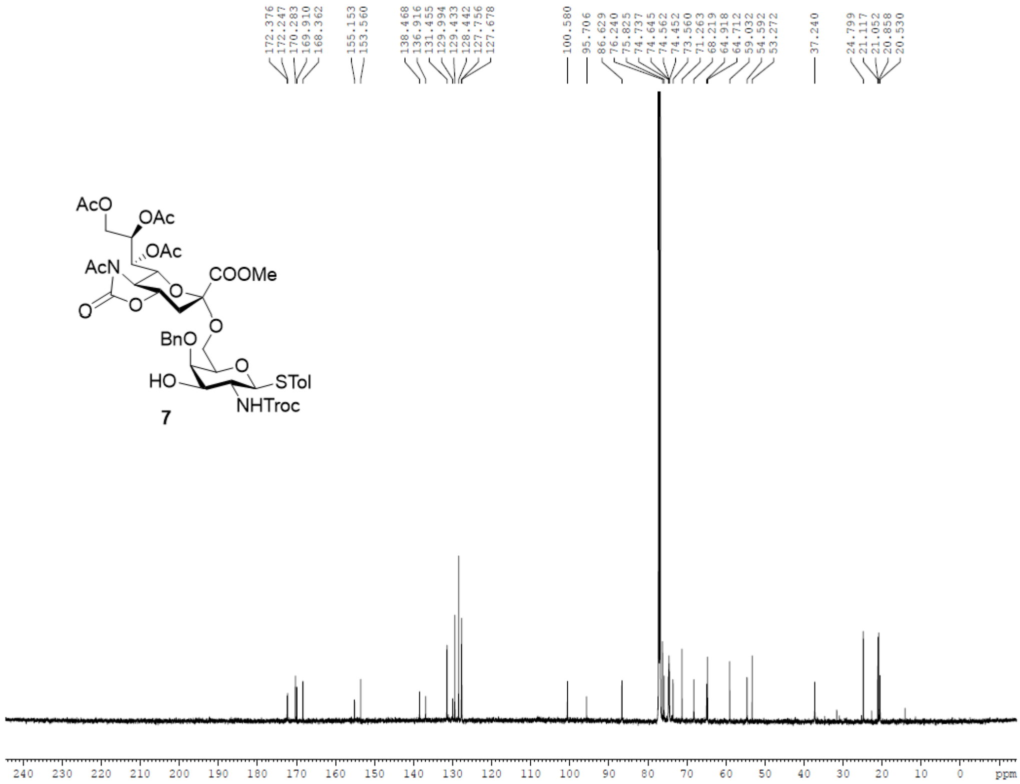
172.376
172.247
170.283
169.910
168.362
155.153
153.560
138.468
136.916
131.455
129.994
129.433
128.442
127.756
127.678
100.580
95.706
86.629
76.240
75.825
74.737
74.645
74.562
74.452
73.560
71.263
68.219
64.918
64.712
59.032
54.592
53.272
37.240
24.799
21.117
21.052
20.858
20.530
AcO
OAc
OAc
AcN
COOMe
O
O
O
O
BnO
O
HO
STol
NHTroc
7
240 230 220 210 200 190 180 170 160 150 140 130 120 110 100 90 80 70 60 50 40 30 20 10 0 ppm

Compound 9

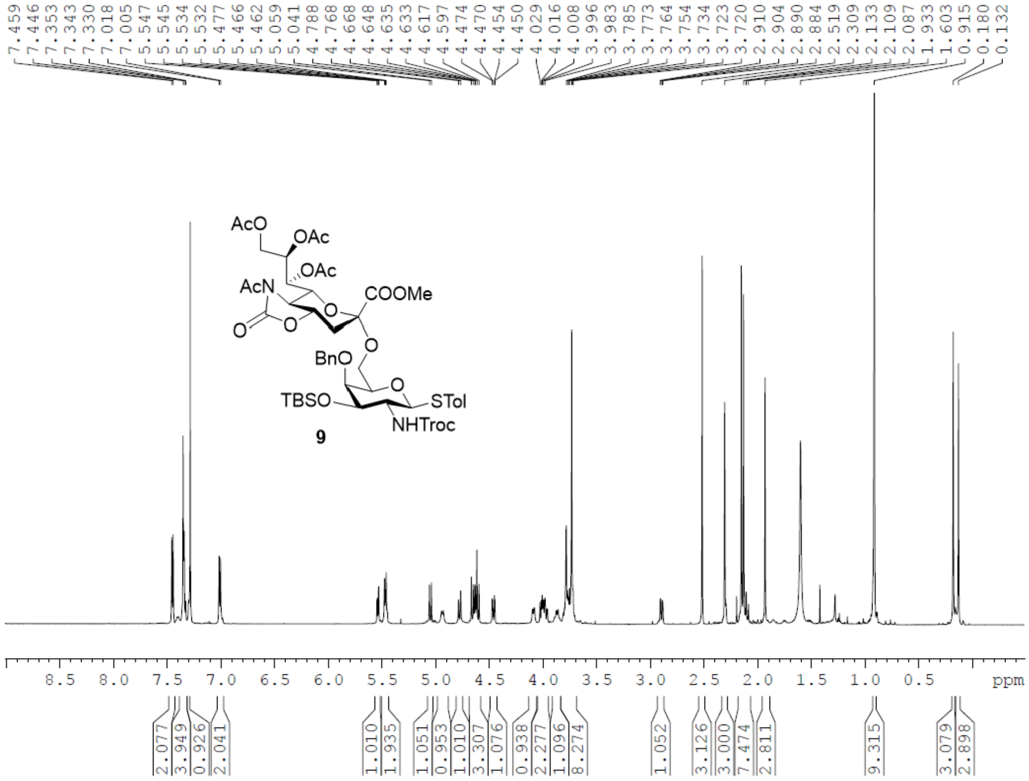
7.459 7.446 7.353 7.343 7.330 7.018 7.005 5.547 5.545 5.534 5.532 5.477 5.466 5.462 5.059 5.041 4.788 4.768 4.668 4.648 4.635 4.633 4.617 4.597 4.474 4.470 4.454 4.450 4.029 4.016 4.008 3.996 3.983 3.785 3.773 3.764 3.754 3.734 3.723 3.720 2.910 2.904 2.890 2.884 2.519 2.309 2.133 2.109 2.087 1.933 1.603 0.915 0.180 0.132
AcO
OAc
OAc
AcN
COOMe
O
BnO
TBSO
STol
NHTroc
9
8.5 8.0 7.5 7.0 6.5 6.0 5.5 5.0 4.5 4.0 3.5 3.0 2.5 2.0 1.5 1.0 0.5 ppm
2.077 3.949 0.926 2.041 1.010 1.935 1.051 0.953 1.010 3.307 1.076 0.938 2.277 1.096 8.274 1.052 3.126 3.000 7.474 2.811 9.315 3.079 2.898

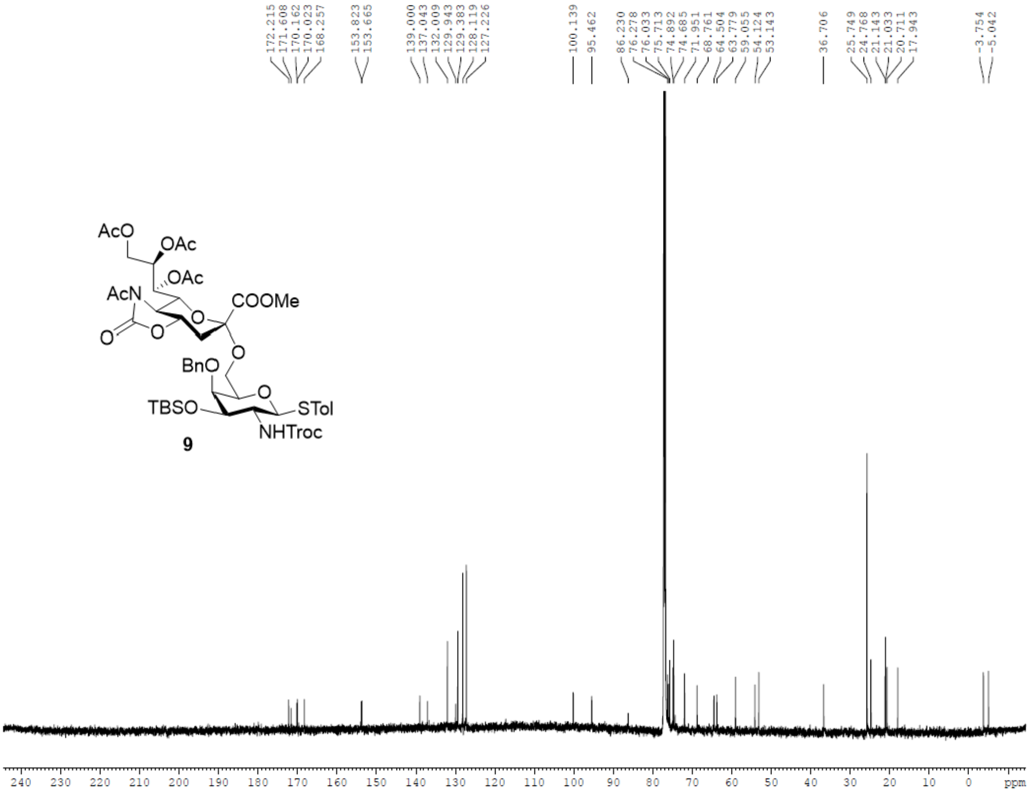
172.215 171.608 170.162 170.023 168.257 153.823 153.665 139.000 137.043 132.009 129.943 129.383 128.119 127.226 100.139 95.462 86.230 76.278 76.033 75.713 74.892 74.685 71.951 68.761 64.504 63.779 59.055 54.124 53.143 36.706 25.749 24.768 21.143 21.033 20.711 17.943 -3.754 -5.042
AcO
OAc
OAc
AcN
COOMe
O
BnO
TBSO
STol
NHTroc
9
240 230 220 210 200 190 180 170 160 150 140 130 120 110 100 90 80 70 60 50 40 30 20 10 0 ppm

Compound 2a

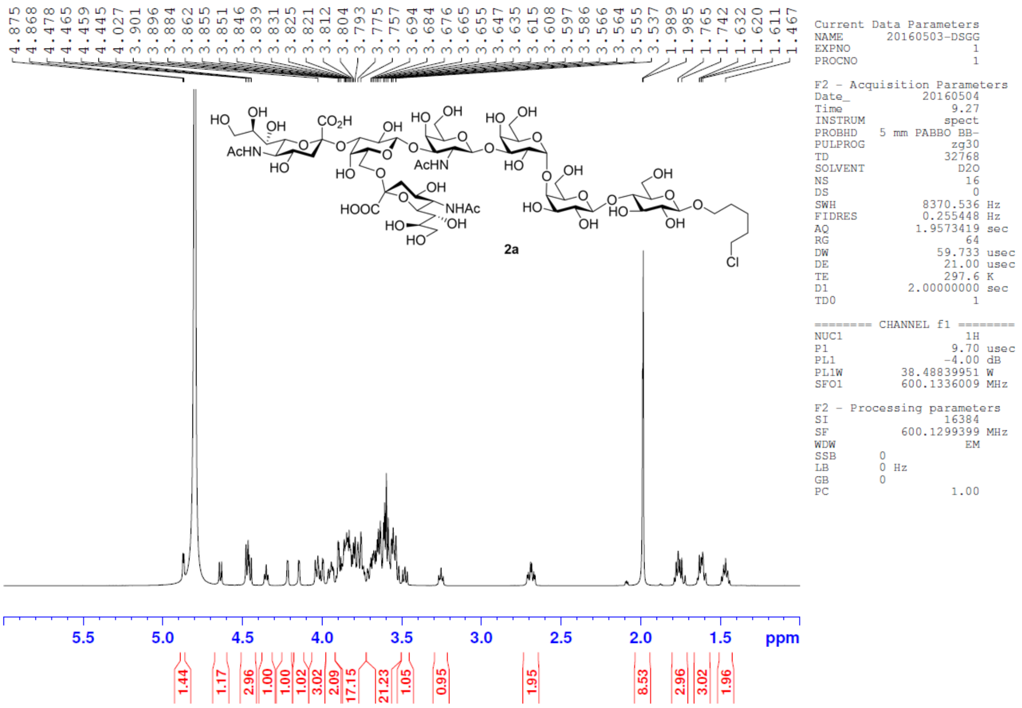
4.875 4.868 4.478 4.465 4.459 4.445 4.027 3.901 3.896 3.884 3.862 3.855 3.851 3.846 3.839 3.831 3.825 3.821 3.812 3.804 3.793 3.775 3.757 3.694 3.684 3.676 3.665 3.655 3.647 3.635 3.615 3.608 3.597 3.586 3.566 3.564 3.555 3.537 1.989 1.985 1.765 1.742 1.632 1.620 1.611 1.467
2a
Current Data Parameters
NAME 20160503-DSGG
EXPNO 1
PROCNO 1
F2 - Acquisition Parameters
Date_ 20160504
Time 9.27
INSTRUM spect
PROBHD 5 mm PABBO BB-
PULPROG zg30
TD 32768
SOLVENT D2O
NS 16
DS 0
SWH 8370.536 Hz
FIDRES 0.255448 Hz
AQ 1.9573419 sec
RG 64
DW 59.733 usec
DE 21.00 usec
TE 297.6 K
D1 2.00000000 sec
TD0 1
======== CHANNEL f1 ========
NUC1 1H
P1 9.70 usec
PL1 -4.00 dB
PL1W 38.48839951 W
SFO1 600.1336009 MHz
F2 - Processing parameters
SI 16384
SF 600.1299399 MHz
WDW EM
SSB 0
LB 0 Hz
GB 0
PC 1.00
5.5 5.0 4.5 4.0 3.5 3.0 2.5 2.0 1.5 ppm
1.44 1.17 2.96 1.00 1.00 1.02 3.02 2.09 17.15 21.23 1.05 0.95 1.95 8.53 2.96 3.02 1.96

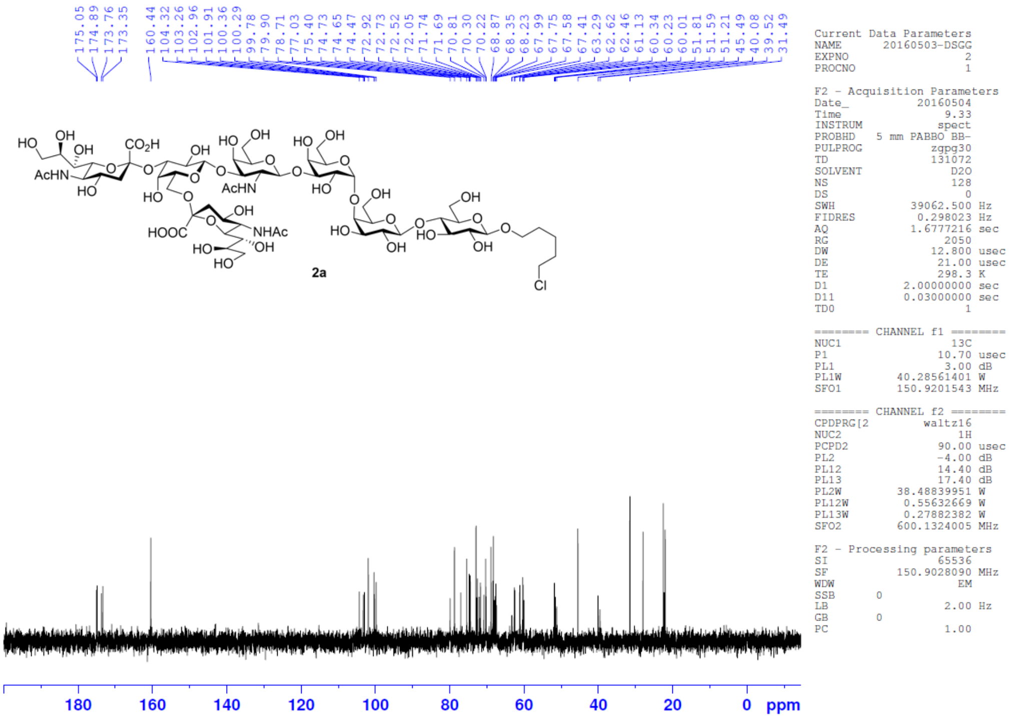
175.05 174.89 173.76 173.35 160.44 104.32 103.26 102.96 101.91 100.36 100.29 99.78 79.90 78.71 77.03 75.40 74.73 74.65 74.47 72.92 72.73 72.52 72.05 71.74 71.69 70.81 70.30 70.22 68.87 68.35 68.23 67.99 67.75 67.58 67.41 63.29 62.62 62.46 61.13 60.34 60.23 60.01 51.81 51.59 51.21 45.49 40.08 39.52 31.49
2a
Current Data Parameters
NAME 20160503-DSGG
EXPNO 2
PROCNO 1
F2 - Acquisition Parameters
Date_ 20160504
Time 9.33
INSTRUM spect
PROBHD 5 mm PABBO BB-
PULPROG zgpg30
TD 131072
SOLVENT D2O
NS 128
DS 0
SWH 39062.500 Hz
FIDRES 0.298023 Hz
AQ 1.6777216 sec
RG 2050
DW 12.800 usec
DE 21.00 usec
TE 298.3 K
D1 2.00000000 sec
D11 0.03000000 sec
TD0 1
======== CHANNEL f1 ========
NUC1 13C
P1 10.70 usec
PL1 3.00 dB
PL1W 40.28561401 W
SFO1 150.9201543 MHz
======== CHANNEL f2 ========
CPDPRG[2 waltz16
NUC2 1H
PCPD2 90.00 usec
PL2 -4.00 dB
PL12 14.40 dB
PL13 17.40 dB
PL2W 38.48839951 W
PL12W 0.55632669 W
PL13W 0.27882382 W
SFO2 600.1324005 MHz
F2 - Processing parameters
SI 65536
SF 150.9028090 MHz
WDW EM
SSB 0
LB 2.00 Hz
GB 0
PC 1.00
180 160 140 120 100 80 60 40 20 0 ppm

Compound 1a

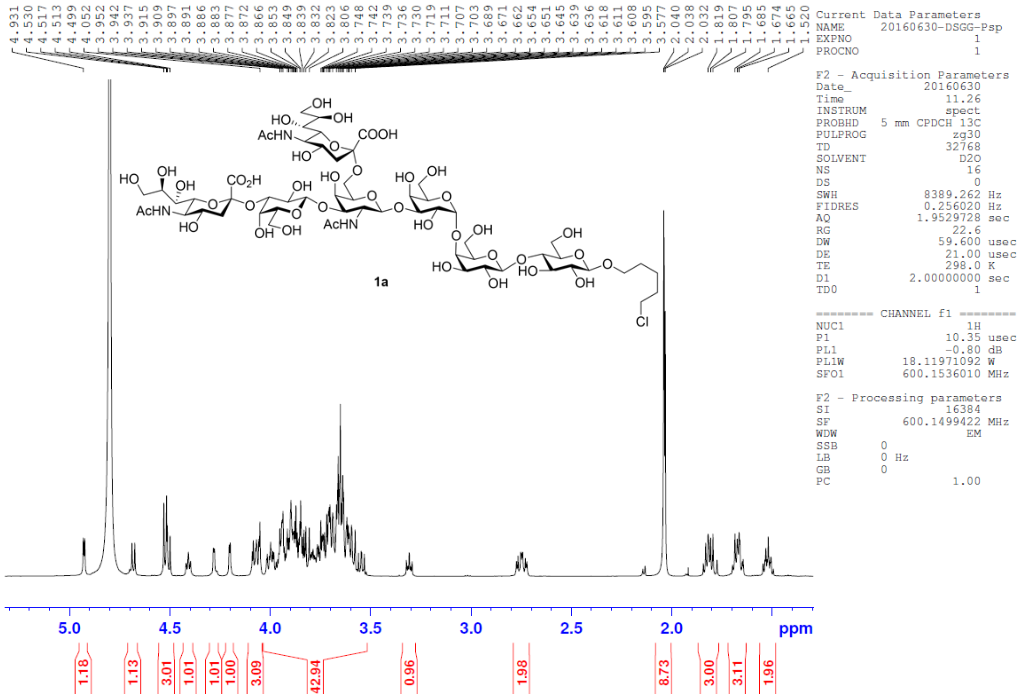

4.931 4.530 4.517 4.513 4.499 4.052 3.952 3.942 3.937 3.915 3.909 3.897 3.891 3.886 3.883 3.877 3.872 3.866 3.853 3.849 3.839 3.832 3.823 3.806 3.748 3.742 3.739 3.736 3.730 3.719 3.711 3.707 3.703 3.689 3.671 3.662 3.654 3.651 3.645 3.639 3.636 3.618 3.611 3.608 3.595 3.577 2.040 2.038 2.032 1.819 1.807 1.795 1.685 1.674 1.665 1.520
1a
Cl
5.0 4.5 4.0 3.5 3.0 2.5 2.0 ppm
1.18 1.13 3.01 1.01 1.01 1.00 3.09 42.94 0.96 1.98 8.73 3.00 3.11 1.96
Current Data Parameters
NAME 20160630-DSGG-Psp
EXPNO 1
PROCNO 1
F2 - Acquisition Parameters
Date_ 20160630
Time 11.26
INSTRUM spect
PROBHD 5 mm CPDCH 13C
PULPROG zg30
TD 32768
SOLVENT D2O
NS 16
DS 0
SWH 8389.262 Hz
FIDRES 0.256020 Hz
AQ 1.9529728 sec
RG 22.6
DW 59.600 usec
DE 21.00 usec
TE 298.0 K
D1 2.00000000 sec
TD0 1
======== CHANNEL f1 ========
NUC1 1H
P1 10.35 usec
PL1 -0.80 dB
PL1W 18.11971092 W
SFO1 600.1536010 MHz
F2 - Processing parameters
SI 16384
SF 600.1499422 MHz
WDW EM
SSB 0
LB 0 Hz
GB 0
PC 1.00

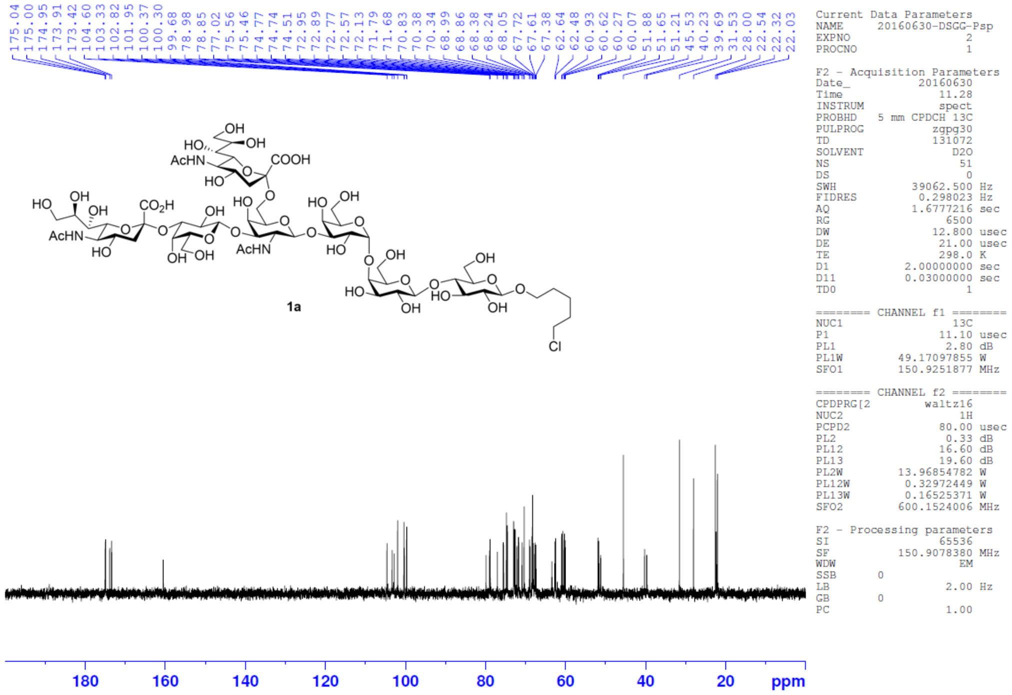

175.04 175.00 174.95 173.91 173.42 104.60 103.33 102.82 101.95 100.37 100.30 99.68 78.98 78.85 77.02 75.56 75.46 74.77 74.74 74.51 72.95 72.89 72.77 72.57 72.13 71.79 71.68 70.83 70.38 70.34 68.99 68.86 68.38 68.24 68.05 67.72 67.61 67.38 62.64 62.48 60.93 60.62 60.27 60.07 51.88 51.65 51.21 45.53 40.23 39.69 31.53 28.00 22.54 22.32 22.03
1a
Cl
180 160 140 120 100 80 60 40 20 ppm
Current Data Parameters
NAME 20160630-DSGG-Psp
EXPNO 2
PROCNO 1
F2 - Acquisition Parameters
Date_ 20160630
Time 11.28
INSTRUM spect
PROBHD 5 mm CPDCH 13C
PULPROG zgpg30
TD 131072
SOLVENT D2O
NS 51
DS 0
SWH 39062.500 Hz
FIDRES 0.298023 Hz
AQ 1.6777216 sec
RG 6500
DW 12.800 usec
DE 21.00 usec
TE 298.0 K
D1 2.00000000 sec
D11 0.03000000 sec
TD0 1
======== CHANNEL f1 ========
NUC1 13C
P1 11.10 usec
PL1 2.80 dB
PL1W 49.17097855 W
SFO1 150.9251877 MHz
======== CHANNEL f2 ========
CPDPRG[2 waltz16
NUC2 1H
PCPD2 80.00 usec
PL2 0.33 dB
PL12 16.60 dB
PL13 19.60 dB
PL2W 13.96854782 W
PL12W 0.32972449 W
PL13W 0.16525371 W
SFO2 600.1524006 MHz
F2 - Processing parameters
SI 65536
SF 150.9078380 MHz
WDW EM
SSB 0
LB 2.00 Hz
GB 0
PC 1.00

Compound 11

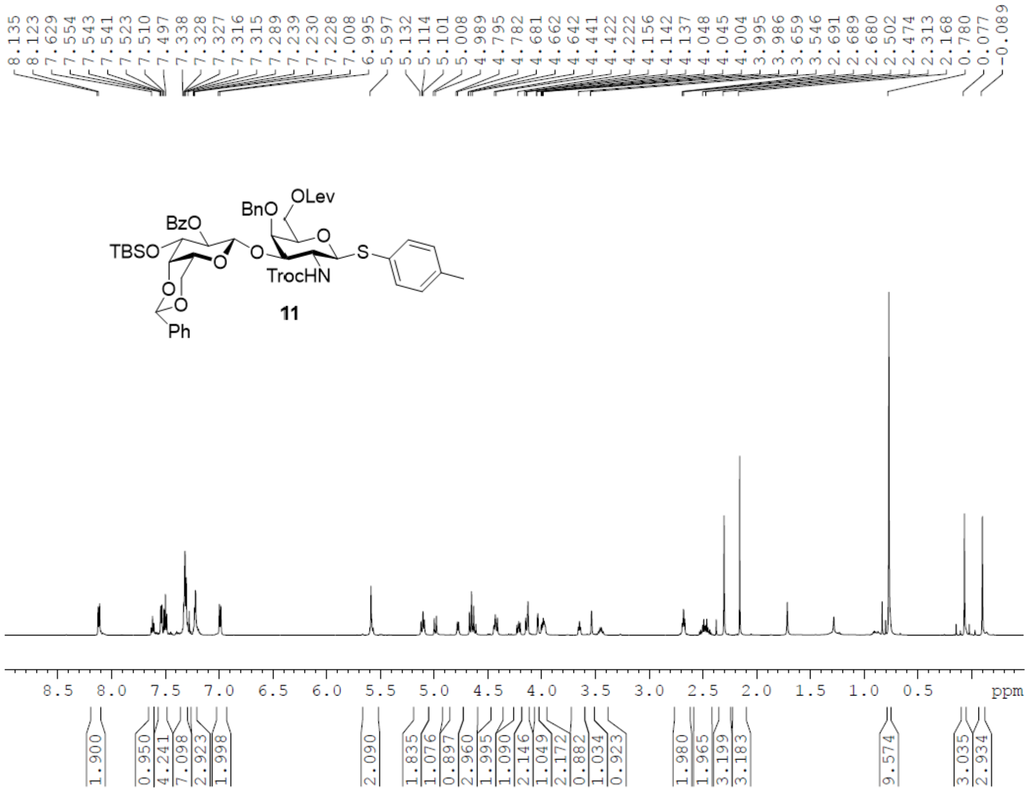
BnO
OLev
BzO
TBSO
O
O
S
TrocHN
O
O
Ph
11
ppm

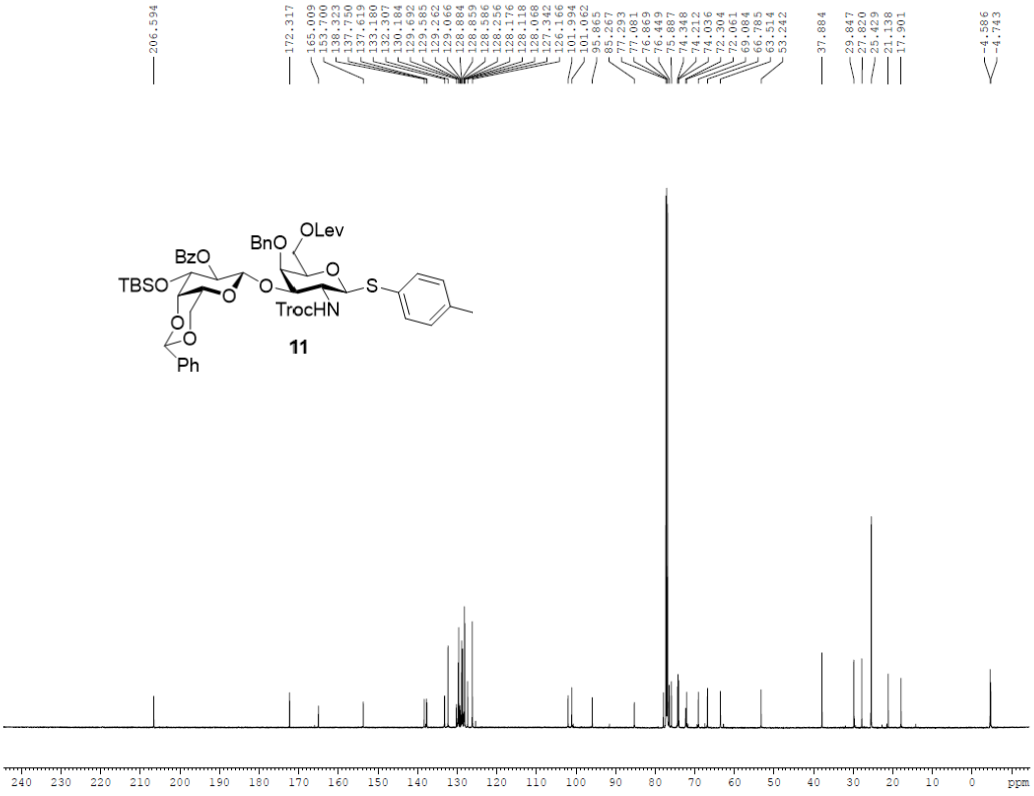
BnO
OLev
BzO
TBSO
O
O
S
TrocHN
O
O
Ph
11
ppm

Compound 12

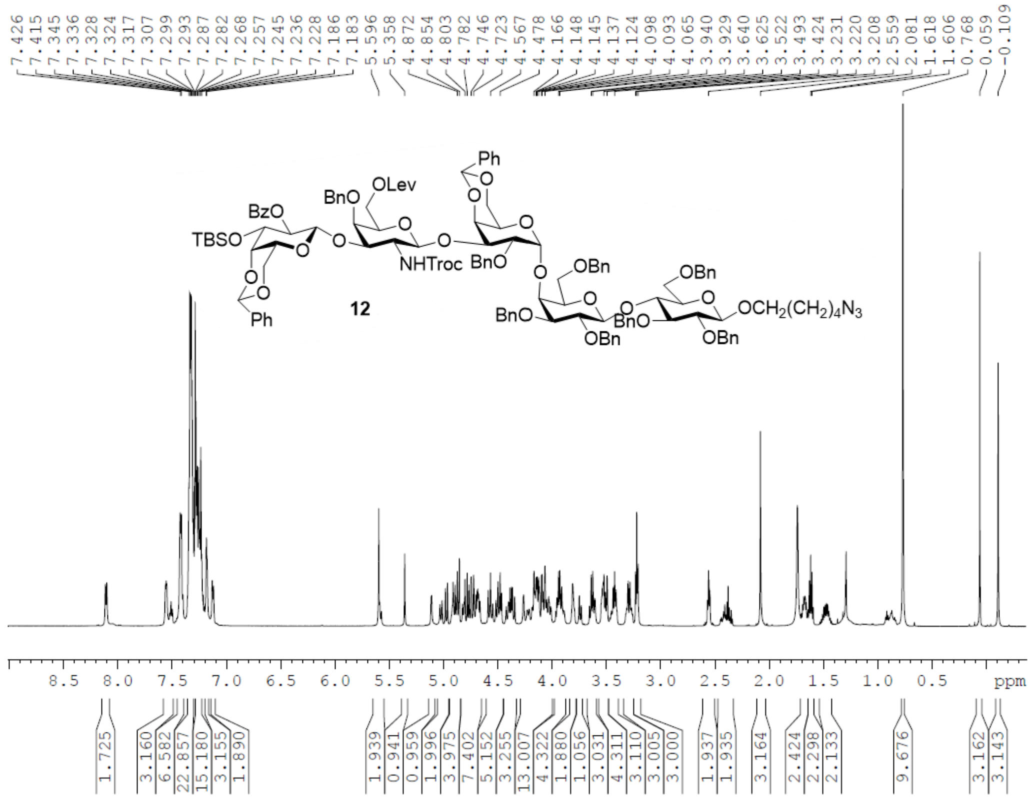

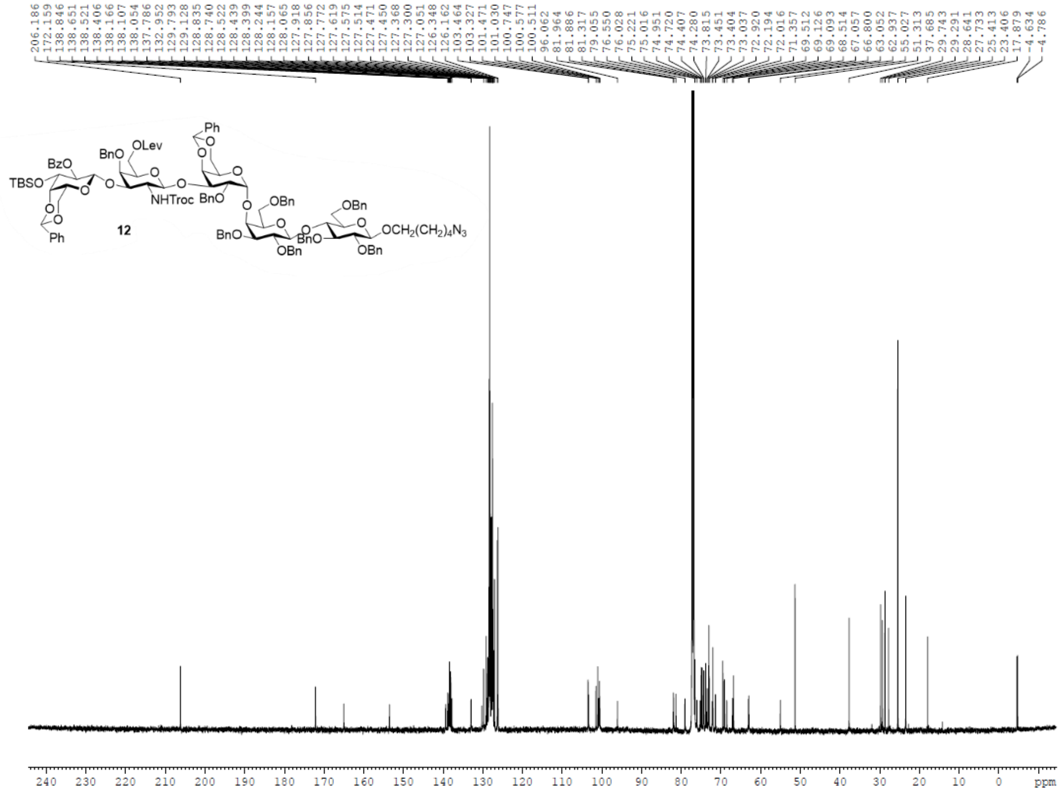

Compound 13

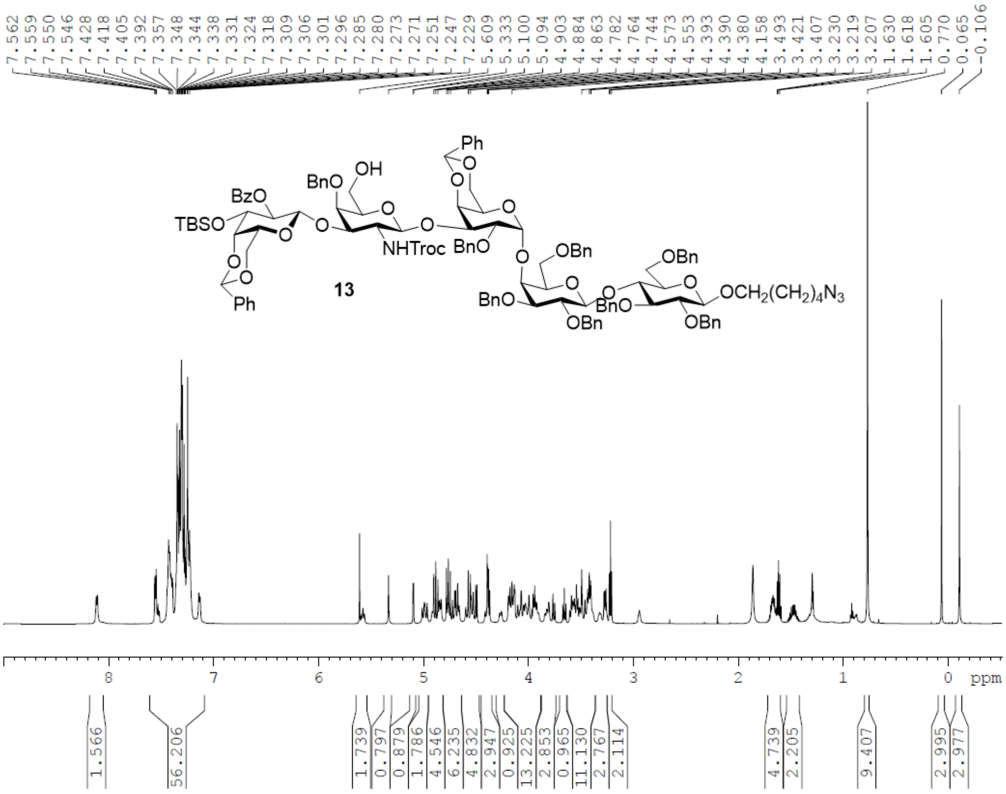
TBSO
BzO
BnO
OH
Ph
NHTroc
BnO
OBn
OBn
BnO
BnO
OBn
OBn
OCH2(CH2)4N3
13
ppm

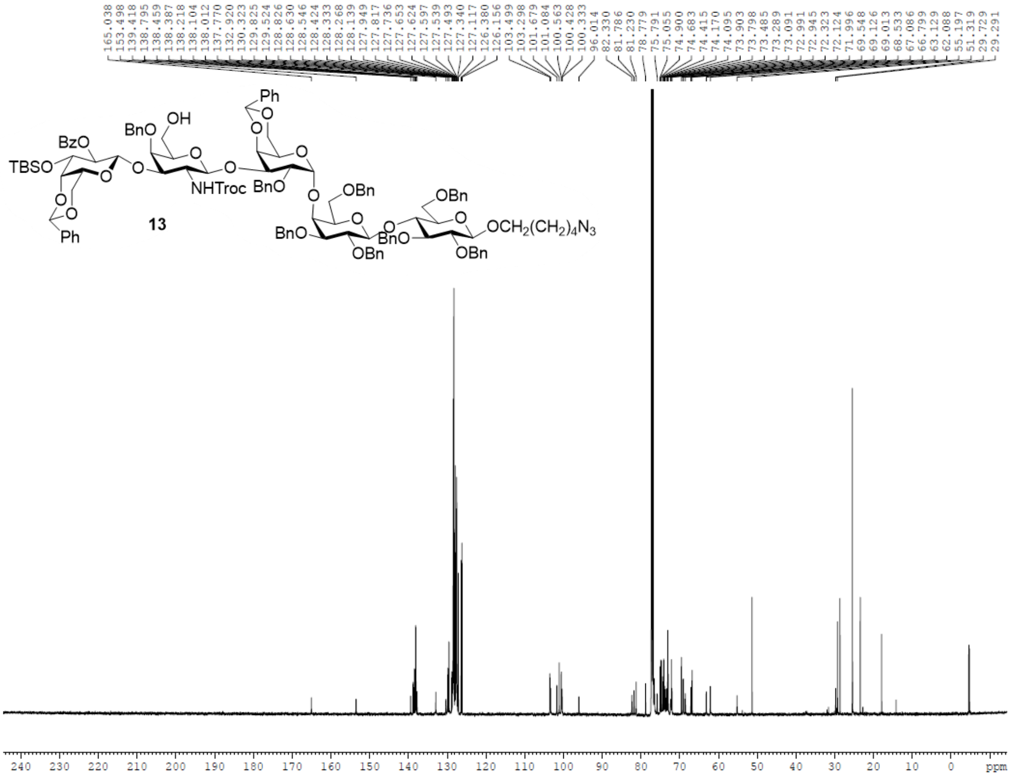
TBSO
BzO
BnO
OH
Ph
NHTroc
BnO
OBn
OBn
BnO
BnO
OBn
OBn
OCH2(CH2)4N3
13
ppm

Compound 14

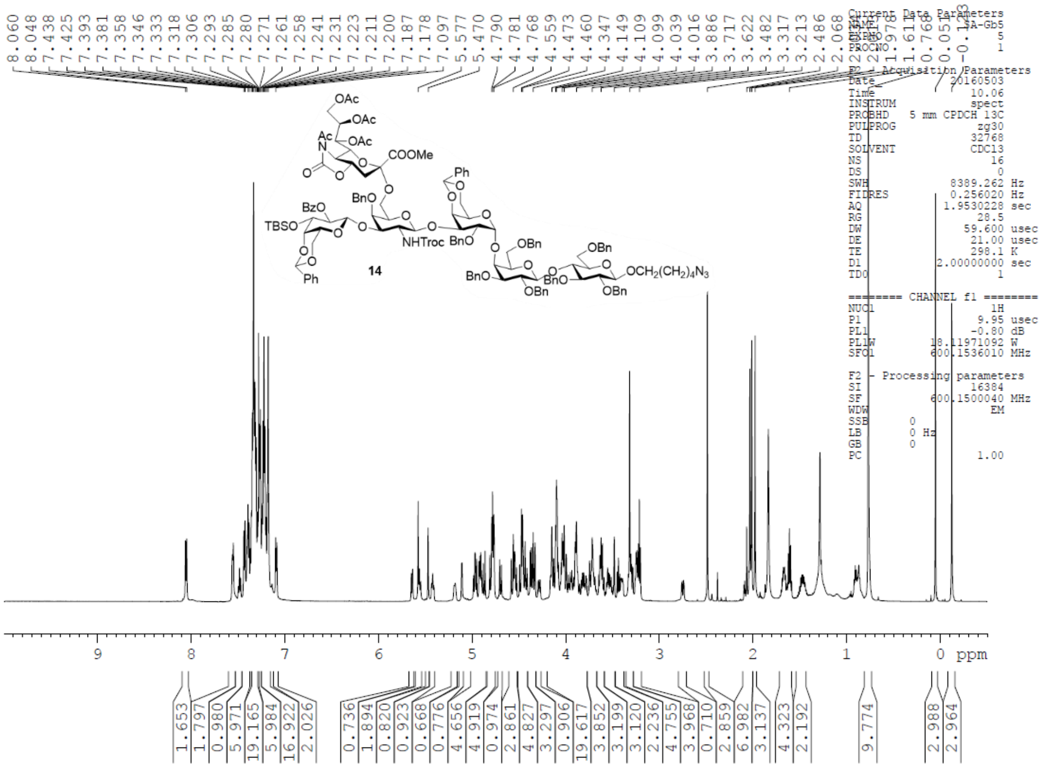

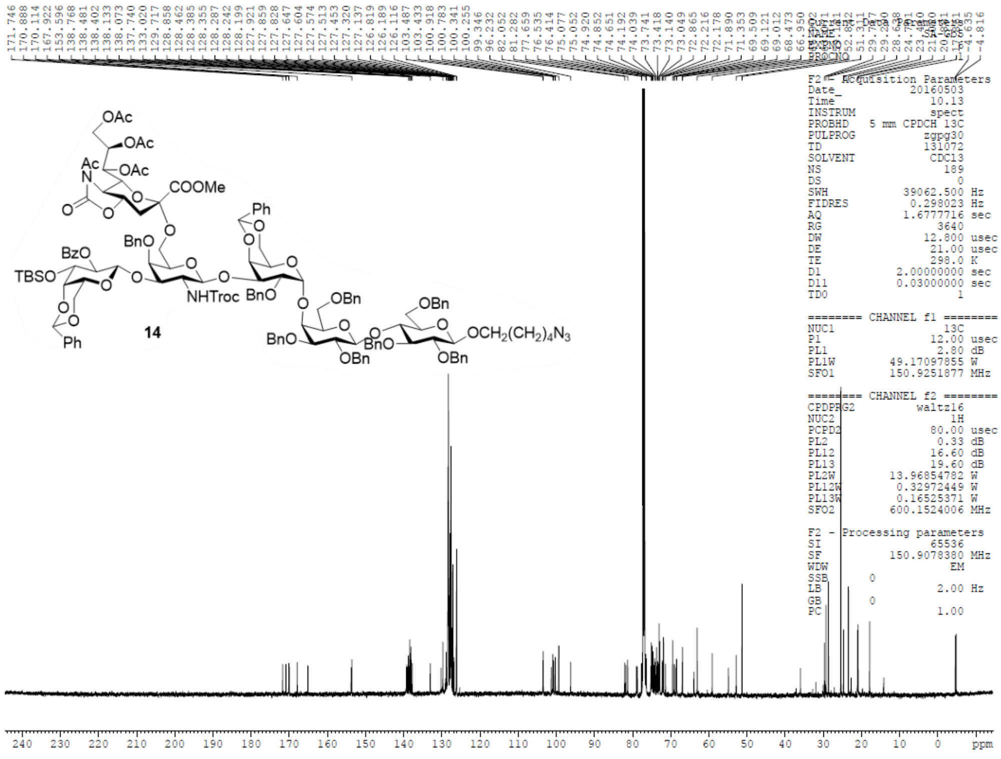

Compound 3

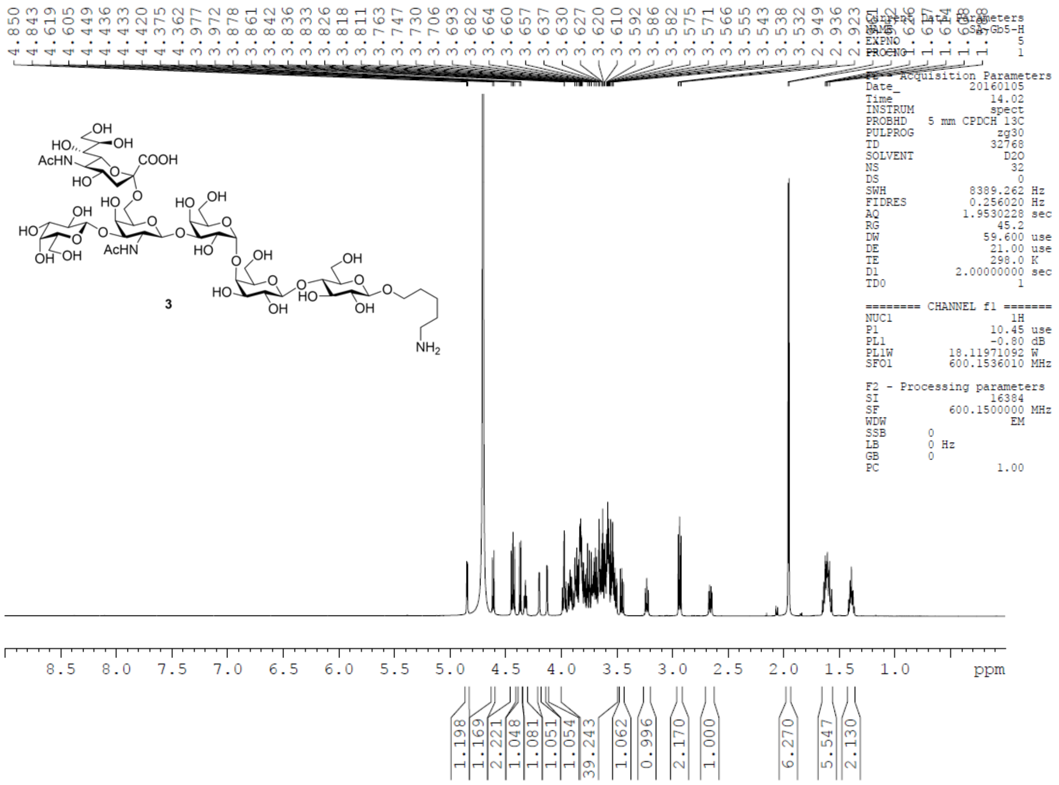
4.850
4.843
4.619
4.605
4.449
4.436
4.433
4.420
4.375
4.362
3.977
3.972
3.878
3.861
3.842
3.836
3.833
3.826
3.818
3.811
3.763
3.747
3.730
3.706
3.693
3.682
3.664
3.660
3.657
3.637
3.630
3.627
3.620
3.610
3.592
3.586
3.582
3.575
3.571
3.566
3.555
3.543
3.538
3.532
2.949
2.936
2.923
Current Data Parameters
NAME SA-Gb5-H
EXPNO 5
PROCNO 1
F2 - Acquisition Parameters
Date_ 20160105
Time 14.02
INSTRUM spect
PROBHD 5 mm CPDCH 13C
PULPROG zg30
TD 32768
SOLVENT D2O
NS 32
DS 0
SWH 8389.262 Hz
FIDRES 0.256020 Hz
AQ 1.9530228 sec
RG 45.2
DW 59.600 use
DE 21.00 use
TE 298.0 K
D1 2.00000000 sec
TD0 1
======== CHANNEL f1 ========
NUC1 1H
P1 10.45 use
PL1 -0.80 dB
PL1W 18.11971092 W
SFO1 600.1536010 MHz
F2 - Processing parameters
SI 16384
SF 600.1500000 MHz
WDW EM
SSB 0
LB 0 Hz
GB 0
PC 1.00
HO
OH
AcHN
COOH
AcHN
NH2
3
8.5 8.0 7.5 7.0 6.5 6.0 5.5 5.0 4.5 4.0 3.5 3.0 2.5 2.0 1.5 1.0 ppm
1.198
1.169
2.221
1.048
1.081
1.051
1.054
39.243
1.062
0.996
2.170
1.000
6.270
5.547
2.130

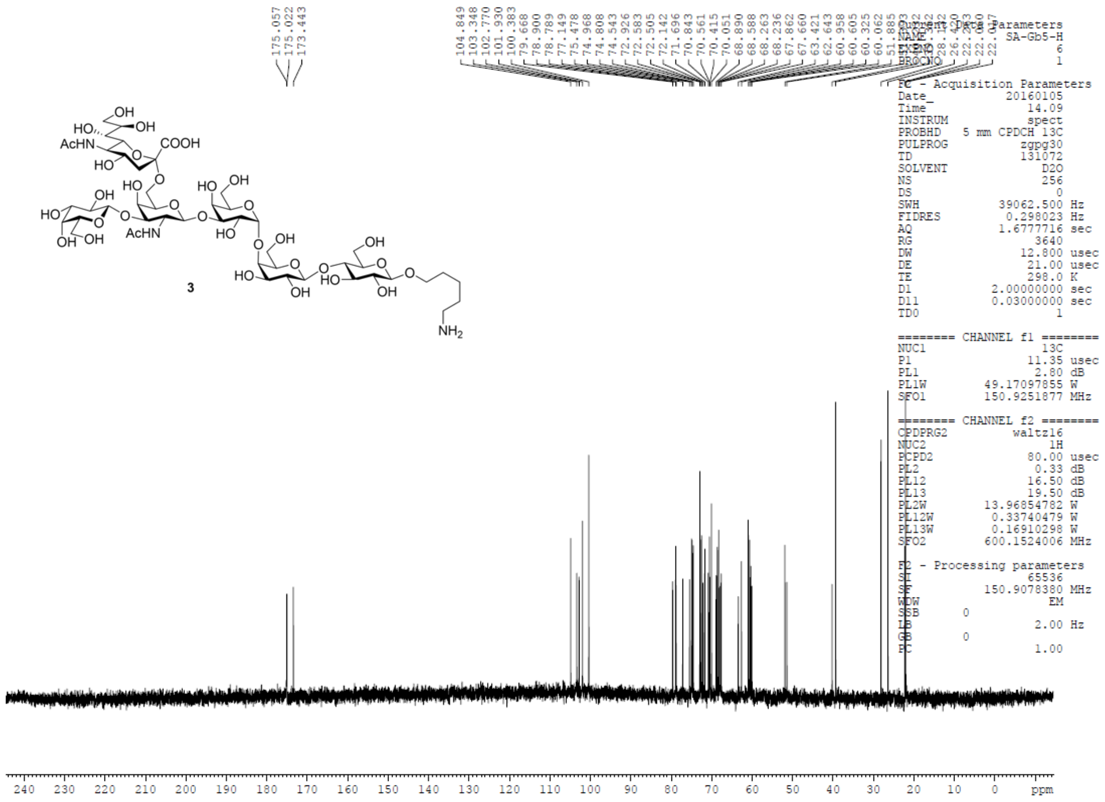
175.057
175.022
173.443
104.849
103.348
102.770
101.930
100.383
79.668
78.900
78.789
77.149
75.478
74.968
74.808
74.543
72.926
72.583
72.505
72.142
71.696
70.843
70.561
70.415
70.051
68.890
68.588
68.263
68.236
67.862
67.660
63.421
62.643
60.958
60.605
60.325
60.062
51.885
Current Data Parameters
NAME SA-Gb5-H
EXPNO 6
PROCNO 1
F2 - Acquisition Parameters
Date_ 20160105
Time 14.09
INSTRUM spect
PROBHD 5 mm CPDCH 13C
PULPROG zgpg30
TD 131072
SOLVENT D2O
NS 256
DS 0
SWH 39062.500 Hz
FIDRES 0.298023 Hz
AQ 1.6777716 sec
RG 3640
DW 12.800 usec
DE 21.00 usec
TE 298.0 K
D1 2.00000000 sec
D11 0.03000000 sec
TD0 1
======== CHANNEL f1 ========
NUC1 13C
P1 11.35 usec
PL1 2.80 dB
PL1W 49.17097855 W
SFO1 150.9251877 MHz
======== CHANNEL f2 ========
CPDPRG2 waltz16
NUC2 1H
PCPD2 80.00 usec
PL2 0.33 dB
PL12 16.50 dB
PL13 19.50 dB
PL2W 13.96854782 W
PL12W 0.33740479 W
PL13W 0.16910298 W
SFO2 600.1524006 MHz
F2 - Processing parameters
SI 65536
SF 150.9078380 MHz
WDW EM
SSB 0
LB 2.00 Hz
GB 0
PC 1.00
COOH
AcHN
NH2
3
240 230 220 210 200 190 180 170 160 150 140 130 120 110 100 90 80 70 60 50 40 30 20 10 0 ppm

Compound 4a

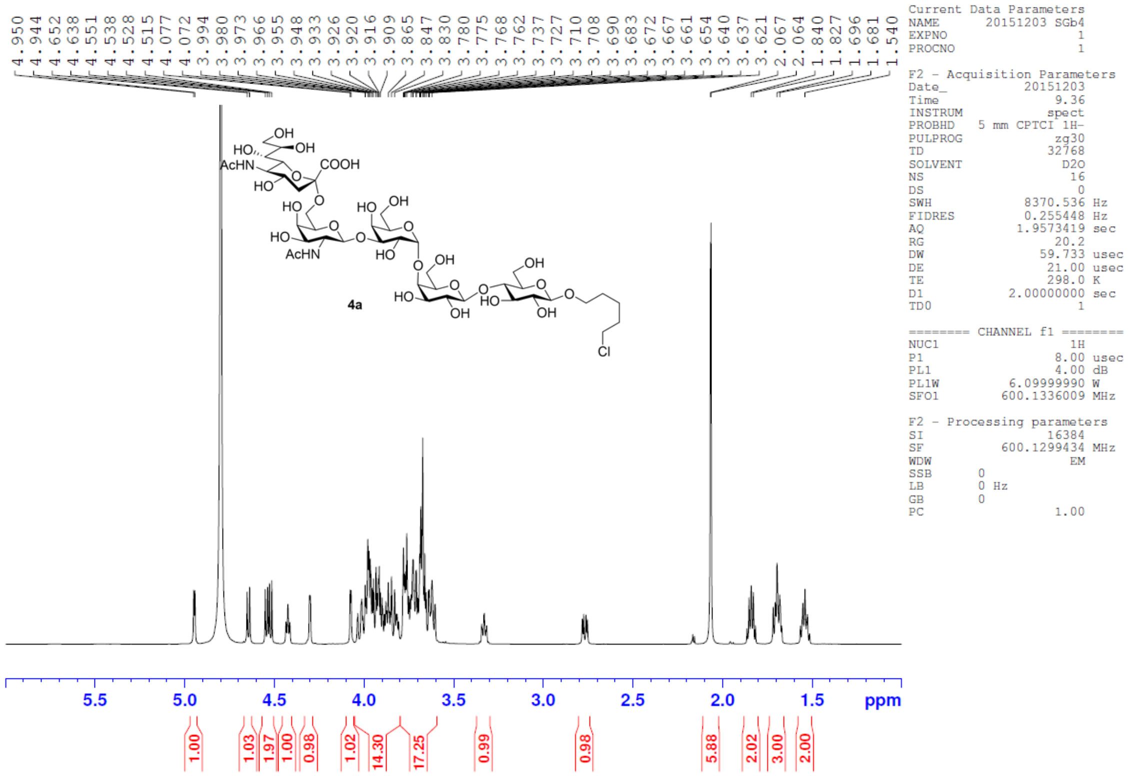
4.950 4.944 4.652 4.638 4.551 4.538 4.528 4.515 4.077 4.072 3.994 3.980 3.973 3.966 3.955 3.948 3.933 3.926 3.920 3.916 3.909 3.865 3.847 3.830 3.780 3.775 3.768 3.762 3.737 3.727 3.710 3.708 3.690 3.683 3.672 3.667 3.661 3.654 3.640 3.637 3.621 2.067 2.064 1.840 1.827 1.696 1.681 1.540
OH
HO
OH
AcHN
COOH
O
HO
O
HO
HO
OH
O
O
HO
O
AcHN
HO
O
OH
O
OH
O
HO
O
HO
O
OH
OH
4a
Cl
Current Data Parameters
NAME 20151203 SGb4
EXPNO 1
PROCNO 1
F2 - Acquisition Parameters
Date_ 20151203
Time 9.36
INSTRUM spect
PROBHD 5 mm CPTCI 1H-
PULPROG zg30
TD 32768
SOLVENT D2O
NS 16
DS 0
SWH 8370.536 Hz
FIDRES 0.255448 Hz
AQ 1.9573419 sec
RG 20.2
DW 59.733 usec
DE 21.00 usec
TE 298.0 K
D1 2.00000000 sec
TD0 1
======== CHANNEL f1 ========
NUC1 1H
P1 8.00 usec
PL1 4.00 dB
PL1W 6.09999990 W
SFO1 600.1336009 MHz
F2 - Processing parameters
SI 16384
SF 600.1299434 MHz
WDW EM
SSB 0
LB 0 Hz
GB 0
PC 1.00
5.5 5.0 4.5 4.0 3.5 3.0 2.5 2.0 1.5 ppm
1.00 1.03 1.97 1.00 0.98 1.02 14.30 17.25 0.99 0.98 5.88 2.02 3.00 2.00

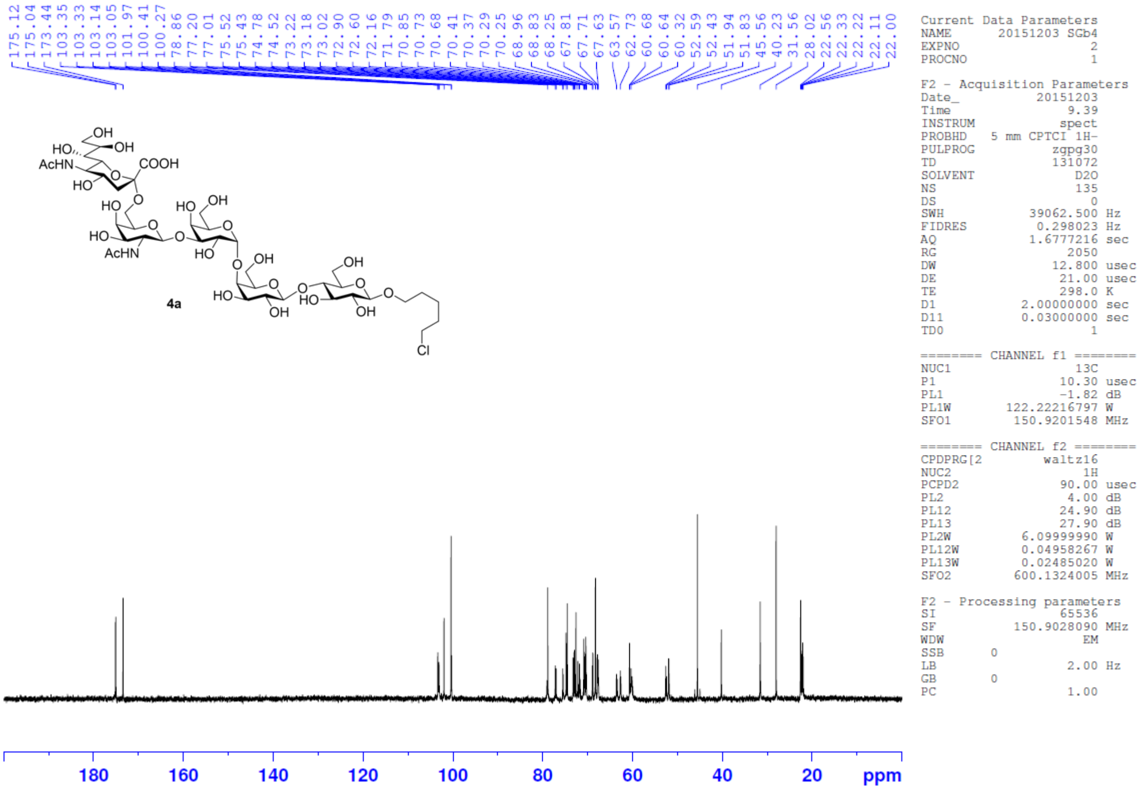
175.12 175.04 173.44 103.35 103.33 103.14 103.05 101.97 100.41 100.27 78.86 77.20 77.01 75.52 75.43 74.78 74.52 73.22 73.18 73.02 72.90 72.60 72.16 71.79 70.85 70.73 70.68 70.41 70.37 70.29 70.25 68.96 68.83 68.25 67.81 67.71 67.63 63.57 62.73 60.68 60.64 60.32 52.59 52.43 51.94 51.83 45.56 40.23 31.56 28.02 22.56 22.33 22.22 22.11 22.00
4a
Cl
Current Data Parameters
NAME 20151203 SGb4
EXPNO 2
PROCNO 1
F2 - Acquisition Parameters
Date_ 20151203
Time 9.39
INSTRUM spect
PROBHD 5 mm CPTCI 1H-
PULPROG zgpg30
TD 131072
SOLVENT D2O
NS 135
DS 0
SWH 39062.500 Hz
FIDRES 0.298023 Hz
AQ 1.6777216 sec
RG 2050
DW 12.800 usec
DE 21.00 usec
TE 298.0 K
D1 2.00000000 sec
D11 0.03000000 sec
TD0 1
======== CHANNEL f1 ========
NUC1 13C
P1 10.30 usec
PL1 -1.82 dB
PL1W 122.22216797 W
SFO1 150.9201548 MHz
======== CHANNEL f2 ========
CPDPRG[2 waltz16
NUC2 1H
PCPD2 90.00 usec
PL2 4.00 dB
PL12 24.90 dB
PL13 27.90 dB
PL2W 6.09999990 W
PL12W 0.04958267 W
PL13W 0.02485020 W
SFO2 600.1324005 MHz
F2 - Processing parameters
SI 65536
SF 150.9028090 MHz
WDW EM
SSB 0
LB 2.00 Hz
GB 0
PC 1.00
180 160 140 120 100 80 60 40 20 ppm

Compound 5a

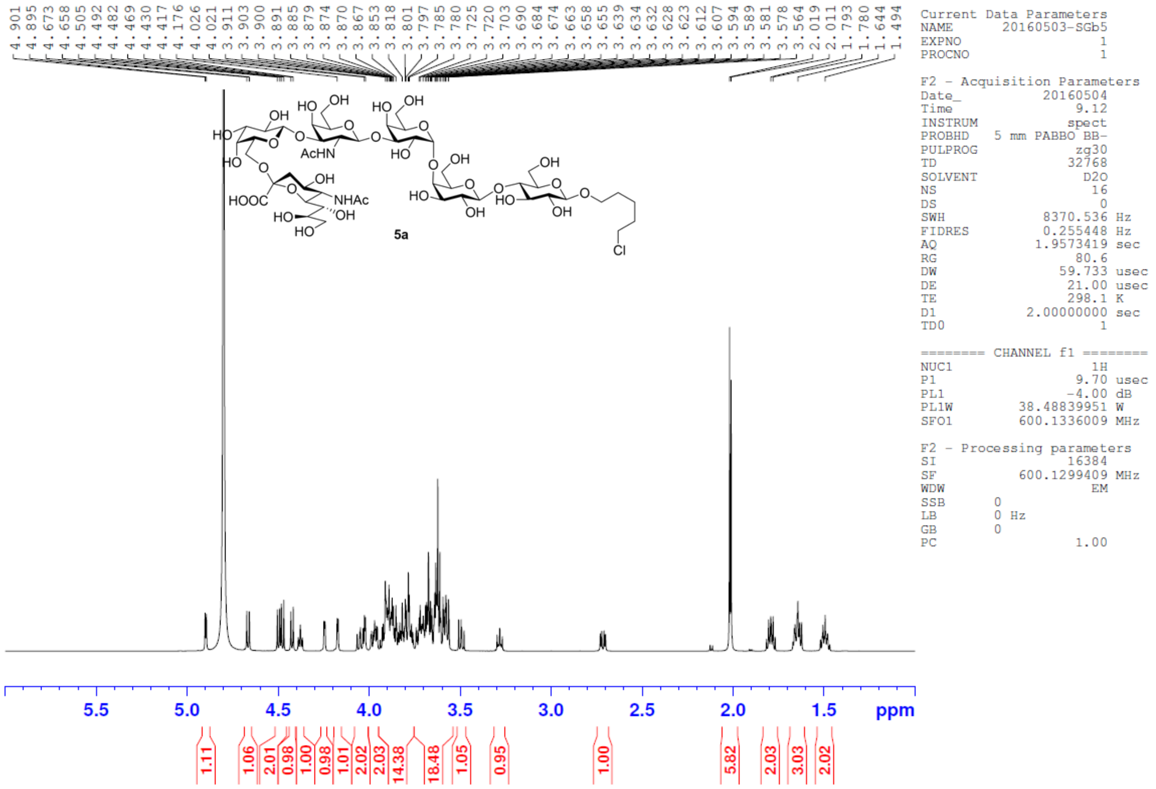
4.901 4.895 4.673 4.658 4.505 4.492 4.482 4.469 4.430 4.417 4.176 4.026 4.021 3.911 3.903 3.900 3.891 3.885 3.879 3.874 3.870 3.867 3.853 3.818 3.801 3.797 3.785 3.780 3.725 3.720 3.703 3.690 3.684 3.674 3.663 3.658 3.655 3.639 3.634 3.632 3.628 3.623 3.612 3.607 3.594 3.589 3.581 3.578 3.564 2.019 2.011 1.793 1.780 1.644 1.494
5a
Current Data Parameters
NAME 20160503-SGb5
EXPNO 1
PROCNO 1
F2 - Acquisition Parameters
Date_ 20160504
Time 9.12
INSTRUM spect
PROBHD 5 mm PABBO BB-
PULPROG zg30
TD 32768
SOLVENT D2O
NS 16
DS 0
SWH 8370.536 Hz
FIDRES 0.255448 Hz
AQ 1.9573419 sec
RG 80.6
DW 59.733 usec
DE 21.00 usec
TE 298.1 K
D1 2.00000000 sec
TD0 1
======== CHANNEL f1 ========
NUC1 1H
P1 9.70 usec
PL1 -4.00 dB
PL1W 38.48839951 W
SFO1 600.1336009 MHz
F2 - Processing parameters
SI 16384
SF 600.1299409 MHz
WDW EM
SSB 0
LB 0 Hz
GB 0
PC 1.00
5.5 5.0 4.5 4.0 3.5 3.0 2.5 2.0 1.5 ppm
1.11 1.06 2.01 0.98 1.00 0.98 1.01 2.02 2.03 14.38 18.48 1.05 0.95 1.00 5.82 2.03 3.03 2.02

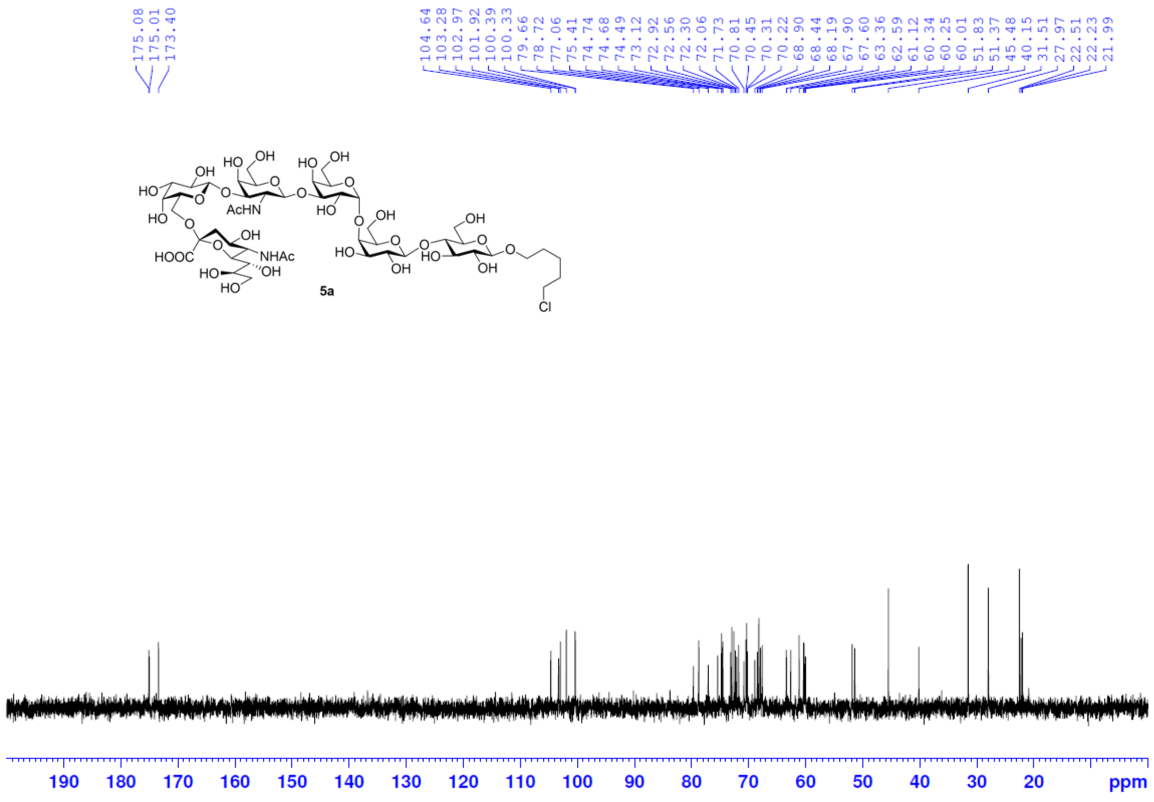
175.08 175.01 173.40
104.64 103.28 102.97 101.92 100.39 100.33 79.66 78.72 77.06 75.41 74.74 74.68 74.49 73.12 72.92 72.56 72.30 72.06 71.73 70.81 70.45 70.31 70.22 68.90 68.44 68.19 67.90 67.60 63.36 62.59 61.12 60.34 60.25 60.01 51.83 51.37 45.48 40.15 31.51 27.97 22.51 22.23 21.99
5a
190 180 170 160 150 140 130 120 110 100 90 80 70 60 50 40 30 20 ppm

Compound 16

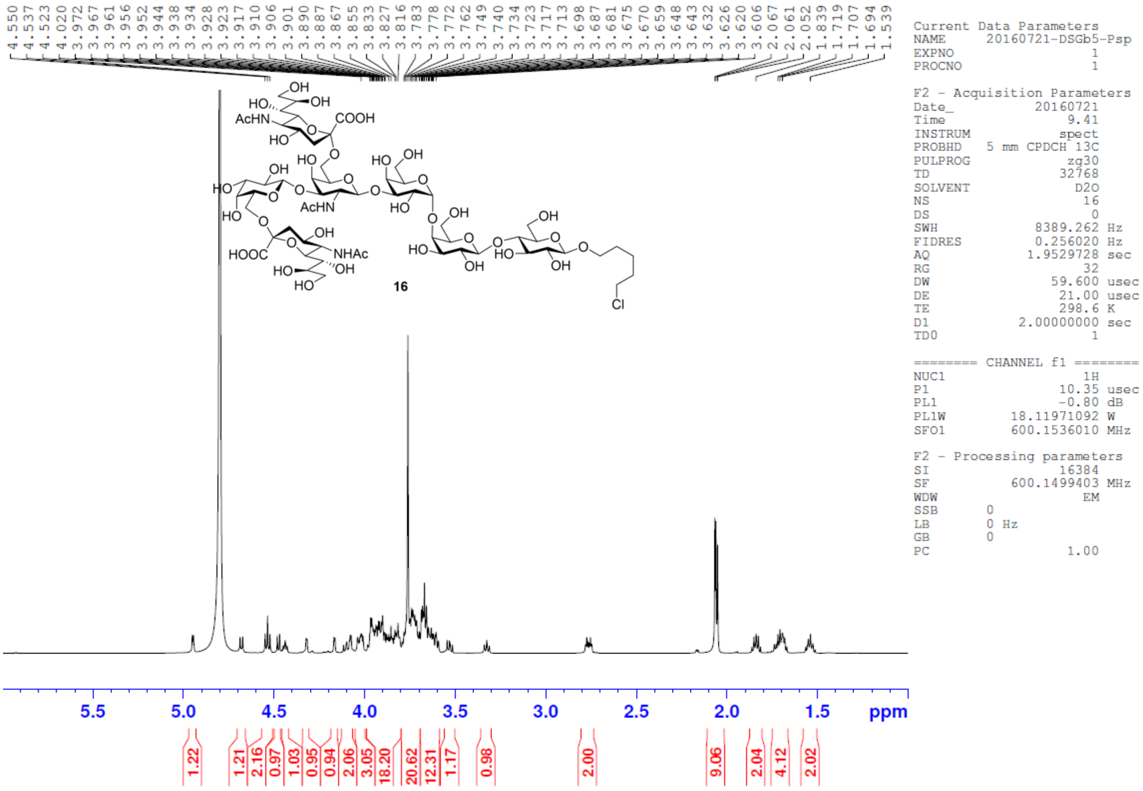

4.550 4.537 4.523 4.020 3.972 3.967 3.961 3.956 3.952 3.944 3.938 3.934 3.928 3.923 3.917 3.910 3.906 3.901 3.890 3.887 3.867 3.855 3.833 3.827 3.816 3.783 3.778 3.772 3.762 3.749 3.740 3.734 3.723 3.717 3.713 3.698 3.687 3.681 3.675 3.670 3.659 3.648 3.643 3.632 3.626 3.620 3.606 2.067 2.061 2.052 1.839 1.719 1.707 1.694 1.539
16
Current Data Parameters
NAME 20160721-DSGb5-Psp
EXPNO 1
PROCNO 1
F2 - Acquisition Parameters
Date_ 20160721
Time 9.41
INSTRUM spect
PROBHD 5 mm CPDCH 13C
PULPROG zg30
TD 32768
SOLVENT D2O
NS 16
DS 0
SWH 8389.262 Hz
FIDRES 0.256020 Hz
AQ 1.9529728 sec
RG 32
DW 59.600 usec
DE 21.00 usec
TE 298.6 K
D1 2.00000000 sec
TD0 1
======== CHANNEL f1 ========
NUC1 1H
P1 10.35 usec
PL1 -0.80 dB
PL1W 18.11971092 W
SFO1 600.1536010 MHz
F2 - Processing parameters
SI 16384
SF 600.1499403 MHz
WDW EM
SSB 0
LB 0 Hz
GB 0
PC 1.00
5.5 5.0 4.5 4.0 3.5 3.0 2.5 2.0 1.5 ppm
1.22 1.21 2.16 0.97 1.03 0.95 0.94 2.06 3.05 18.20 20.62 12.31 1.17 0.98 2.00 9.06 2.04 4.12 2.02

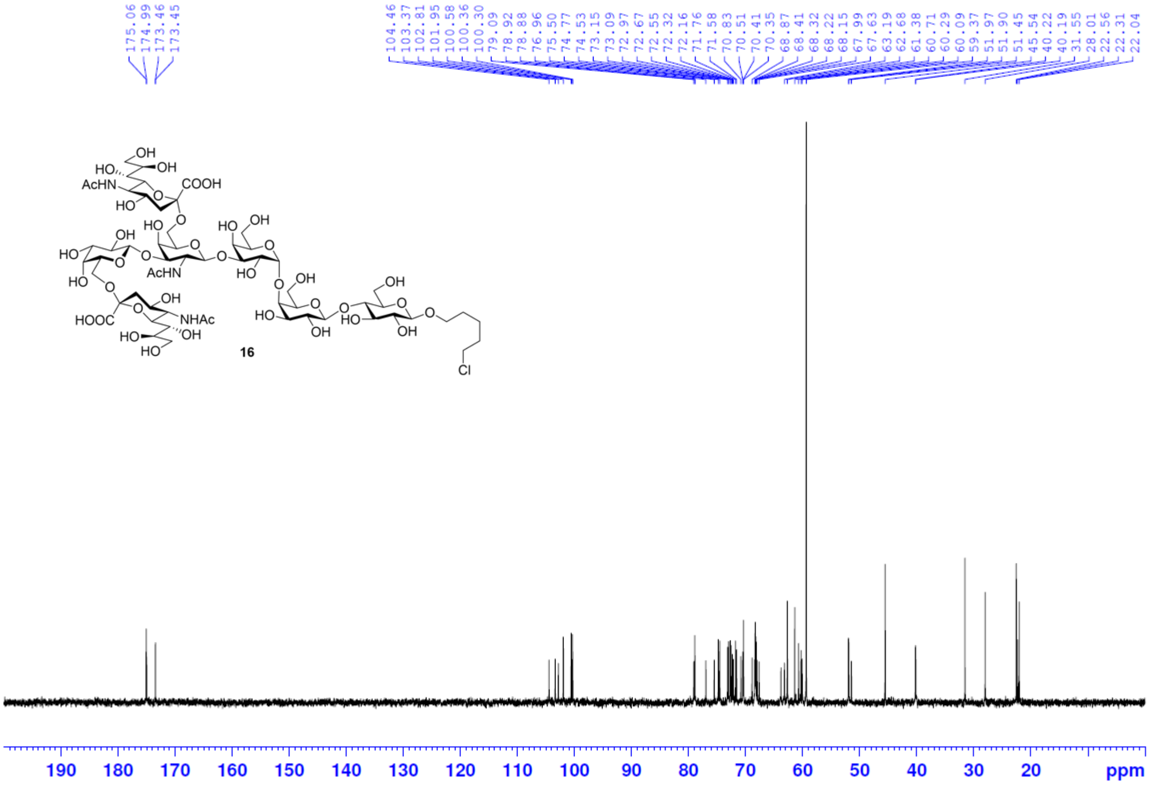

175.06 174.99 173.46 173.45
104.46 103.37 102.81 101.95 100.58 100.36 100.30 79.09 78.92 78.88 76.96 75.50 74.77 74.53 73.15 73.09 72.97 72.67 72.55 72.32 72.16 71.76 71.58 70.83 70.51 70.41 70.35 68.87 68.41 68.32 68.22 68.15 67.99 67.63 63.19 62.68 61.38 60.71 60.29 60.09 59.37 51.97 51.90 51.45 45.54 40.22 40.19 31.55 28.01 22.56 22.31 22.04
16
190 180 170 160 150 140 130 120 110 100 90 80 70 60 50 40 30 20 ppm

Compound 17

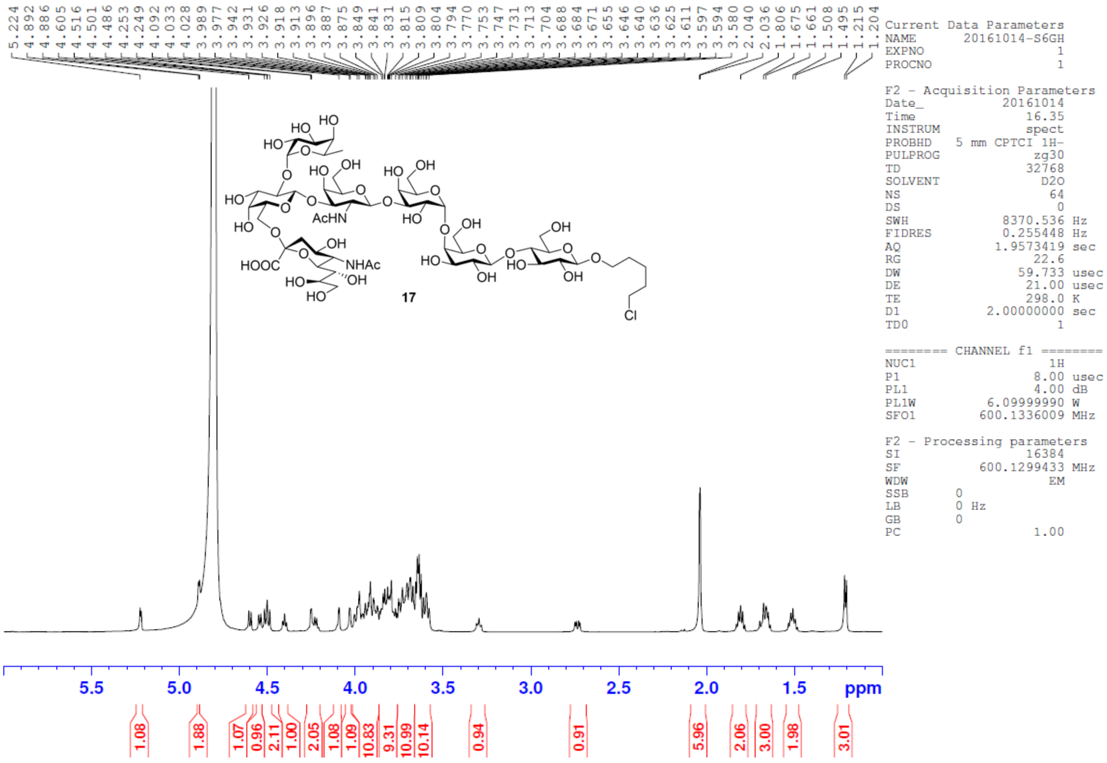
5.224 4.892 4.886 4.605 4.516 4.501 4.486 4.253 4.249 4.092 4.033 4.028 3.989 3.977 3.942 3.931 3.926 3.918 3.913 3.896 3.887 3.875 3.849 3.841 3.831 3.815 3.809 3.804 3.794 3.770 3.753 3.747 3.731 3.713 3.704 3.688 3.684 3.671 3.655 3.646 3.640 3.636 3.625 3.611 3.597 3.594 3.580 2.040 2.036 1.806 1.675 1.661 1.508 1.495 1.215 1.204
Current Data Parameters
NAME 20161014-S6GH
EXPNO 1
PROCNO 1
F2 - Acquisition Parameters
Date_ 20161014
Time 16.35
INSTRUM spect
PROBHD 5 mm CPTCI 1H-
PULPROG zg30
TD 32768
SOLVENT D2O
NS 64
DS 0
SWH 8370.536 Hz
FIDRES 0.255448 Hz
AQ 1.9573419 sec
RG 22.6
DW 59.733 usec
DE 21.00 usec
TE 298.0 K
D1 2.00000000 sec
TD0 1
======== CHANNEL f1 ========
NUC1 1H
P1 8.00 usec
PL1 4.00 dB
PL1W 6.09999990 W
SFO1 600.1336009 MHz
F2 - Processing parameters
SI 16384
SF 600.1299433 MHz
WDW EM
SSB 0
LB 0 Hz
GB 0
PC 1.00
17
5.5 5.0 4.5 4.0 3.5 3.0 2.5 2.0 1.5 ppm
1.08 1.88 1.07 0.96 2.11 1.00 2.05 1.08 1.09 10.83 9.31 10.99 10.14 0.94 0.91 5.96 2.06 3.00 1.98 3.01

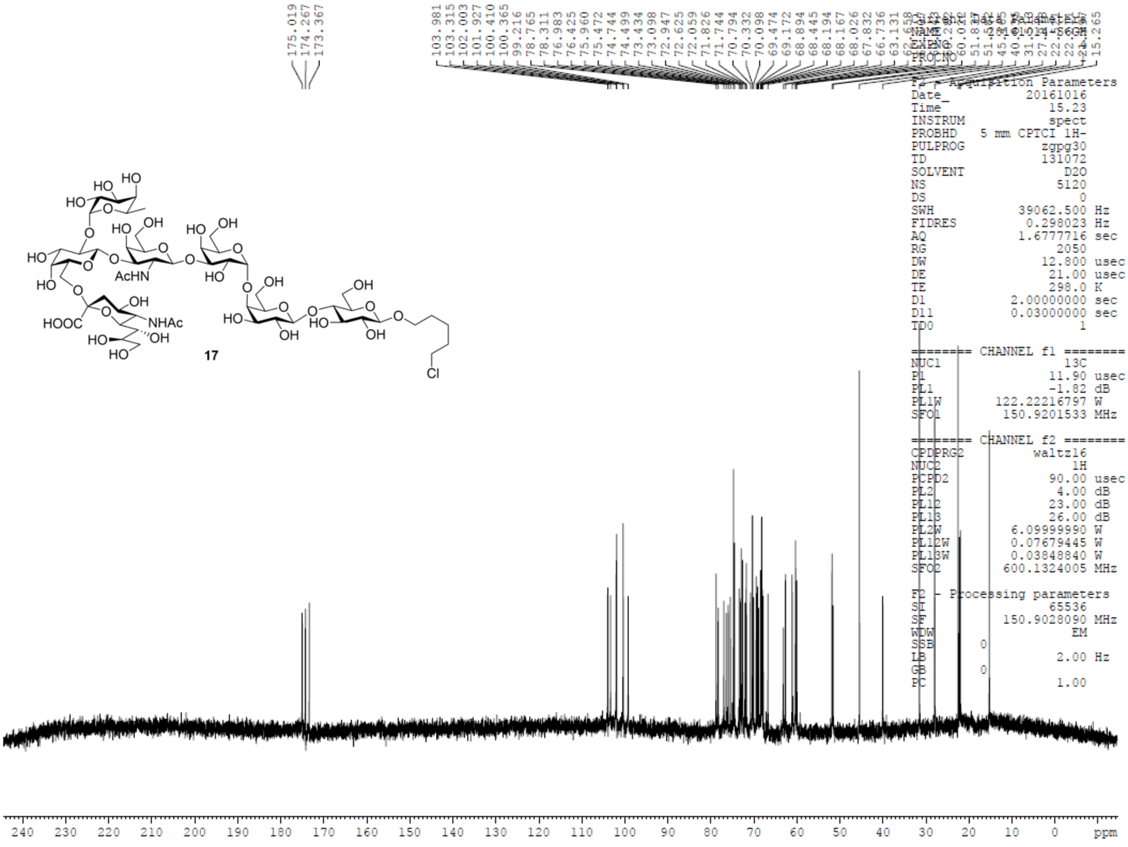
175.019 174.267 173.367
103.981 103.315 102.003 101.927 100.410 100.365 99.216 78.765 78.311 76.983 76.425 75.960 75.472 74.744 74.499 73.434 73.098 72.947 72.625 72.059 71.826 71.744 70.794 70.332 70.098 69.474 69.172 68.894 68.445 68.194 68.167 68.026 67.832 66.736 63.131 15.265
F2 - Acquisition Parameters
Date_ 20161016
Time 15.23
INSTRUM spect
PROBHD 5 mm CPTCI 1H-
PULPROG zgpg30
TD 131072
SOLVENT D2O
NS 5120
DS 0
SWH 39062.500 Hz
FIDRES 0.298023 Hz
AQ 1.6777716 sec
RG 2050
DW 12.800 usec
DE 21.00 usec
TE 298.0 K
D1 2.00000000 sec
D11 0.03000000 sec
TD0 1
======== CHANNEL f1 ========
NUC1 13C
P1 11.90 usec
PL1 -1.82 dB
PL1W 122.22216797 W
SFO1 150.9201533 MHz
======== CHANNEL f2 ========
CPDPRG2 waltz16
NUC2 1H
PCPD2 90.00 usec
PL2 4.00 dB
PL12 23.00 dB
PL13 26.00 dB
PL2W 6.09999990 W
PL12W 0.07679445 W
PL13W 0.03848840 W
SFO2 600.1324005 MHz
F2 - Processing parameters
SI 65536
SF 150.9028090 MHz
WDW EM
SSB 0
LB 2.00 Hz
GB 0
PC 1.00
17
240 230 220 210 200 190 180 170 160 150 140 130 120 110 100 90 80 70 60 50 40 30 20 10 0 ppm

Compound 18

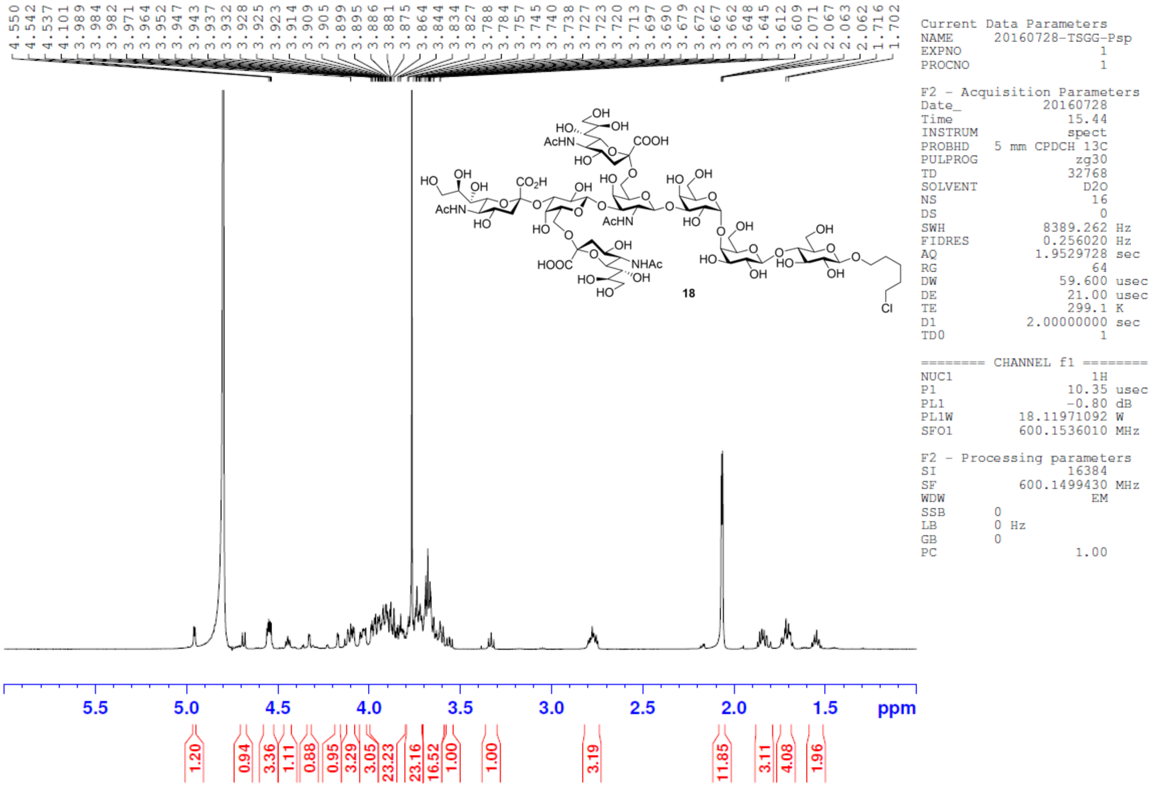
Current Data Parameters
NAME 20160728-TSGG-Psp
EXPNO 1
PROCNO 1
F2 - Acquisition Parameters
Date_ 20160728
Time 15.44
INSTRUM spect
PROBHD 5 mm CPDCH 13C
PULPROG zg30
TD 32768
SOLVENT D2O
NS 16
DS 0
SWH 8389.262 Hz
FIDRES 0.256020 Hz
AQ 1.9529728 sec
RG 64
DW 59.600 usec
DE 21.00 usec
TE 299.1 K
D1 2.00000000 sec
TD0 1
======== CHANNEL f1 ========
NUC1 1H
P1 10.35 usec
PL1 -0.80 dB
PL1W 18.11971092 W
SFO1 600.1536010 MHz
F2 - Processing parameters
SI 16384
SF 600.1499430 MHz
WDW EM
SSB 0
LB 0 Hz
GB 0
PC 1.00
18
ppm

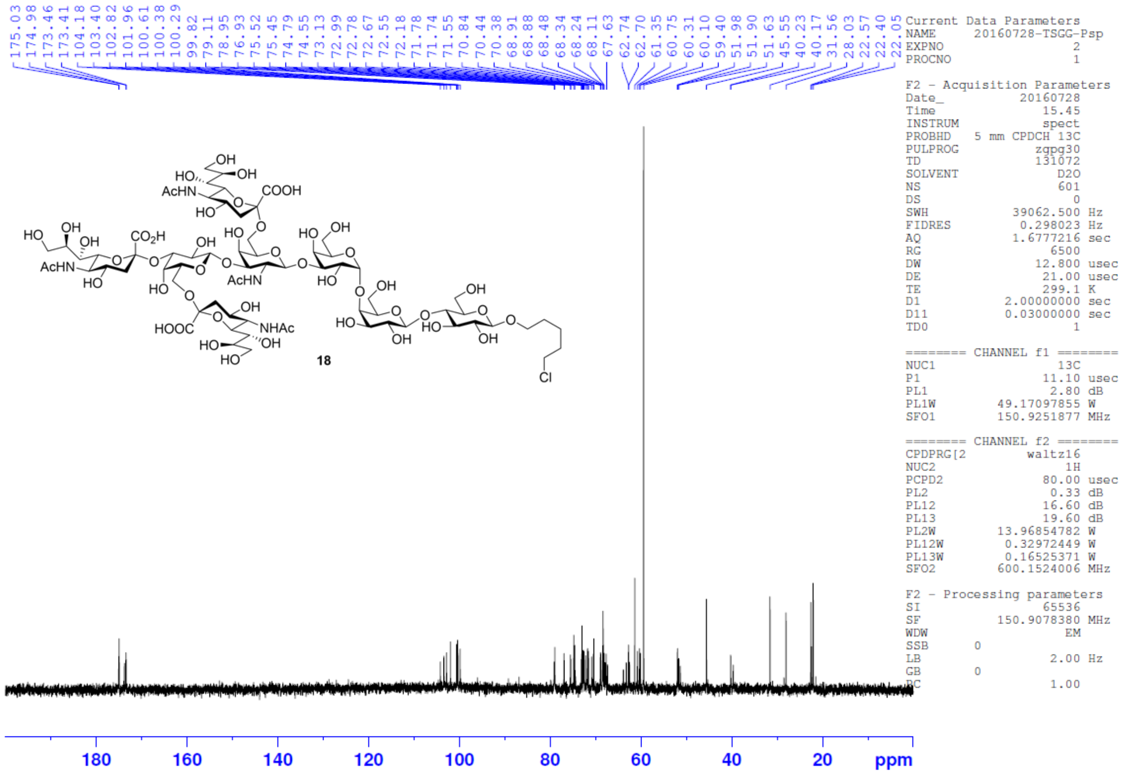
Current Data Parameters
NAME 20160728-TSGG-Psp
EXPNO 2
PROCNO 1
F2 - Acquisition Parameters
Date_ 20160728
Time 15.45
INSTRUM spect
PROBHD 5 mm CPDCH 13C
PULPROG zgpg30
TD 131072
SOLVENT D2O
NS 601
DS 0
SWH 39062.500 Hz
FIDRES 0.298023 Hz
AQ 1.6777216 sec
RG 6500
DW 12.800 usec
DE 21.00 usec
TE 299.1 K
D1 2.00000000 sec
D11 0.03000000 sec
TD0 1
======== CHANNEL f1 ========
NUC1 13C
P1 11.10 usec
PL1 2.80 dB
PL1W 49.17097855 W
SFO1 150.9251877 MHz
======== CHANNEL f2 ========
CPDPRG[2 waltz16
NUC2 1H
PCPD2 80.00 usec
PL2 0.33 dB
PL12 16.60 dB
PL13 19.60 dB
PL2W 13.96854782 W
PL12W 0.32972449 W
PL13W 0.16525371 W
SFO2 600.1524006 MHz
F2 - Processing parameters
SI 65536
SF 150.9078380 MHz
WDW EM
SSB 0
LB 2.00 Hz
GB 0
PC 1.00
18
ppm

DSGb5- NH_2 (Compound 1)

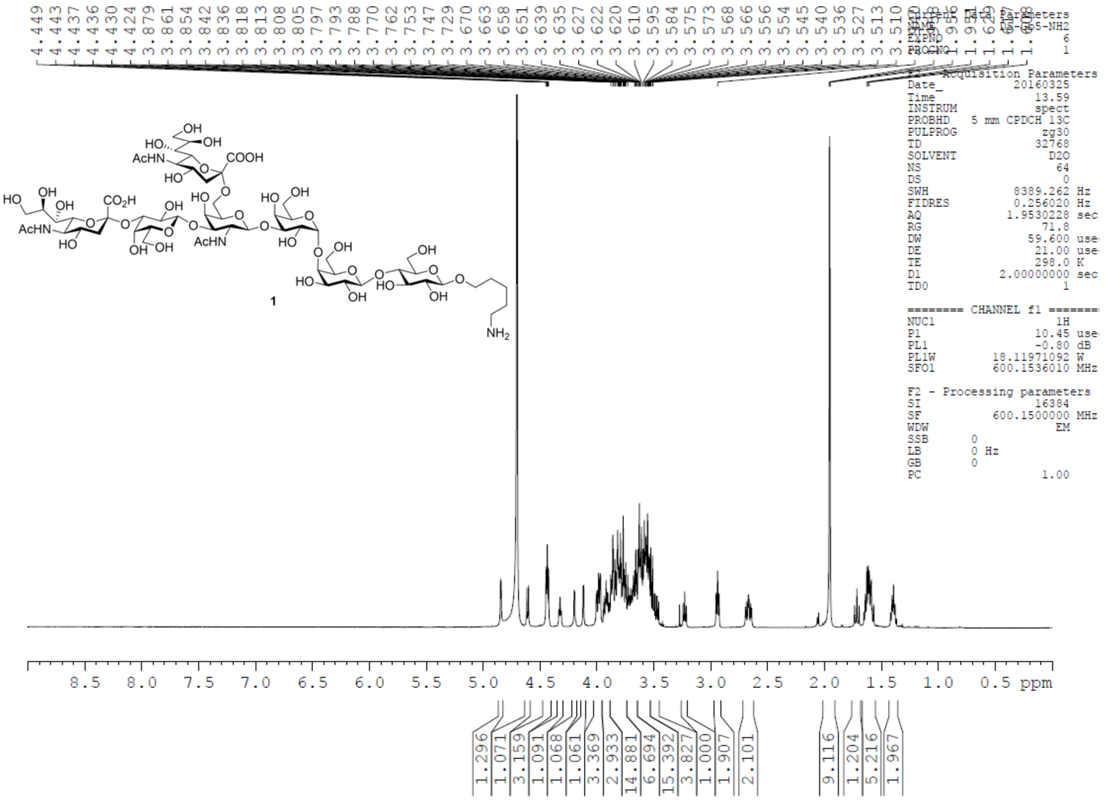

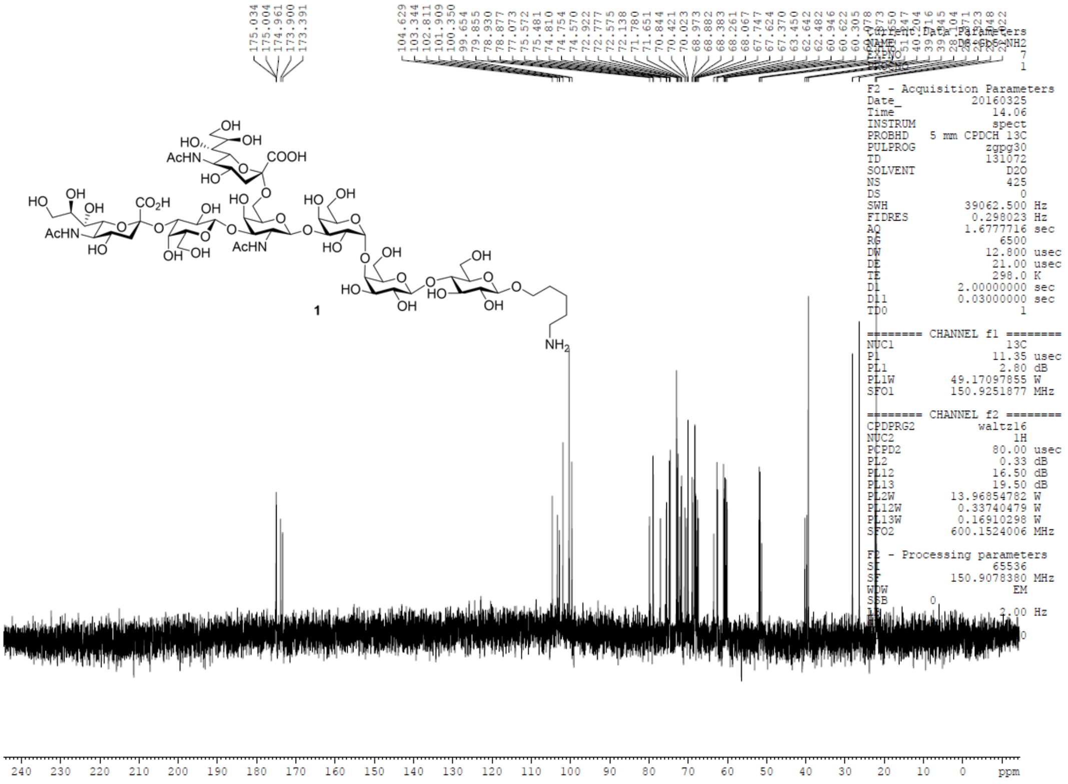

SGb4-NH$_2$ (Compound 4)

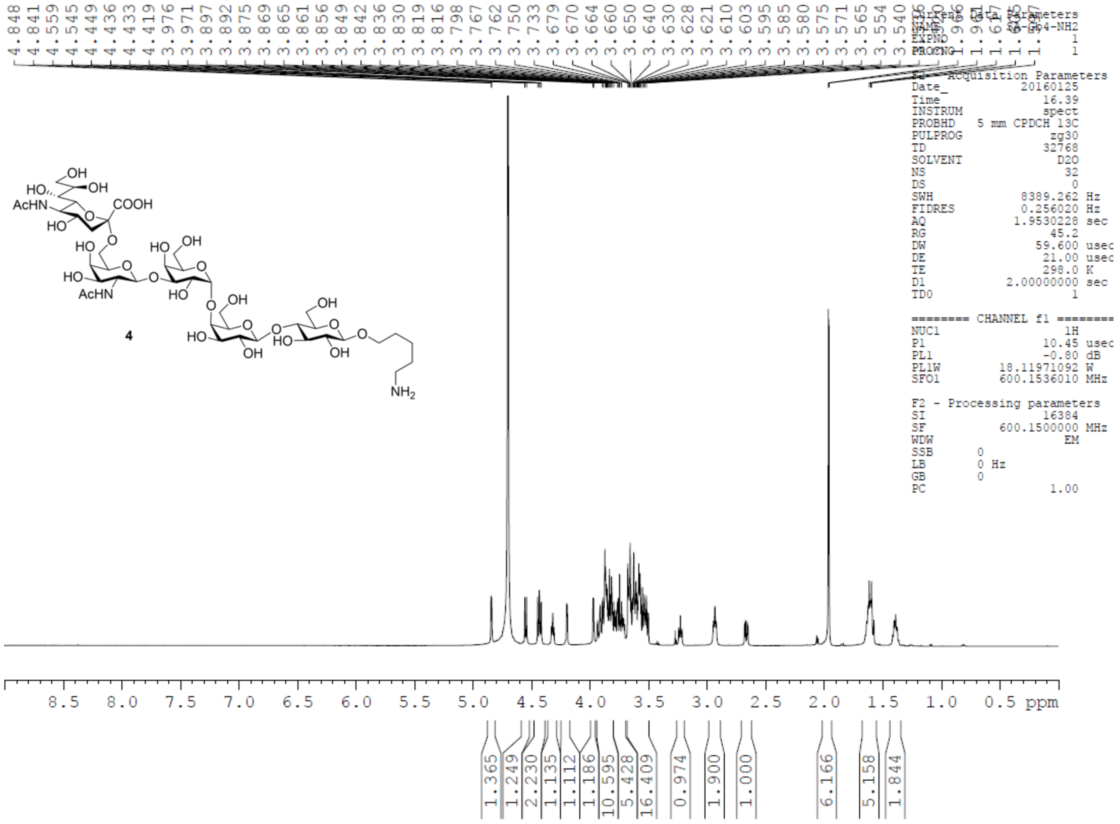

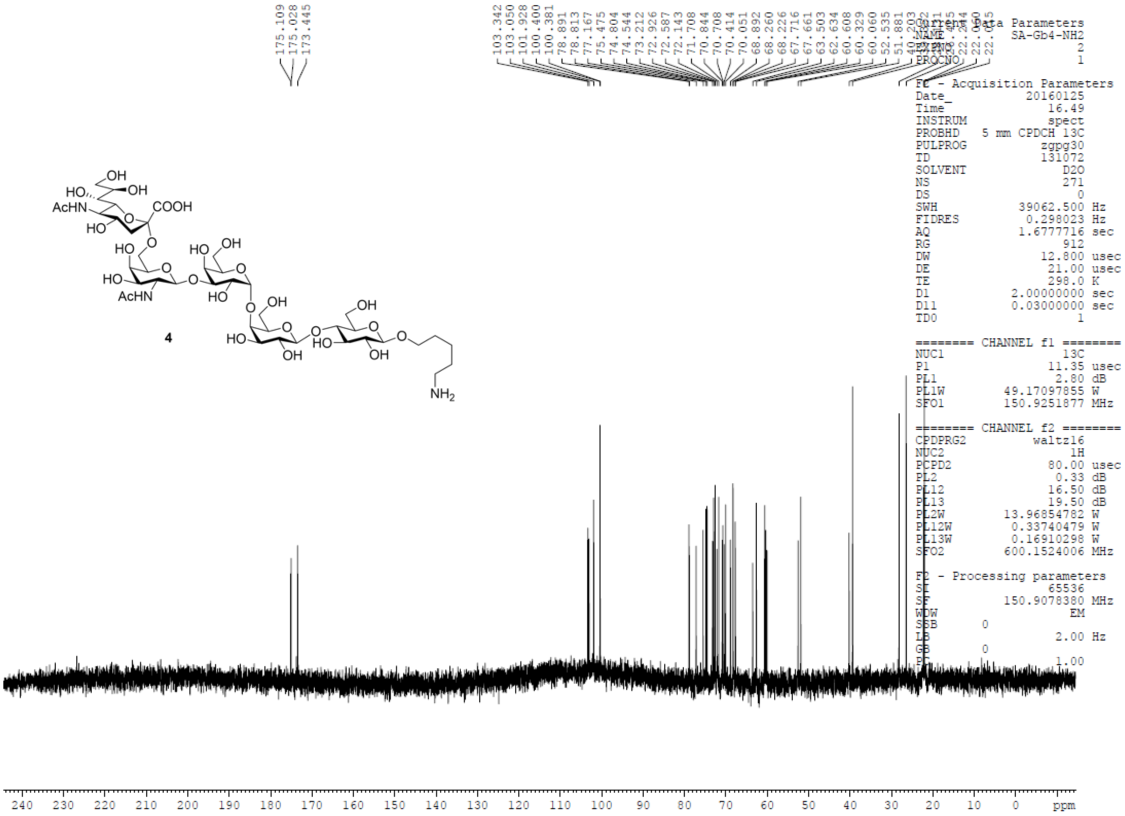

SGb5-NH_2 (Compound 5)

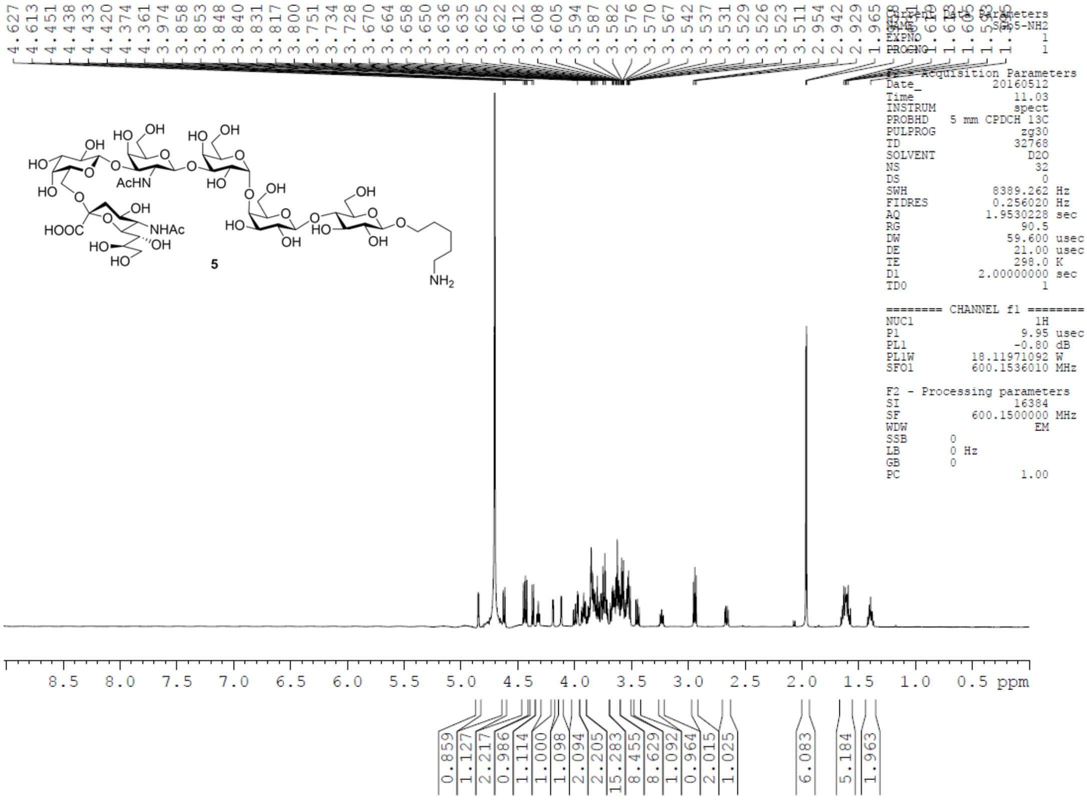

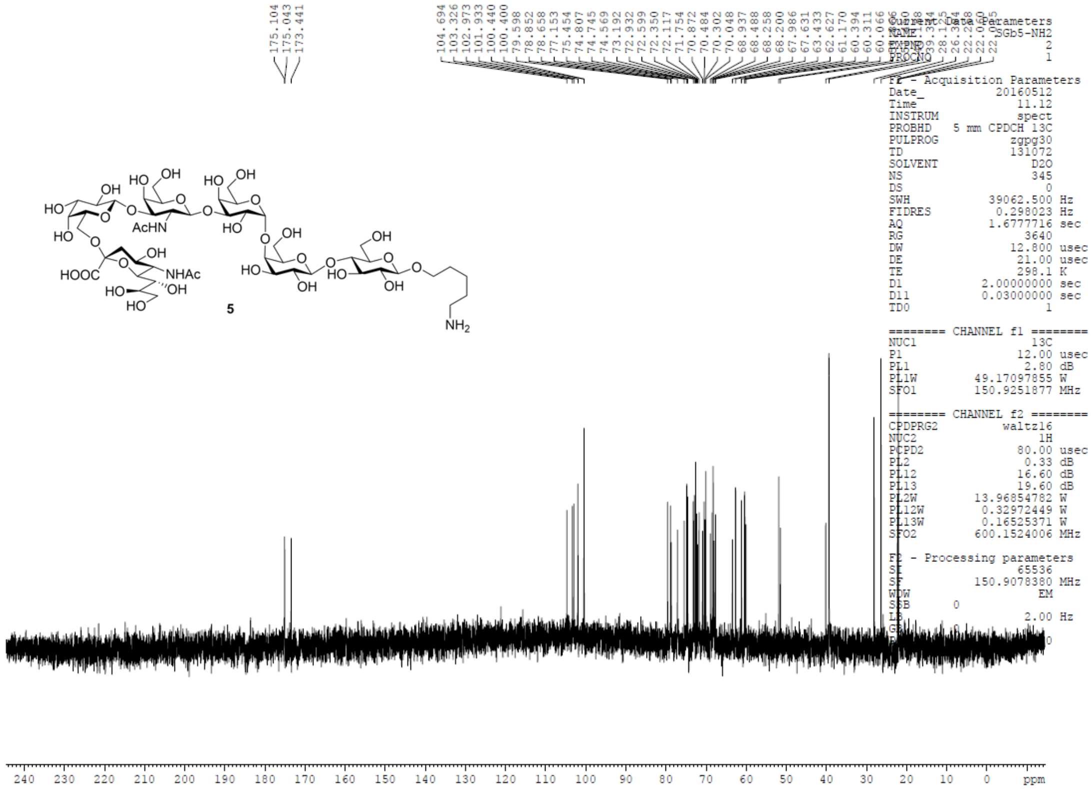

DSGG-NH$_2$ (Compound 2)

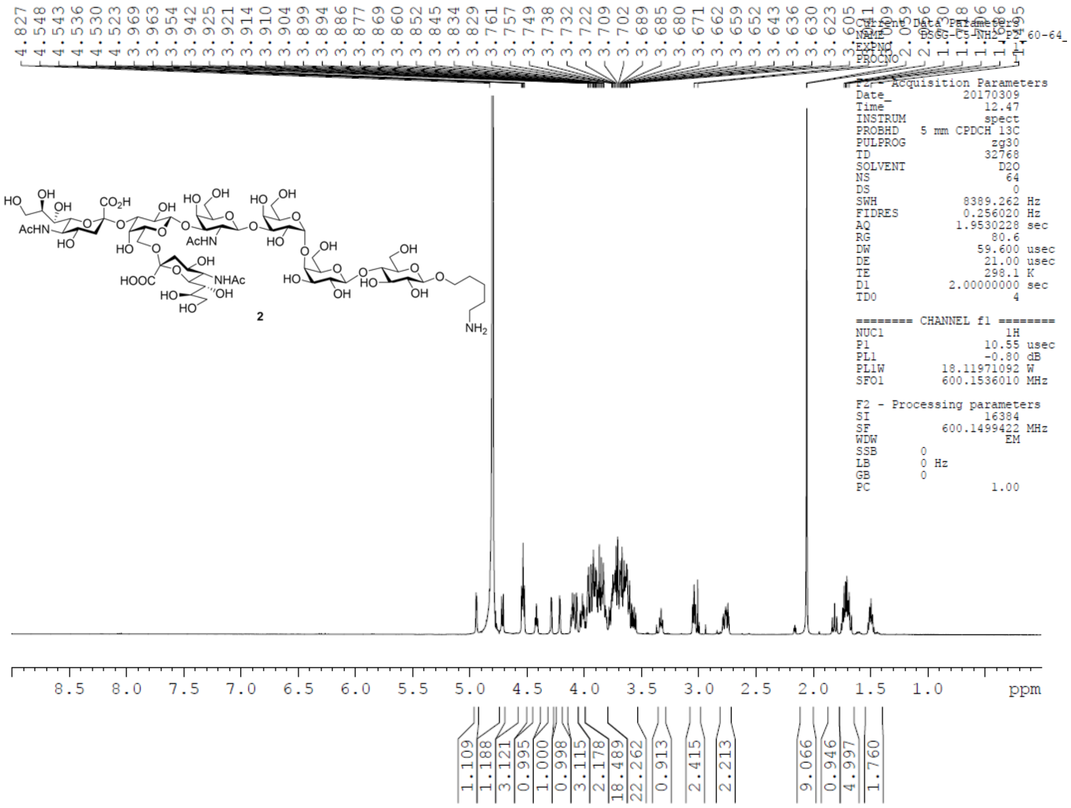

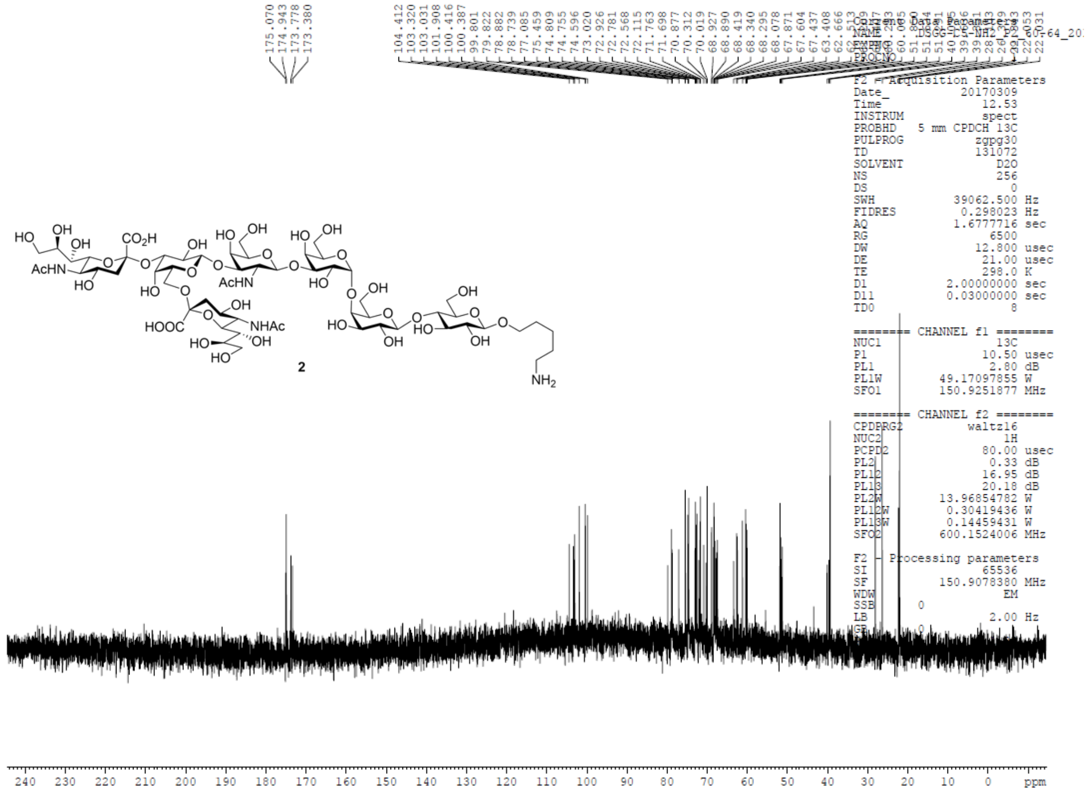

Neu5Gc-pentasaccharide (SL-1)

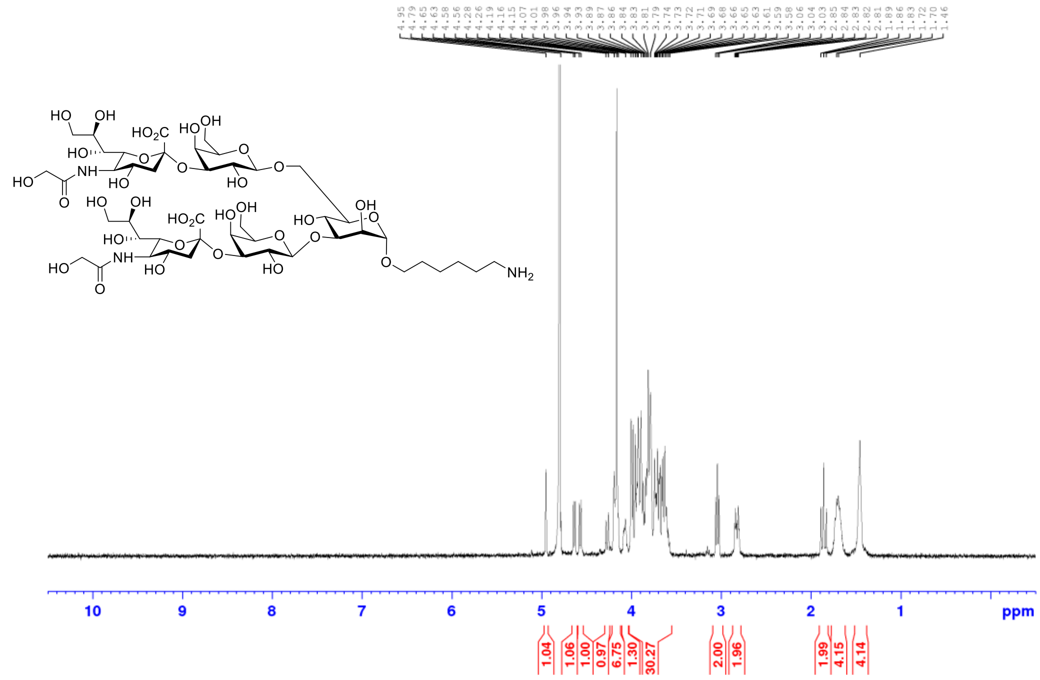

Neu5Gc-trisaccharide (SL-2)

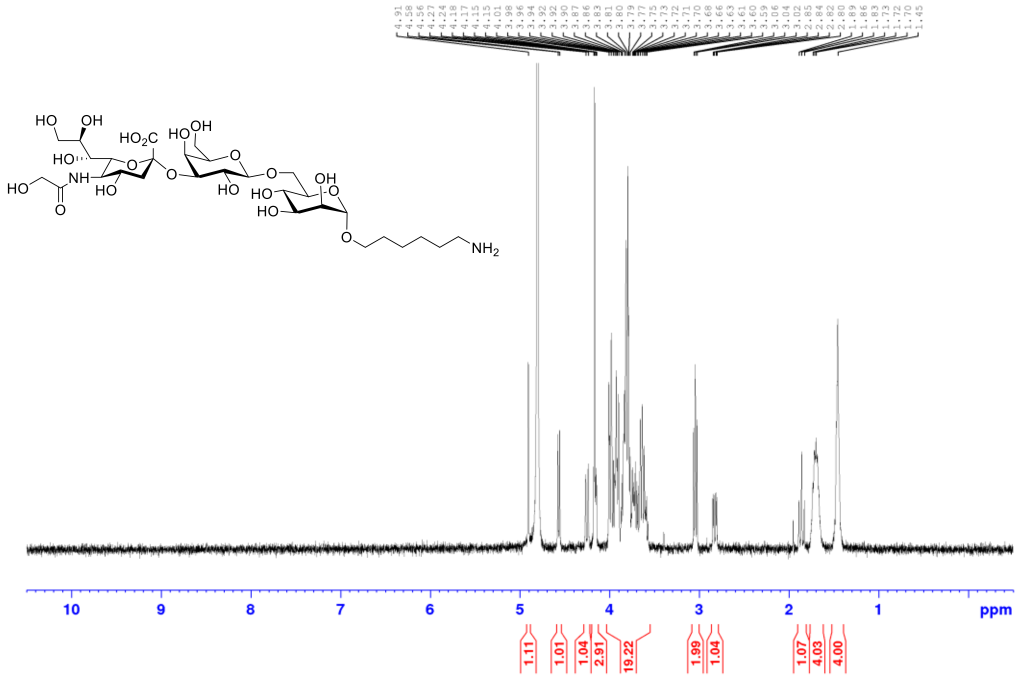

References:

1. Sun, B.; Jiang, H. Y. An efficient approach for total synthesis of aminopropyl functionalized ganglioside GM1b. *Tetrahedron Lett.* **2012**, *53* (42), 5711-5715. DOI: 10.1016/j.tetlet.2012.08.077

2. Crich, D.; Li, W. O-sialylation with N-acetyl-5-n,4-o-carbonyl-protected thiosialoside donors in dichloromethane: facile and selective cleavage of the oxazolidinone ring. *J. Org. Chem.* **2007**, *72* (7), 2387-2391. DOI: 10.1021/jo062431r

3. Farris, M. D.; De Meo, C. Application of 4,5-O,-oxazolidinone protected thiophenyl sialosyl donor to the synthesis of α-sialosides. *Tetrahedron Lett.* **2007**, *48* (7), 1225-1227. DOI: 10.1016/j.tetlet.2006.12.061

4. Ingle, A. B.; Chao, C. S.; Hung, W. C.; Mong, K. K. T. Chemical Synthesis of the O-Antigen Repeating Unit of *Escherichia coli* O86 by an N-Formylmorpholine-Modulated One-Pot Glycosylation Strategy. *Asian J. Org. Chem.* **2014**, *3* (8), 870-876. DOI: 10.1002/ajoc.201402057

5. Yu, H.; Yu, H.; Karpel, R.; Chen, X. Chemoenzymatic synthesis of CMP-sialic acid derivatives by a one-pot two-enzyme system: comparison of substrate flexibility of three microbial CMP-sialic acid synthetases. *Bioorgan. Med. Chem.* **2004**, *12* (24), 6427-6435. DOI: 10.1016/j.bmc.2004.09.030

6. Ding, L.; Yu, H.; Lau, K.; Li, Y.; Muthana, S.; Wang, J.; Chen, X. Efficient chemoenzymatic synthesis of sialyl Tn-antigens and derivatives. *Chem. Commun. (Camb)* **2011**, *47* (30), 8691-8693. DOI: 10.1039/c1cc12732b

7. Yamamoto, T.; Nakashizuka, M.; Terada, I. Cloning and expression of a marine bacterial β-galactoside α2,6-sialyltransferase gene from *Photobacterium damsela* JT0160. *J. Biochem.* **1998**, *123* (1), 94-100.

8. Sun, M.; Li, Y.; Chokhawala, H. A.; Henning, R.; Chen, X. N-Terminal 112 amino acid residues are not required for the sialyltransferase activity of Photobacterium damsela alpha2,6-sialyltransferase. *Biotechnol. Lett.* **2008**, *30* (4), 671-676. DOI: 10.1007/s10529-007-9588-y

9. Choi, Y. H.; Kim, J. H.; Park, J. H.; Lee, N.; Kim, D. H.; Jang, K. S.; Park, I. H.; Kim, B. G. Protein engineering of alpha2,3/2,6-sialyltransferase to improve the yield and productivity of in vitro sialyllactose synthesis. *Glycobiology* **2014**, *24* (2), 159-169. DOI: 10.1093/glycob/cwt092

10. Meng, X.; Yao, W.; Cheng, J.; Zhang, X.; Jin, L.; Yu, H.; Chen, X.; Wang, F.; Cao, H. Regioselective chemoenzymatic synthesis of ganglioside disialyl tetrasaccharide epitopes. *J. Am. Chem. Soc.* **2014**, *136* (14), 5205-5208. DOI: 10.1021/ja5000609

11. Komba, S.; Yamaguchi, M.; Ishida, H.; Kiso, M. 6-O-sulfo de-N-acetylsialyl Lewis X as a novel high-affinity ligand for human L-selectin: Total synthesis and structural characterization. *Biol. Chem.* **2001**, *382* (2), 233-240. DOI: Doi 10.1515/Bc.2001.030

12. Liao, H. Y.; Hsu, C. H.; Wang, S. C.; Liang, C. H.; Yen, H. Y.; Su, C. Y.; Chen, C. H.; Jan, J. T.; Ren, C. T.; Chen, C. H.; et al. Differential receptor binding affinities of influenza hemagglutinins on glycan arrays. *J. Am. Chem. Soc.* **2010**, *132* (42), 14849-14856. DOI: 10.1021/ja104657b
